# Case-matched retrieval improves textual alignment of LLM-generated radiology impressions

**DOI:** 10.1371/journal.pone.0354688

**Published:** 2026-07-31

**Authors:** Vera Sorin, Jeremy D. Collins, Lewis D. Hahn, Alex K. Bratt, Eyal Klang, Panagiotis Korfiatis

**Affiliations:** 1 Department of Radiology, Mayo Clinic College of Medicine and Science, Mayo Clinic, Rochester, Minnesota, United States of America; 2 The Windreich Department of Artificial Intelligence and Human Health, Mount Sinai Health System and the Icahn School of Medicine, Mount Sinai, New York, United States of America; Indian Institute of Technology Patna, INDIA

## Abstract

**Background:**

Radiology impressions guide clinical care. Large Language Models (LLMs)-drafted impressions can drift into generic, off-style text. Retrieval-augmented generation (RAG) enables context-aware few-shot prompting during inference.

**Methods:**

This retrospective IRB-approved study included 11,998 CT pulmonary angiography (CTPA) reports. We built a retrieval bank from 11,399 reports and reserved 599 reports for testing. GPT-4o and LLaMA 3.1-70B generated impressions from the “findings” section using three setups: zero-shot, fixed random few-shot, and dynamic retrieval-selected few-shot (top-k semantic matches; k = 3/5/10). We ran temperatures 0, 0.7, 1. We scored outputs against the original impressions with ROUGE and BERTScore F1, report mean scores with 95% confidence intervals, and tested for statistical significance using Wilcoxon signed-rank test.

**Results:**

Dynamic retrieval-based few-shot prompting outperformed zero-shot and fixed few-shot prompting across all configurations (all p < 0.05). The highest scores were observed at temperature 0 and k = 10. ROUGE-1 F1 increased to 0.44–0.47 for GPT-4o and 0.37–0.50 for LLaMA, versus 0.35–0.37 and 0.25–0.37, respectively, in zero-shot prompting. Lower temperature and larger k were associated with higher similarity scores.

**Conclusions:**

Dynamic, case-matched retrieval improved alignment of LLM-generated CTPA impressions with reference impressions on automated text-similarity metrics. Scores remained moderate, and radiologists’ verification is still required before clinical deployment.

## Introduction

Radiology impressions guide clinical care. They don’t just restate findings, they turn them into an answer to the clinical question, often the only part a referring clinician reads closely [1].

Large language models (LLMs) perform well across different NLP tasks [2]. In radiology, they are being tested for report classification [3–6], clinical note summarization [7–9], structuring free text reports [10,11], and drafting reports, summaries, and impressions [12–14].

The problem is that fluent isn’t the same as right. For impression generation, LLMs can produce output that reads well but drifts into generic phrasing, inconsistent style, or, worse, unsupported statements and incorrect interpretations [15].

Workarounds exist. Domain adaptation and fine-tuning can improve performance [16], but inference-time strategies are gaining traction because they can change behavior without retraining [17]. Retrieval-augmented generation (RAG) is one such strategy: retrieve semantically similar prior cases and use them as high-fit few-shot examples at inference time, instead of relying on static examples [18].

This study tests a RAG-based dynamic few-shot approach for generating impressions from pulmonary CT angiography (CTPA) reports. We compared this method against zero-shot prompting and fixed, randomly selected few-shot prompting.

## Methods

This retrospective study was approved by the Mayo Clinic Institutional Review Board (IRB), with a waiver of informed consent granted (approval no. 24–013325, March 10, 2025). We included CTPA reports from Mayo Clinic sites (Rochester, Arizona, Florida, and the Mayo Clinic Health System) performed between April 30, 2023, and June 17, 2024. Data were accessed for research purposes between March 15, 2025, and April 10, 2025. The authors had access to identifiable patient information during data extraction, which was handled within institutional firewalls and only for the analysis.

In total, 11,998 reports (10,681 unique patients) were analyzed. Eligible reports contained both findings and impression sections and included both structured and unstructured report formats.. Reports were randomly split at the report level: 95% (n = 11,399) formed the retrieval bank and 5% (n = 599) were held out for testing. Because the split was performed at the report rather than patient level, patients with multiple examinations could contribute more than one report across the overall dataset. The reference impressions served as the ground truth for evaluation.

**Fig 1:**
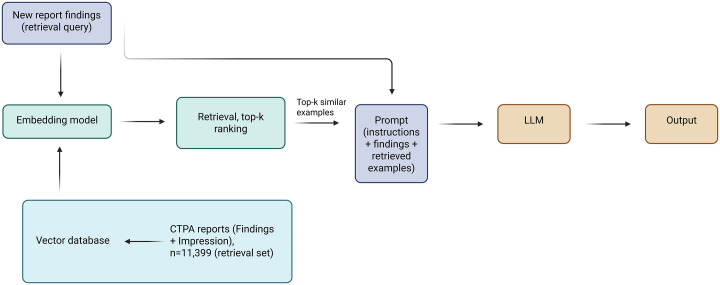
Retrieval-augmented prompting (retrieval-based few-shot) for impression generation from CT pulmonary angiography (CTPA) reports. The findings text from the new report is used as the retrieval query and embedded to retrieve the top-k most semantically similar findings–impression examples from a vector database (retrieval set). The final LLM prompt includes task instructions, the new report findings, and the retrieved examples, and the LLM generates the output impression.

RAG inserts case-matched examples at inference time [18]. We generated GPT-based embeddings for the findings text in the retrieval bank. For each test report, we embedded its findings, retrieved the top-k most similar findings from the bank, and inserted the paired findings–impression examples into the prompt as in-context demonstrations (Fig 1).

We evaluated LLaMA 3.1-70B [19] and GPT-4o [20] for impression generation from the findings section under three inference setups: zero-shot prompting; fixed few-shot prompting with 3, 5, or 10 randomly selected examples (held constant across runs); and dynamic retrieval-selected few-shot prompting with k = 3, 5, or 10. We ran temperatures 0, 0.7, and 1, using default top-P and top-K settings.

Outputs were compared to the reference impressions using ROUGE-1, ROUGE-2, ROUGE-L, and BERTScore F1 [21,22]; the full prompt is provided in the S1 File. We also performed qualitative analysis on a subset of cases to assess whether low similarity scores reflected superficial wording differences or clinically important errors, the 50 lowest-scoring generated impressions by ROUGE-L F1 for each model (overall 100 impressions) were evaluated by a radiologist. Error categories included wrong prioritization/redundant details, truncated or malformed output, hallucinated findings, overinterpretation/unsupported recommendations, omission, benign rephrasing, and other.

Statistical comparisons used the Wilcoxon signed-rank test with p < 0.05. All analyses were conducted in Python 3.11 within institutional firewalls, and each report was processed in an isolated run to prevent cross-case influence.

This study evaluated text-generation performance only; latency, throughput, compute cost, and clinical workflow feasibility were not assessed.

## Results

### Demographic characteristics

Baseline demographics for the retrieval bank and test set are summarized in Table 1. The cohorts were similar, with no significant differences.

**Table 1:** Demographic characteristics of patients in the embeddings and test datasets (n = 11,998^*^).

Characteristics	Embeddings Dataset	Test Dataset	P-value
Total No. of reports	11,399	599	
Age (yrs)			0.105
Median	66.39	65.52	
Mean	63.54 ± 17.29	62.31 ± 17.87	
Gender			0.407
Female	5,993	304	
Male	5,406	295	

* Patients with multiple CT pulmonary angiography examinations were included more than once, resulting in a total of 10,681 unique patients. Age reflects the patient’s age at the time of each radiology examination.

### Overall performance

**Fig 2:**
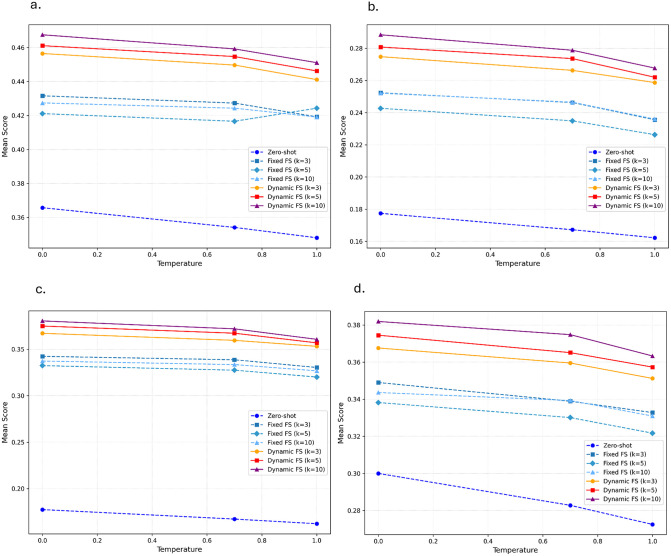
GPT-4o comparison of mean scores with dynamic, fixed and without few-shot learning across temperatures and top-k values. Mean ROUGE-1 (a), ROUGE-2 (b), ROUGE-L (c), and BERTScore (d) F1 scores for generated impressions using dynamic few-shot (FS) learning (“Dynamic FS”), zero-shot (“Zero-shot”), and fixed few-shot (“Fixed FS”), across varying temperatures (0, 0.7 and 1) and top-k retrieved documents [3,5,10]. Solid lines represent results with dynamic few-shot results, dashed lines represent zero-shot and fixed few-shot results.

**Fig 3:**
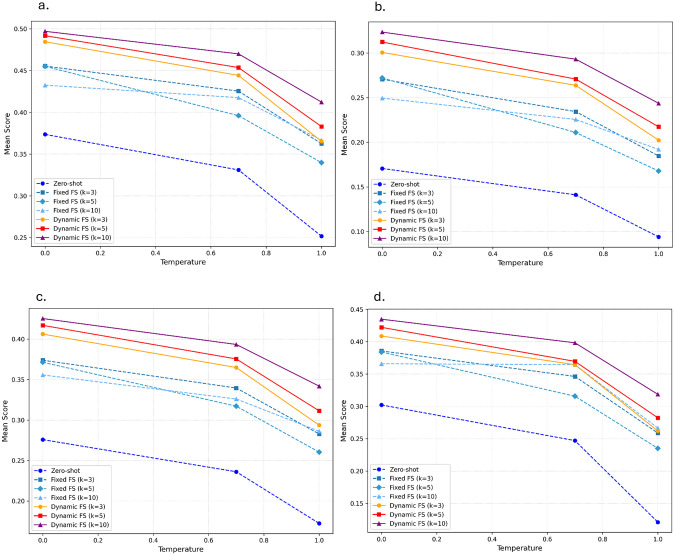
LLaMA-3.1-70B comparison of mean scores with dynamic, fixed and without few-shot learning across temperatures and top-k values. Mean ROUGE-1 (a), ROUGE-2 (b), ROUGE-L (c), and BERTScore (d) F1 scores for generated impressions using dynamic few-shot (FS) learning (“Dynamic FS”), zero-shot (“Zero-shot”), and fixed few-shot (“Fixed FS”), across varying temperatures (0, 0.7 and 1) and top-k retrieved documents [3,5,10]. Solid lines represent results with dynamic few-shot results, dashed lines represent zero-shot and fixed few-shot results.

Dynamic retrieval few-shot prompting beat both zero-shot and fixed few-shot across models, temperatures, and k on ROUGE-1/2/L and BERTScore F1 (all p < 0.05; S1 File: Supplemental Tables 1-8). Fixed few-shot also improved over zero-shot, but the gains were smaller (Figs 2–3, S1 File: Supplemental Tables 1–8).

### GPT-4o

Dynamic retrieval few-shot increased scores across metrics versus zero-shot. ROUGE-1 F1 improved from 0.348–0.366 to 0.441–0.468; ROUGE-2 from 0.162–0.177 to 0.259–0.289; ROUGE-L from 0.256–0.273 to 0.353–0.381; and BERTScore F1 from 0.273–0.300 to 0.351–0.382 (S1 File: Supplemental Tables 1-4).

### LLaMA 3.1-70B

LLaMA showed the same pattern, with larger absolute swings in several settings. ROUGE-1 F1 improved from 0.252–0.374 to 0.365–0.497; ROUGE-2 from 0.094–0.171 to 0.202–0.323; ROUGE-L from 0.172–0.276 to 0.294–0.425; and BERTScore F1 from 0.121–0.302 to 0.262–0.435 (S1 File: Supplemental Tables 5-8).

### Top-k and temperature

Across both models, performance peaked at temperature 0 with k = 10 (Figs 2–3). The gap between dynamic retrieval few-shot and zero-shot generally widened with larger k and higher temperature, with the largest gains often seen in ROUGE-L (S1 File: Supplemental Figs 1-2). GPT-4o was more temperature-sensitive than LLaMA, dropping more sharply as temperature increased from 0.7 to 1 (S1 File: Supplemental Tables 1-8). Full metric breakdowns are provided in S1 File: Supplemental Tables 1-8 and Supplemental Figs 3-74.

### Qualitative analysis

Example high- and low-scoring impressions (ROUGE-L) for both models at temperature 0 with top-10 retrieved examples are shown in Tables 2-3. In several cases, the generated impression exactly matched the reference (ROUGE-L = 1), reflecting consistent phrasing patterns in many CTPA impressions. Retrieval-selected few-shot examples helped both models capture this case-appropriate style more reliably. Lower-scoring examples illustrate where the generated impression diverged from the reference, highlighting clear targets for further prompt and retrieval refinement (Tables 2-3).

**Table 2:** GPT-4o example cases for generated impressions against the ground truth, under temperature 0 and top-10 retrieved documents.

ROUGE-L F1 for RAG Few-Shot Compared to Ground Truth	Ground Truth	Prompt	GPT-4o
1.0	1. Negative for acute pulmonary embolism. 2. No acute findings.	With RAG few-shot examples	1. Negative for acute pulmonary embolism. 2. No acute findings.
		Zero-shot	1. No evidence of pulmonary embolus. 2. No signs of right ventricular dysfunction. 3. Scattered calcified granulomas within the lungs, with no acute infiltrate or pulmonary nodule. 4. Normal mediastinal structures, including the aorta. 5. No axillary or mediastinal lymphadenopathy.
1.0	1. Negative for acute pulmonary embolism. 2. Mild bibasilar atelectasis.	With RAG few-shot examples	1. Negative for acute pulmonary embolism. 2. Mild bibasilar atelectasis.
		Zero-shot	1. No evidence of pulmonary embolus. 2. No right ventricular dysfunction. 3. Mild bibasilar atelectasis; otherwise, lungs are clear. 4. Normal mediastinal structures, including the aorta. 5. No axillary or mediastinal lymphadenopathy.
0.087	1. Negative CT angiogram of the chest.	With RAG few-shot examples	1. No pulmonary embolism. 2. No pulmonary nodule or airspace disease. 3. Heart is mildly enlarged.
		Zero-shot	1. No evidence of acute pulmonary embolism. 2. Heart size is slightly enlarged; clinical correlation recommended. 3. No significant abnormalities in the pulmonary arteries, chest wall, or upper abdomen.
0.059	Negative chest without evidence of acute pulmonary embolus.	With RAG few-shot examples	1. Negative for acute pulmonary embolism.2. No significant airway or parenchymal abnormalities to explain dyspnea.3. Small airway wall thickening and tiny mucous plugs.4. Focal linear atelectasis or scar in the posterior lingula. 5. 12 x 7 mm left thyroid nodule; recommend further evaluation with thyroid ultrasound. 6. Small hiatal hernia with herniated abdominal fat. 7. Tiny posterior left Bochdalek hernia. 8. 11 mm low attenuated lesion in the anterior liver consistent with a hepatic cyst or hemangioma. 9. Hypertrophic changes of the thoracic spine consistent with diffuse idiopathic skeletal hyperostosis (DISH).
		Zero-shot	1. No evidence of acute pulmonary embolus. 2. No significant airway or parenchymal abnormalities to explain dyspnea. 3. Incidental findings: – Small airway wall thickening and tiny mucous plugs. - Focal linear atelectasis or scar in the posterior lingula. - Calcified subcarinal and right infrahilar lymph nodes. - 12 x 7 mm left thyroid nodule. - Aortic and slight coronary artery calcification. - Small hiatal hernia with herniated abdominal fat. - Tiny posterior left Bochdalek hernia. - 11 mm low attenuated round lesion in the anterior liver, likely a hepatic cyst or hemangioma. - Hypertrophic changes of the thoracic spine consistent with diffuse idiopathic skeletal hyperostosis (DISH).

**Table 3:** LLaMA 3.1-70B example cases for generated impressions against the ground truth, under temperature 0 and top-10 retrieved documents.

ROUGE-L F1 for Few-Shot Compared to Ground Truth		Ground Truth	LLaMA 3.1-70B
1.0	With RAG few-shot examples	1. Negative for acute pulmonary embolism. 2. Mild bibasilar atelectasis.	1. Negative for acute pulmonary embolism. 2. Mild bibasilar atelectasis.
	Zero-shot		1. No evidence of pulmonary embolism or right ventricular dysfunction.2. Mild bibasilar atelectasis, likely related to patient positioning or shallow breathing during the scan. 3. No other significant findings in the chest or upper abdomen.
1.0	With RAG few-shot examples	1. Negative for acute pulmonary embolism. 2. Clear Lungs.	1. Negative for acute pulmonary embolism. 2. Clear lungs.
	Zero-shot		1. No evidence of pulmonary embolism or other significant vascular abnormalities. 2. No acute or chronic lung disease identified. 3. No pleural or pericardial effusion. 4. Normal cardiac silhouette and mediastinal structures.
0.061	With RAG few-shot examples	Negative chest CT without evidence of acute pulmonary embolus.	1. No pulmonary embolism. 2. Interval clearing of prior left lower lobe infiltrate and small left pleural effusion with residual linear atelectasis or scarring.
	Zero-shot		1. No evidence of pulmonary embolism. 2. Interval clearing of left lower lobe infiltrate and small pleural effusion, with residual linear atelectasis or scarring. 3. Incidental finding of a tiny renal papilla stone in the left kidney. 4. Incidental finding of a cortical bone island in the right scapula.

Review of the 50 lowest-scoring impressions per model showed different error profiles (S1 File: Supplemental Tables 9-12). GPT-4o failures were dominated by wrong prioritization or redundant details (37/50, 74%), whereas LLaMA 3.1-70B failures were dominated by truncated or malformed output (34/50, 68%). Hallucinations were observed in both models (GPT-4o: 5/50, 10%; LLaMA 3.1-70B: 9/50, 18%), while omissions were less common (GPT-4o: 3/50, 6%; LLaMA 3.1-70B: 4/50, 8%). Representative examples are provided in S1 File: Supplemental Table 12.

## Discussion

In this study, we evaluated whether retrieval-based dynamic few-shot prompting could generate LLM-derived CTPA impressions that more closely resemble reference impressions. Across GPT-4o and LLaMA-3.1-70B, this method consistently outperformed zero-shot and fixed few-shot prompting, showing improved alignment on automated text-similarity metrics.

An impression is not a restatement of findings; it is the clinical answer, often compressed into a few lines that clinicians actually act on [1]. That makes impression generation harder than generic summarization and more sensitive to local style, ordering, and what a radiologist chooses to foreground. Zero-shot prompting has no way to lock onto that local style. Fixed few-shot helps, but it is blunt: the examples may be irrelevant to the case at hand. Dynamic retrieval changes the few-shots from “random examples” into “this is how we talk about this kind of case”, which is exactly the anchor a model needs when the target output is short and stylistically constrained.

Lower temperature and larger k produced the strongest alignment on text similarity metrics, consistent with more deterministic generation and more exposure to highly similar exemplars. The practical takeaway is simple: inference-time settings are not cosmetic, they materially shift automated similarity scores.

In the broader literature, this sits between two common approaches. Prior work has shown that zero-shot LLMs can generate radiology impressions, but performance is variable and often evaluated with subjective human scoring [12]. Fine-tuning can push performance further by teaching domain- and institution-specific patterns, as in PET impression generation [16], but it costs data, compute, and maintenance. Retrieval-based prompting offers a third path: adapt at inference time without retraining, while keeping the model closer to local reporting patterns through matched examples. This aligns with the growing focus on optimizing inference rather than only scaling models or training [17,23]. It also complements newer agent-style pipelines that pair retrieval with additional review steps to improve outputs beyond zero-shot baselines [24].

At the same time, the evaluation frame matters. ROUGE and BERTScore quantify overlap and semantic similarity, but they do not measure clinical correctness or safety; they can miss hallucinations, unsupported claims, and harmful omissions [21,22,25]. Because both retrieval and BERTScore rely on embeddings, BERTScore gains also deserve a careful read [22]. These metrics are useful for controlled benchmarking, but they do not answer the clinical question of “is this impression right?”. We did not measure end-to-end inference latency, retrieval overhead, token or compute cost, throughput, or implementation burden in clinical workflow. Accordingly, the present results speak to text-generation alignment rather than operational feasibility in real-world deployment.

This study has several limitations. It was retrospective, limited to a single exam type (CTPA), and evaluated only two LLMs. In addition, we did not perform human evaluation of the generated impressions, so we cannot determine whether higher similarity scores corresponded to better clinical quality, correctness, or safety. Evaluation relied exclusively on automated similarity metrics. ROUGE and BERTScore are useful for benchmarking textual overlap and semantic similarity to reference impressions, but they are not measures of diagnostic accuracy or clinical correctness. They do not reliably detect negation errors, clinically important omissions, unsupported statements, or harmful hallucinations. Accordingly, the improvements reported here should be interpreted as gains in textual alignment with reference impressions rather than evidence of clinical safety, diagnostic validity, or readiness for real-world deployment. In addition, the dataset was split at the report level rather than the patient level. Because some patients contributed multiple CTPA reports, strict independence between retrieval-bank and test reports may be reduced compared with a patient-level split, and this may have modestly favored retrieval of semantically similar prior cases. Finally, we did not assess latency, throughput, compute cost, or operational feasibility in clinical workflow; therefore, these findings should not be interpreted as evidence of deployment readiness.

In conclusion, dynamic, case-matched retrieval consistently improved alignment of LLM-generated CTPA impressions with reference impressions on automated text-similarity metrics. The largest gains were seen at temperature 0 and k = 10. Further prospective work with human evaluation will be needed to determine whether these improvements translate to clinically meaningful gains.

## Supporting information

S1 File Supplementary materials. Supplemental Tables 1–12, Supplemental Figures 1–74, and the LLM impression generation prompt.
(DOCX)

## Abbreviations

BERTScore: Bidirectional Encoder Representations from Transformers Score
CT: Computed Tomography
CTA: Computed Tomography Angiography
CTPA: Computed Tomography Pulmonary Angiography
F1: A classification metric, which is based on precision/recall harmonic mean
GPT-4o: Generative Pre-trained Transformer version 4o
LLaMA: Large Language Model Meta AI
LLM: Large Language Model
NLP: Natural Language Processing
PE: Pulmonary Embolism
RAG: Retrieval-Augmented Generation
ROUGE: Recall-Oriented Understudy for Gisting Evaluation
