# Supplementary material for: Case-matched retrieval improves textual alignment of LLM-generated radiology impressions
Source: PLoS One. 2026 Jul 31;21(7):e0354688. doi: 10.1371/journal.pone.0354688 (PMC13426976; doi:10.1371/journal.pone.0354688)
Supplement: S1 File — Supplemental Tables 1–12, Supplemental Figures 1–74, and the LLM impression generation prompt. (DOCX) [file pone.0354688.s001.docx]

**Retrieval-Augmented Large Language Models for Generating Radiology Impressions from CT Pulmonary Angiography Reports**

**Supplementary Materials**

**Index**

Supplemental Table 1. GPT-4o ROUGE-1 F1 scores................................................................................................3

Supplemental Table 2. GPT-4o ROUGE-2 F1 scores................................................................................................4

Supplemental Table 3. GPT-4o ROUGE-L F1 scores................................................................................................5

Supplemental Table 4. GPT-4o BERTScore F1 scores..............................................................................................6

Supplemental Table 5. LLaMA 3.1-70B ROUGE-1 F1 scores..................................................................................7

Supplemental Table 6. LLaMA 3.1-70B ROUGE-2 F1 scores..................................................................................8

Supplemental Table 7. LLaMA 3.1-70B ROUGE-L F1 scores..................................................................................9

Supplemental Table 8. LLaMA 3.1-70B BERTScore F1 scores................................................................................10

Supplemental Table 9. Distribution of Lowest Scoring Impressions by Error Type per Model……………………11

Supplemental Table 10. Distribution by Model and Strategy………………………………………………………12

Supplemental Table 11. Distribution by Model and Top-k………………………………………………………….12

Supplemental Table 12. Representative low-scoring cases with assigned error category………………………12-15

Supplemental Figure 1. GPT-4o heatmaps of differences between dynamic few-shot and zero-shot metrics…......16

Supplemental Figure 2. LLaMA-3.1-70B heatmaps of differences between few-shot and zero-shot metrics…......17

Supplemental Figures 3-38 GPT-4o change in metrics for different temperatures and top-k................................18-53

Supplemental Figures 39-74 LLaMA-3.1-70B change in metrics for different temperatures and top-k................54-89

LLM Impression Generation Prompt.......................................................................................................................90-91

**Supplemental Table 1**. GPT-4o ROUGE-1 F1 scores across temperatures and top-k retrieved documents

| Temperature | Top-k Retrieved Documents | Mean Score with Dynamic Few-Shot [95% CI] | Mean Score Zero-Shot | Mean Score with Fixed Few-Shot | P-value | | |
| --- | --- | --- | --- | --- | --- | --- | --- |
|  |  |  |  |  | Zero-Shot vs Dynamic Few-Shot | Zero-Shot vs Fixed Few-Shot | Fixed vs. Dynamic Few-Shot |
| 0 | 3 | 0.4565 [0.445-0.468] | 0.3657 [0.355-0.377] | 0.4316 [0.420–0.444] | < 0.001 | < 0.001 | < 0.001 |
| 0 | 5 | 0.4611 [0.450-0.472] |  | 0.4211 [0.410–0.432] | < 0.001 | < 0.001 | < 0.001 |
| 0 | 10 | 0.4675 [0.456-0.479] |  | 0.4274 [0.416–0.438] | < 0.001 | < 0.001 | < 0.001 |
| 0.7 | 3 | 0.4497 [0.439-0.461] | 0.3541 [0.344–0.365] | 0.4273 [0.416–0.439] | < 0.001 | < 0.001 | < 0.001 |
| 0.7 | 5 | 0.4547 [0.444-0.466] |  | 0.4166 [0.405–0.427] | < 0.001 | < 0.001 | < 0.001 |
| 0.7 | 10 | 0.4592 [0.448-0.470] |  | 0.4243 [0.413–0.436] | < 0.001 | < 0.001 | < 0.001 |
| 1 | 3 | 0.4411 [0.430-0.452] | 0.348 [0.338–0.359] | 0.4192 [0.408–0.430] | < 0.001 | < 0.001 | < 0.001 |
| 1 | 5 | 0.4462 [0.435-0.457] |  | 0.4096 [0.408–0.430] | < 0.001 | < 0.001 | < 0.001 |
| 1 | 10 | 0.4511 [0.440-0.462] |  | 0.4191 [0.408–0.430] | < 0.001 | < 0.001 | < 0.001 |

**Supplemental Table 2**. GPT-4o ROUGE-2 F1 scores across temperatures and top-k retrieved documents

| Temperature | Top-k Retrieved Documents | Mean Score with Dynamic Few-Shot | Mean Score Zero-Shot | Mean Score with Fixed Few-Shot | P-value | | |
| --- | --- | --- | --- | --- | --- | --- | --- |
|  |  |  |  |  | Zero-Shot vs Dynamic Few-Shot | Zero-Shot vs Fixed Few-Shot | Fixed vs. Dynamic Few-Shot |
| 0 | 3 | 0.2748 [0.265–0.285] | 0.1774 [0.172–0.188] | 0.2523 [0.242–0.263] | < 0.001 | < 0.001 | 0.001 |
| 0 | 5 | 0.2808 [0.271–0.291] |  | 0.2427 [0.233–0.252] | < 0.001 | < 0.001 | < 0.001 |
| 0 | 10 | 0.2884 [0.278–0.298] |  | 0.2521 [0.242–0.262] | < 0.001 | < 0.001 | < 0.001 |
| 0.7 | 3 | 0.2663 [0.256–0.276] | 0.1672 [0.163–0.179] | 0.2463 [0.236–0.257] | < 0.001 | < 0.001 | < 0.001 |
| 0.7 | 5 | 0.2736 [0.264–0.284] |  | 0.2349 [0.225–0.244] | < 0.001 | < 0.001 | < 0.001 |
| 0.7 | 10 | 0.2788 [0.269–0.289] |  | 0.2465 [0.237–0.257] | < 0.001 | < 0.001 | < 0.001 |
| 1 | 3 | 0.2587 [0.249–0.269] | 0.1622 [0.153–0.168] | 0.2356 [0.225–0.246] | < 0.001 | < 0.001 | 0.001 |
| 1 | 5 | 0.262 [0.252–0.272] |  | 0.2263 [0.217–0.236] | < 0.001 | < 0.001 | < 0.001 |
| 1 | 10 | 0.2677 [0.258–0.278] |  | 0.236 [0.258–0.278] | < 0.001 | < 0.001 | < 0.001 |

**Supplemental Table 3**. GPT-4o ROUGE-L F1 scores across temperatures and top-k retrieved documents

| Temperature | Top-k Retrieved Documents | Mean Score with Dynamic Few-Shot | Mean Score Zero-Shot | Mean Score with Fixed Few-Shot | P-value | | |
| --- | --- | --- | --- | --- | --- | --- | --- |
|  |  |  |  |  | Zero-Shot vs Dynamic Few-Shot | Zero-Shot vs Fixed Few-Shot | Fixed vs. Dynamic Few-Shot |
| 0 | 3 | 0.3672 [0.357–0.377] | 0.2733 [0.266–0.283] | 0.3424 [0.332–0.353] | < 0.001 | < 0.001 | < 0.001 |
| 0 | 5 | 0.375 [0.365–0.385] |  | 0.3324 [0.322–0.342] | < 0.001 | < 0.001 | < 0.001 |
| 0 | 10 | 0.3805 [0.370–0.391] |  | 0.3373 [0.327–0.347] | < 0.001 | < 0.001 | < 0.001 |
| 0.7 | 3 | 0.3596 [0.349–0.370] | 0.2623 [0.253–0.269] | 0.3387 [0.328–0.349] | < 0.001 | < 0.001 | < 0.001 |
| 0.7 | 5 | 0.3673 [0.357–0.377] |  | 0.3275 [0.317–0.337] | < 0.001 | < 0.001 | < 0.001 |
| 0.7 | 10 | 0.372 [0.362–0.382] |  | 0.3335 [0.324–0.343] | < 0.001 | < 0.001 | < 0.001 |
| 1 | 3 | 0.3532 [0.343–0.363] | 0.2559 [0.245–0.261] | 0.3304 [0.320–0.341] | < 0.001 | < 0.001 | 0.001 |
| 1 | 5 | 0.3569 [0.347–0.367] |  | 0.3201 [0.310–0.330] | < 0.001 | < 0.001 | < 0.001 |
| 1 | 10 | 0.3607 [0.351–0.371] |  | 0.3268 [0.317–0.337] | < 0.001 | < 0.001 | < 0.001 |

**Supplemental Table 4.** GPT-4o BERTScore F1 scores across temperatures and top-k retrieved documents

| Temperature | Top-k Retrieved Documents | Mean Score with Dynamic Few-Shot | Mean Score Zero-Shot | Mean Score with Fixed Few-Shot | P-value | | |
| --- | --- | --- | --- | --- | --- | --- | --- |
|  |  |  |  |  | Zero-Shot vs Dynamic Few-Shot | Zero-Shot vs Fixed Few-Shot | Fixed vs. Dynamic Few-Shot |
| 0 | 3 | 0.3676 [0.356–0.379] | 0.3 [0.290–0.311] | 0.3497 [0.338–0.361] | < 0.001 | < 0.001 | 0.002 |
| 0 | 5 | 0.3745 [0.363–0.386] |  | 0.3382 [0.326–0.349] | < 0.001 | < 0.001 | < 0.001 |
| 0 | 10 | 0.3819 [0.371–0.393] |  | 0.3436 [0.332–0.355] | < 0.001 | < 0.001 | < 0.001 |
| 0.7 | 3 | 0.3595 [0.348–0.371] | 0.2828 [0.276–0.297] | 0.3389 [0.328–0.350] | < 0.001 | < 0.001 | < 0.001 |
| 0.7 | 5 | 0.3651 [0.354–0.376] |  | 0.3301 [0.319–0.341] | < 0.001 | < 0.001 | < 0.001 |
| 0.7 | 10 | 0.3748 [0.363–0.386] |  | 0.3393 [0.328–0.351] | < 0.001 | < 0.001 | < 0.001 |
| 1 | 3 | 0.3512 [0.340–0.363] | 0.2725 [0.261–0.281] | 0.3328 [0.321–0.344] | < 0.001 | < 0.001 | < 0.001 |
| 1 | 5 | 0.3573 [0.346–0.369] |  | 0.3217 [0.311–0.333] | < 0.001 | < 0.001 | < 0.001 |
| 1 | 10 | 0.3633 [0.352–0.375] |  | 0.3309 [0.320–0.342] | < 0.001 | < 0.001 | < 0.001 |

**Supplemental Table 5**. LLaMA 3.1-70B ROUGE-1 F1 scores across temperatures and top-k retrieved documents

| Temperature | Top-k Retrieved Documents | Mean Score with Dynamic Few-Shot | Mean Score Zero-Shot | Mean Score with Fixed Few-Shot | P-value | | |
| --- | --- | --- | --- | --- | --- | --- | --- |
|  |  |  |  |  | Zero-Shot vs Dynamic Few-Shot | Zero-Shot vs Fixed Few-Shot | Fixed vs. Dynamic Few-Shot |
| 0 | 3 | 0.4830 [0.4694–0.4967] | 0.3737 [0.3630–0.3841] | 0.4555 [0.4434–0.4674] | < 0.001 | < 0.001 | 0.002 |
| 0 | 5 | 0.4919 [0.4779–0.5056] |  | 0.4552 [0.4434–0.4666] | < 0.001 | < 0.001 | < 0.001 |
| 0 | 10 | 0.4971 [0.4828–0.5115] |  | 0.4326 [0.4527–0.4774] | < 0.001 | < 0.001 | < 0.001 |
| 0.7 | 3 | 0.4443 [0.4300–0.4581] | 0.3311 [0.3200–0.3426] | 0.4255 [0.4137–0.4368] | < 0.001 | < 0.001 | 0.063 |
| 0.7 | 5 | 0.4537 [0.4395–0.4676] |  | 0.4219 [0.4094–0.4340] | < 0.001 | < 0.001 | 0.002 |
| 0.7 | 10 | 0.4701 [0.4560–0.4846] |  | 0.4313 [0.4190–0.4431] | < 0.001 | < 0.001 | < 0.001 |
| 1 | 3 | 0.3654 [0.3499–0.3811] | 0.2518 [0.2405–0.2633] | 0.3626 [0.3497–0.3757] | < 0.001 | < 0.001 | 0.712 |
| 1 | 5 | 0.3831 [0.3684–0.3983] |  | 0.3467 [0.4190–0.4431] | < 0.001 | < 0.001 | < 0.001 |
| 1 | 10 | 0.4124 [0.3968–0.4280] |  | 0.3668 [0.3540–0.3797] | < 0.001 | < 0.001 | < 0.001 |

**Supplemental Table 6**. LLaMA 3.1-70B ROUGE-2 F1 scores across temperatures and top-k retrieved documents

| Temperature | Top-k Retrieved Documents | Mean Score with Dynamic Few-Shot | Mean Score Zero-Shot | Mean Score with Fixed Few-Shot | P-value | | |
| --- | --- | --- | --- | --- | --- | --- | --- |
|  |  |  |  |  | Zero-Shot vs Dynamic Few-Shot | Zero-Shot vs Fixed Few-Shot | Fixed vs. Dynamic Few-Shot |
| 0 | 3 | 0.3006 [0.2849–0.3147] | 0.1707 [0.1630–0.1787] | 0.2706 [0.2587–0.2827] | < 0.001 | < 0.001 | 0.006 |
| 0 | 5 | 0.3123 [0.2971–0.3276] |  | 0.2721 [0.2610–0.2833] | < 0.001 | < 0.001 | < 0.001 |
| 0 | 10 | 0.3234 [0.3078–0.3395] |  | 0.2495 [0.2731–0.2977] | < 0.001 | < 0.001 | < 0.001 |
| 0.7 | 3 | 0.2638 [0.2492–0.2785] | 0.1412 [0.1348–0.1501] | 0.2344 [0.2238–0.2451] | < 0.001 | < 0.001 | 0.008 |
| 0.7 | 5 | 0.2708 [0.2565–0.2856] |  | 0.211 [0.2245–0.2465] | < 0.001 | < 0.001 | < 0.001 |
| 0.7 | 10 | 0.2931 [0.2784–0.3089] |  | 0.2256 [0.2282–0.2505] | < 0.001 | < 0.001 | < 0.001 |
| 1 | 3 | 0.2024 [0.1885–0.2168] | 0.0941 [0.0895–0.1017] | 0.1847 [0.1747–0.1952] | < 0.001 | < 0.001 | 0.422 |
| 1 | 5 | 0.2174 [0.2035–0.2319] |  | 0.1679 [0.1631–0.1833] | < 0.001 | < 0.001 | < 0.001 |
| 1 | 10 | 0.2437 [0.2282–0.2601] |  | 0.192 [0.1817–0.2028] | < 0.001 | < 0.001 | < 0.001 |

**Supplemental Table 7**. LLaMA 3.1-70B ROUGE-L F1 scores across temperatures and top-k retrieved documents

| Temperature | Top-k Retrieved Documents | Mean Score with Dynamic Few-Shot | Mean Score Zero-Shot | Mean Score with Fixed Few-Shot | P-value | | |
| --- | --- | --- | --- | --- | --- | --- | --- |
|  |  |  |  |  | Zero-Shot vs Dynamic Few-Shot | Zero-Shot vs Fixed Few-Shot | Fixed vs. Dynamic Few-Shot |
| 0 | 3 | 0.4062 [0.3904–0.4194] | 0.2758 [0.2673–0.2840] | 0.3739 [0.3620–0.3856] | < 0.001 | < 0.001 | < 0.001 |
| 0 | 5 | 0.417 [0.4024–0.4319] |  | 0.3714 [0.3600–0.3826] | < 0.001 | < 0.001 | < 0.001 |
| 0 | 10 | 0.4254 [0.4100–0.4408] |  | 0.3810 [0.3690–0.3931] | < 0.001 | < 0.001 | < 0.001 |
| 0.7 | 3 | 0.3649 [0.3505–0.3791] | 0.2361 [0.2282–0.2460] | 0.3396 [0.3286–0.3507] | < 0.001 | < 0.001 | 0.021 |
| 0.7 | 5 | 0.3755 [0.3608–0.3900] |  | 0.3369 [0.3257–0.3481] | < 0.001 | < 0.001 | < 0.001 |
| 0.7 | 10 | 0.3934 [0.3789–0.4086] |  | 0.3433 [0.3318–0.3546] | < 0.001 | < 0.001 | < 0.001 |
| 1 | 3 | 0.2937 [0.2788–0.3087] | 0.1723 [0.1666–0.1829] | 0.2828 [0.2714–0.2947] | < 0.001 | < 0.001 | 0.786 |
| 1 | 5 | 0.3114 [0.2971–0.3262] |  | 0.2604 [0.2521–0.2754] | < 0.001 | < 0.001 | < 0.001 |
| 1 | 10 | 0.3419 [0.3262–0.3581] |  | 0.2861 [0.2747–0.2976] | < 0.001 | < 0.001 | < 0.001 |

**Supplemental Table 8.** LLaMA 3.1-70B BERTScore F1 scores across temperatures and top-k retrieved documents

| Temperature | Top-k Retrieved Documents | Mean Score with Dynamic Few-Shot | Mean Score Zero-Shot | Mean Score with Fixed Few-Shot | P-value | | |
| --- | --- | --- | --- | --- | --- | --- | --- |
|  |  |  |  |  | Zero-Shot vs Dynamic Few-Shot | Zero-Shot vs Fixed Few-Shot | Fixed vs. Dynamic Few-Shot |
| 0 | 3 | 0.4074 [0.3930–0.4220] | 0.3018 [0.2908–0.3127] | 0.3856 [0.3725–0.3987] | < 0.001 | < 0.001 | 0.003 |
| 0 | 5 | 0.4221 [0.4078–0.4366] |  | 0.384 [0.3709–0.3967] | < 0.001 | < 0.001 | < 0.001 |
| 0 | 10 | 0.4347 [0.4197–0.4501] |  | 0.3658 [0.3798–0.4065] | < 0.001 | < 0.001 | < 0.001 |
| 0.7 | 3 | 0.3644 [0.3494–0.3794] | 0.2463 [0.2339–0.2587] | 0.3462 [0.3334–0.3586] | < 0.001 | < 0.001 | 0.004 |
| 0.7 | 5 | 0.3695 [0.3542–0.3847] |  | 0.3419 [0.3285–0.3554] | < 0.001 | < 0.001 | 0.001 |
| 0.7 | 10 | 0.3982 [0.3828–0.4142] |  | 0.3555 [0.3422–0.3687] | < 0.001 | < 0.001 | < 0.001 |
| 1 | 3 | 0.2619 [0.2427–0.2810] | 0.1159 [0.1000–0.1320] | 0.2588 [0.2427–0.2750] | < 0.001 | < 0.001 | 0.853 |
| 1 | 5 | 0.2822 [0.2636–0.3012] |  | 0.2409 [0.2247–0.2571] | < 0.001 | < 0.001 | < 0.001 |
| 1 | 10 | 0.3185 [0.3002–0.3370] |  | 0.2663 [0.2498–0.2830] | < 0.001 | < 0.001 | < 0.001 |

**Supplemental Table 9. Distribution of Lowest Scoring Impressions by Error Type per Model**

| **Error type** | **GPT-4o, n (%)** | **LLaMA 3.1-70B, n (%)** | **Total, n (%)** |
| --- | --- | --- | --- |
| Wrong prioritization / redundant details | 37 (74%) | 1 (2%) | 38 (38%) |
| Truncated or malformed output | 0 (0%) | 34 (68%) | 34 (34%) |
| Hallucinated | 5 (10%) | 9 (18%) | 14 (14%) |
| Overinterpretation / unsupported recommendation | 5 (10%) | 0 (0%) | 5 (5%) |
| Omission | 3 (6%) | 4 (8%) | 7 (7%) |
| Benign rephrasing | 0 (0%) | 2 (4%) | 2 (2%) |
| Other | 0 (0%) | 0 (0%) | 0 (0%) |
| Total | 50 (100%) | 50 (100%) | 100 (100%) |

**Supplemental Table 10. Distribution by Model and Strategy**

| **Model** | **Strategy** | **n (%)** |
| --- | --- | --- |
| GPT-4o | Fixed few shot | 30 (30%) |
| GPT-4o | Zero shot | 20 (20%) |
| LLaMA 3.1-70B | Dynamic few shot | 24 (24%) |
| LLaMA 3.1-70B | Fixed few shot | 13 (13%) |
| LLaMA 3.1-70B | Zero shot | 13 (13%) |

**Supplemental Table 11. Distribution by Model and Top-k**

| **Model** | **Top-k** | **n (%)** |
| --- | --- | --- |
| GPT-4o | Zero-shot | 20 (20%) |
| GPT-4o | 3 | 9 (9%) |
| GPT-4o | 5 | 10 (10%) |
| GPT-4o | 10 | 11 (11%) |
| LLaMA 3.1-70B | Zero-shot | 13 (13%) |
| LLaMA 3.1-70B | 3 | 17 (17%) |
| LLaMA 3.1-70B | 5 | 12 (12%) |
| LLaMA 3.1-70B | 10 | 8 (8%) |

**Supplemental Table 12. Representative low-scoring cases with assigned error category**

| ROUGE-L F1 for RAG Few-Shot Compared to Ground Truth | Ground Truth | Generated Impression | Model | Type of Error |
| --- | --- | --- | --- | --- |
| 0.0323 | Negative exam for pulmonary arteriovenous malformation. | 1. No evidence of pulmonary embolism.  2. Normal pulmonary parenchyma and airways.  3. No pleural fluid, thickening, or mediastinal/hilar lymphadenopathy.  4. Normal heart size and pulmonary venous return, with mild bulging of the interatrial septum to the right.  5. Normal caliber thoracic aorta without atherosclerotic plaque.  6. Visualized upper abdominal structures are within normal limits. | GP4o (Fixed few-shot, Temp 0.7, Top-k = 5) | Hallucination |
| 0.037 | Exam is negative for pulmonary embolism. | 1. No pulmonary embolism identified.  2. Mild dependent bilateral groundglass opacities likely indicative of atelectasis.  3. Mild retained secretions and mucous plugging in the right bronchial tree, suggestive of potential airway disease.  4. Presence of coronary calcifications/stents, which may indicate underlying coronary artery disease.  5. No lymphadenopathy or suspicious bone lesions observed.  6. Incidental findings include hiatal hernia, atrophic pancreas, and colonic diverticula which are not currently of clinical concern.  Recommendations:  - Correlate clinical symptoms with potential airway disease due to mucous plugging and retained secretions.  - Continue current management for coronary artery disease as appropriate.  - No immediate action required for incidental findings. | GPT-4o (zero-shot, temperature = 1) | Overinterpretation |
| 0.0526 | No evidence of acute pulmonary embolus. Enlarged ascending aorta measuring 45 x 45 mm. | 1. Negative for acute pulmonary embolism.  2. Mild bronchial wall thickening is noted, which may contribute to the patient's shortness of breath.  3. No pleural fluid or thickening.  4. No mediastinal or hilar lymphadenopathy.  5. Stable incidental findings include subcentimeter right inferior thyroid nodule, mild anterior wedge deformity of the T8 vertebral body, and probable hemangioma of the T11 vertebral body. | GPT-4o (Fixed few-shot, temperature 1, top-k = 10) | Omission |
| 0 | No pulmonary embolism or other acute intrathoracic abnormality. Mild cardiomegaly, scattered coronary calcification, dilated ascending aorta up to 4.9 cm, and dilated main pulmonary artery. | ? | LLaMA 3.1-70B | Truncated or Malformed Output |

**Supplemental Figure 1. GPT-4o heatmaps of differences between dynamic few-shot and zero-shot metrics across temperatures and top-k values.** Differences between ROUGE-1 (a), ROUGE-2 (b), ROUGE-L (c), and BERTScore (d) F1 scores for generated impressions. Each cell represents the performance improvement (dynamic few-shot – zero shot) for a given combination of temperature and top-k value. Higher differences, indicated by darker blue shades.


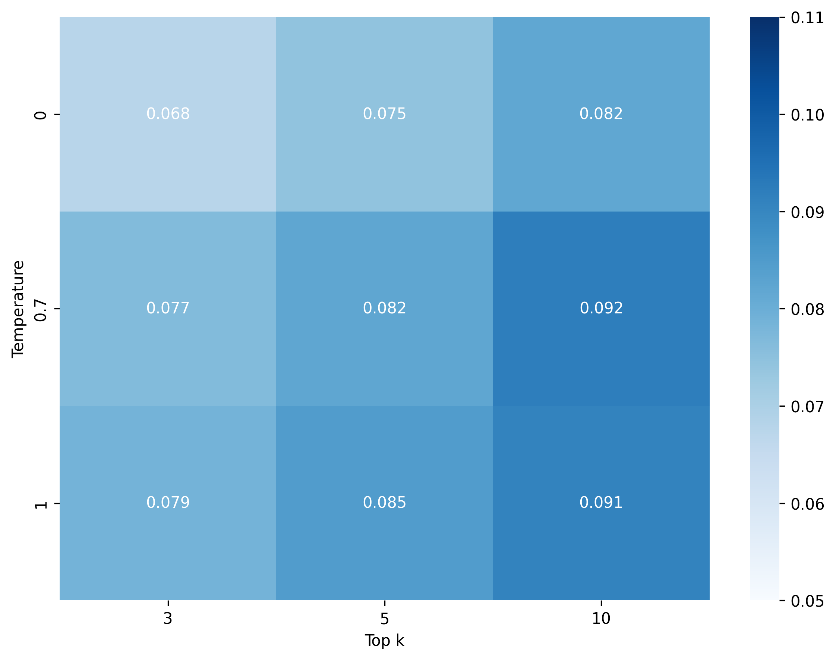

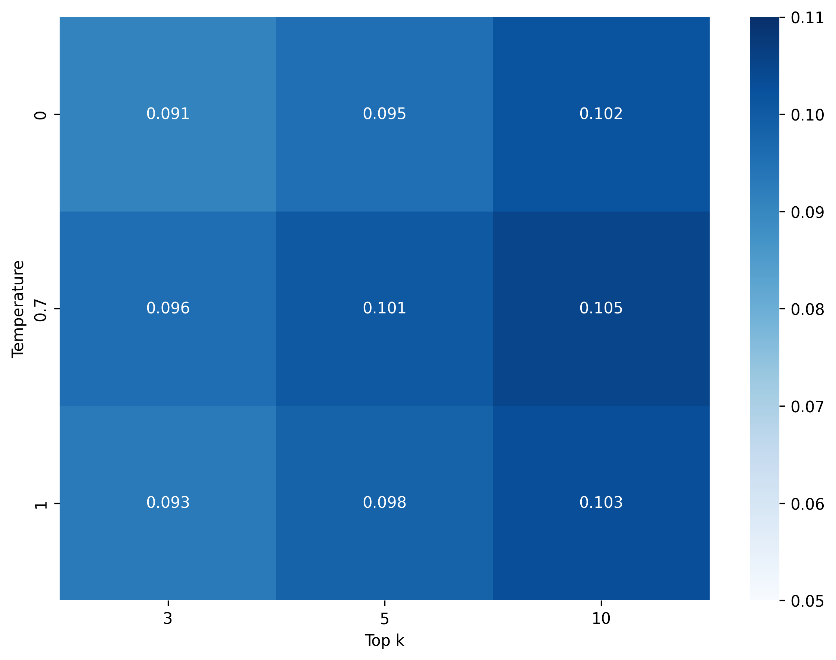

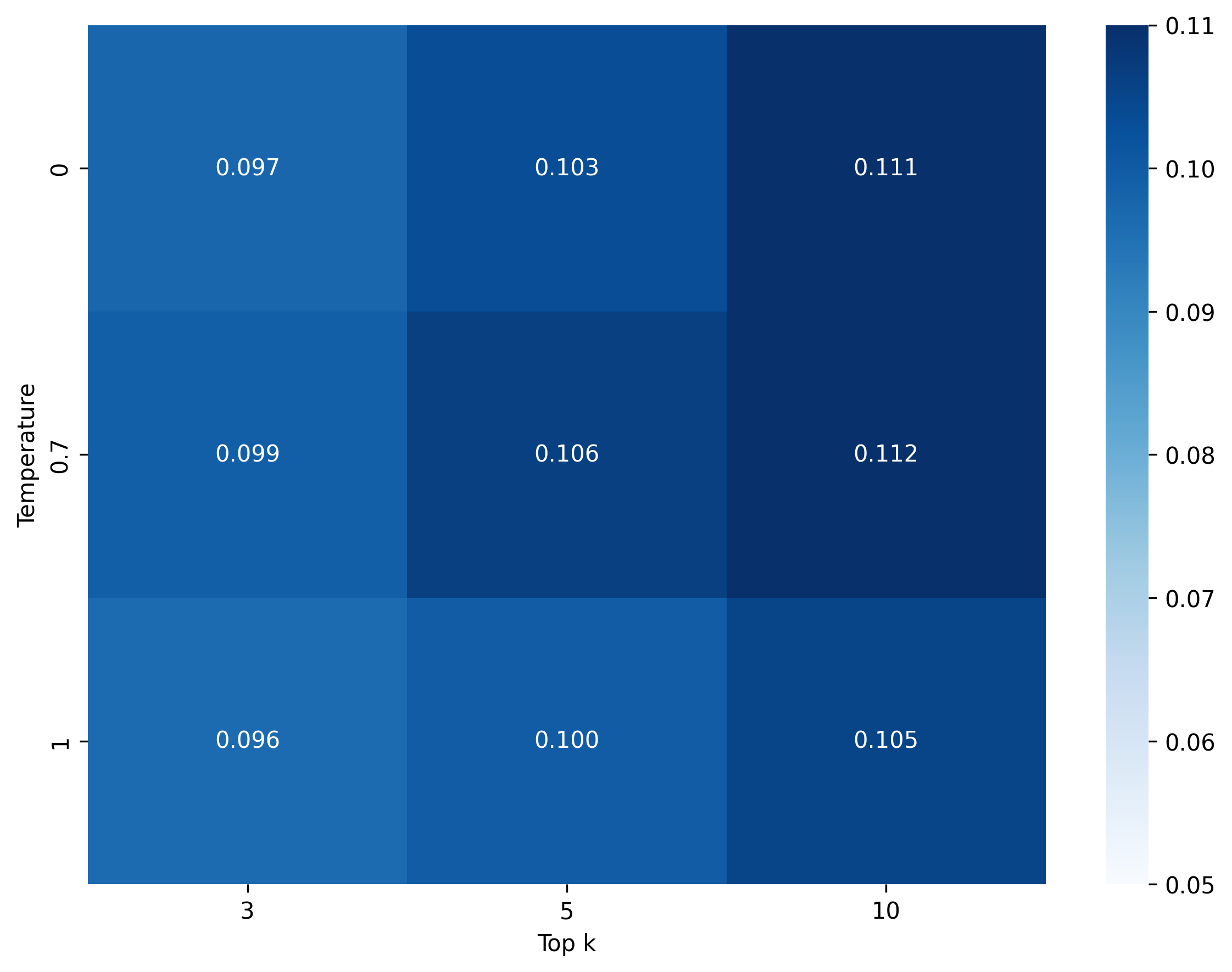

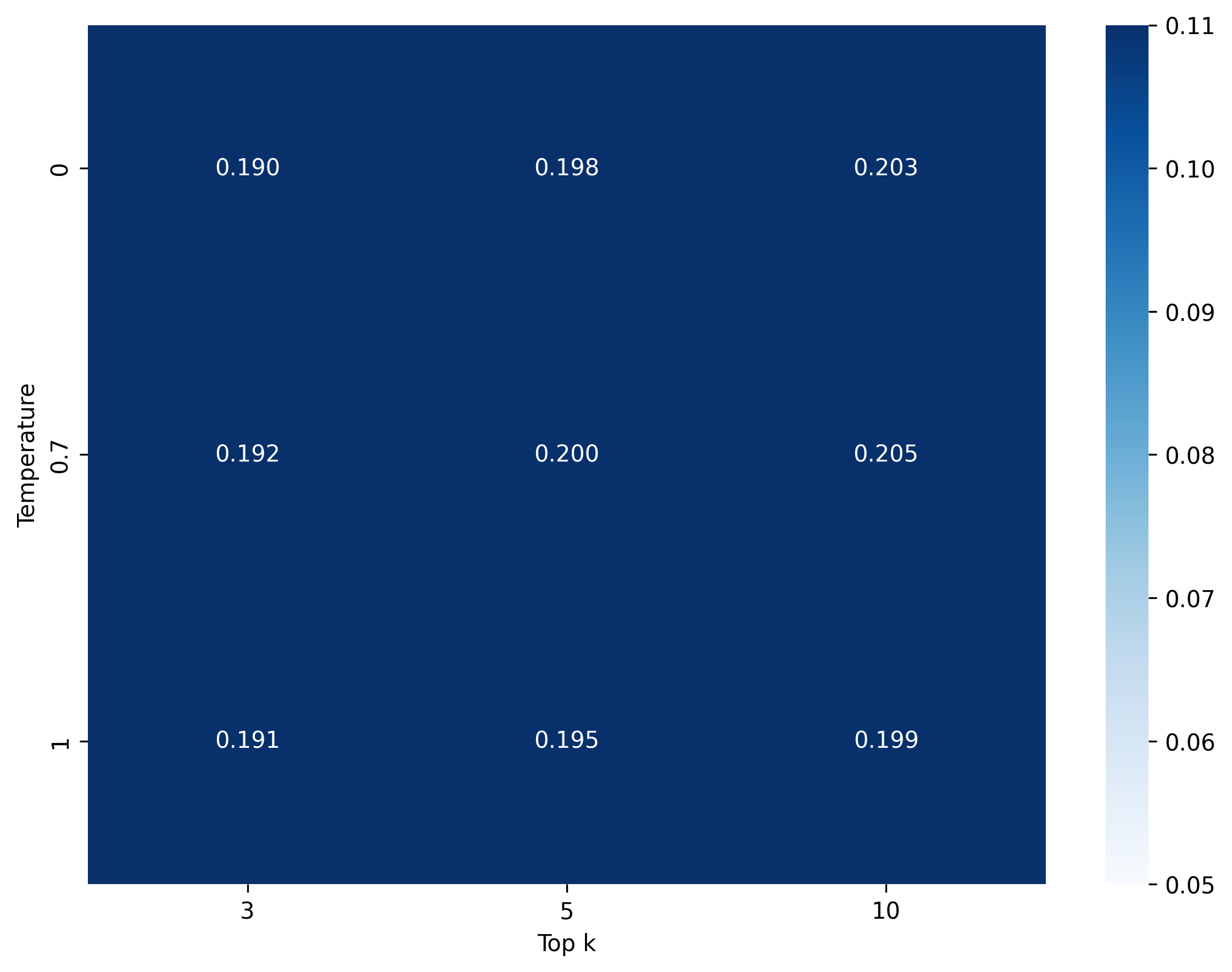


a.

b.

c.

d.

**Supplemental Figure 2. LLaMA-3.1-70B heatmaps of differences between dynamic few-shot and zero-shot metrics across temperatures and top-k values.** Differences between ROUGE-1 (a), ROUGE-2 (b), ROUGE-L (c), and BERTScore (d) F1 scores for generated impressions. Each cell represents the performance improvement (dynamic few-shot – zero shot) for a given combination of temperature and top-k value. Higher differences, indicated by darker blue shades.


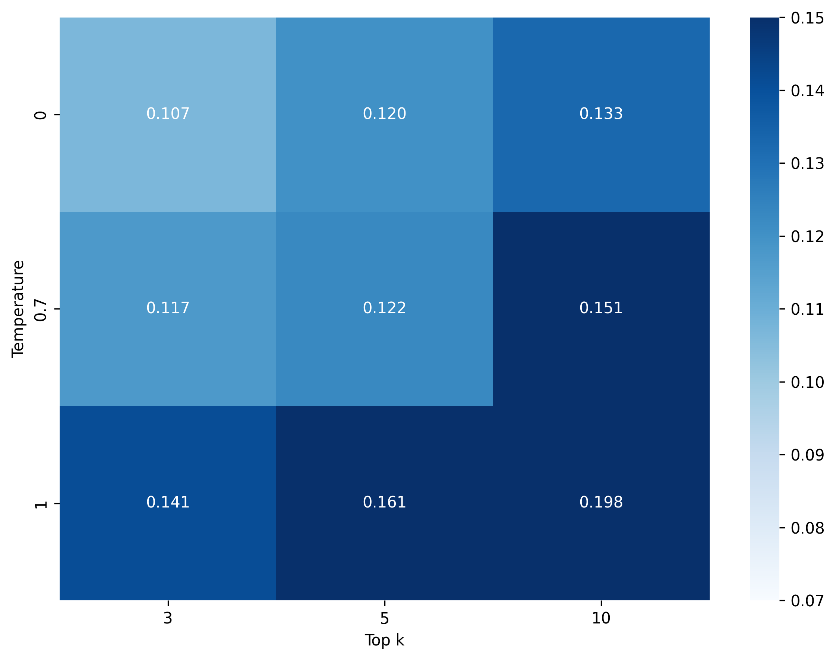

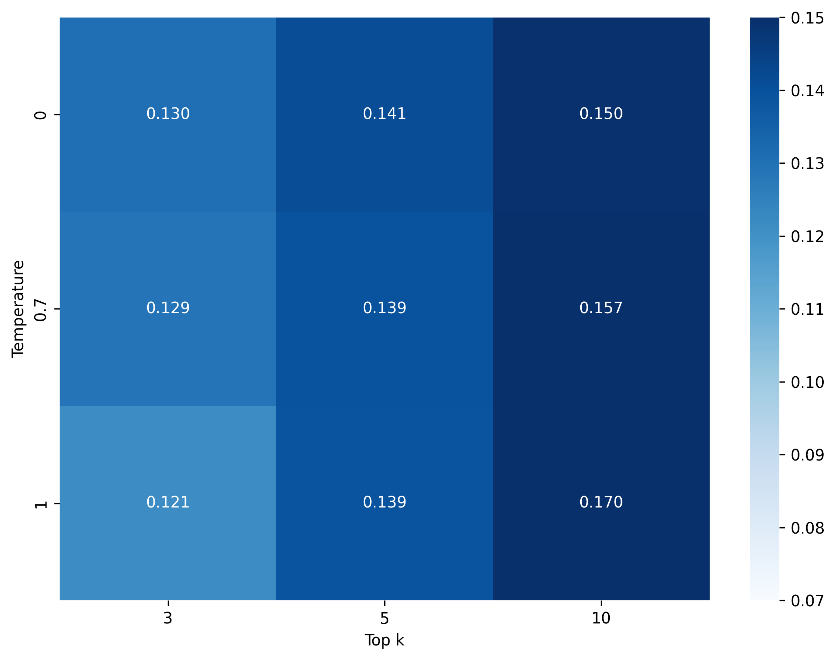

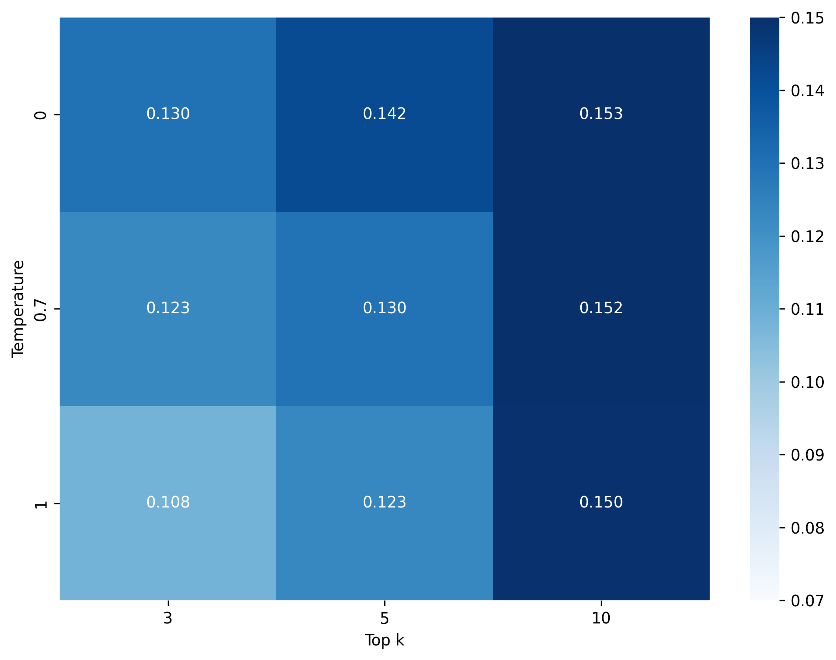

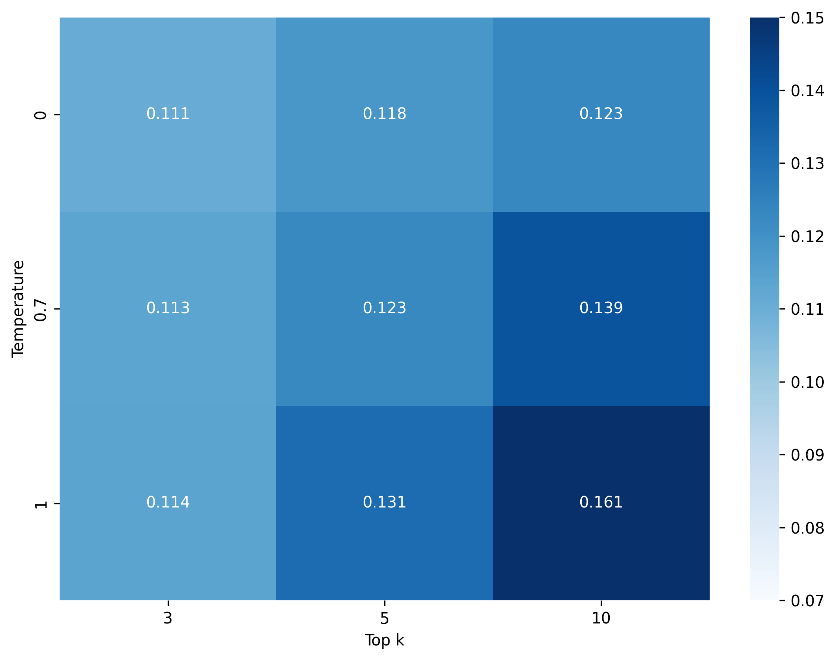


a.

b.

c.

d.

**Supplemental Figure 3. GPT-4o ROUGE-1 F1 scores with and without few-shot examples – temperature 0, top-k=3.** The lines indicate the change for each impression, where green lines indicate an increase in the score with few-shot examples, and the red lines indicate decrease.

**
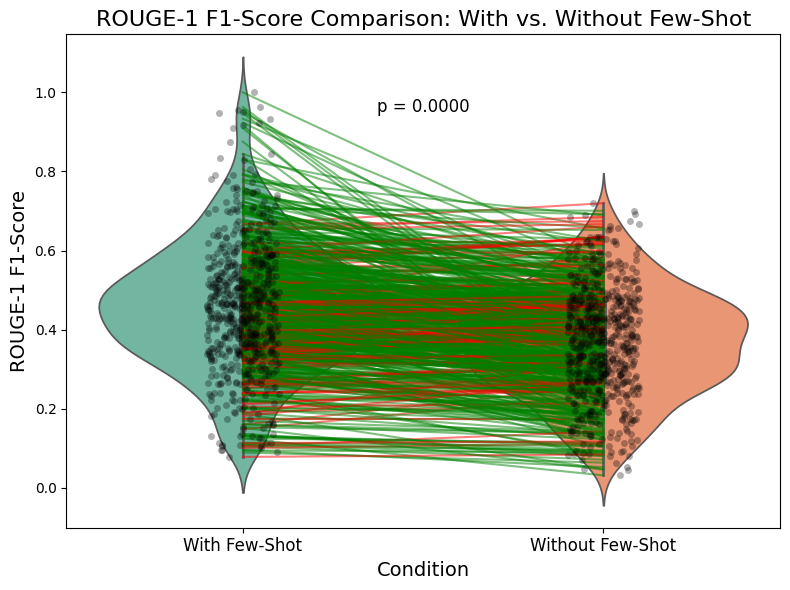
**

**Supplemental Figure 4. GPT-4o ROUGE-2 F1 scores with and without few-shot examples – temperature 0, top-k=3.** The lines indicate the change for each impression, where green lines indicate an increase in the score with few-shot examples, and the red lines indicate decrease.

**
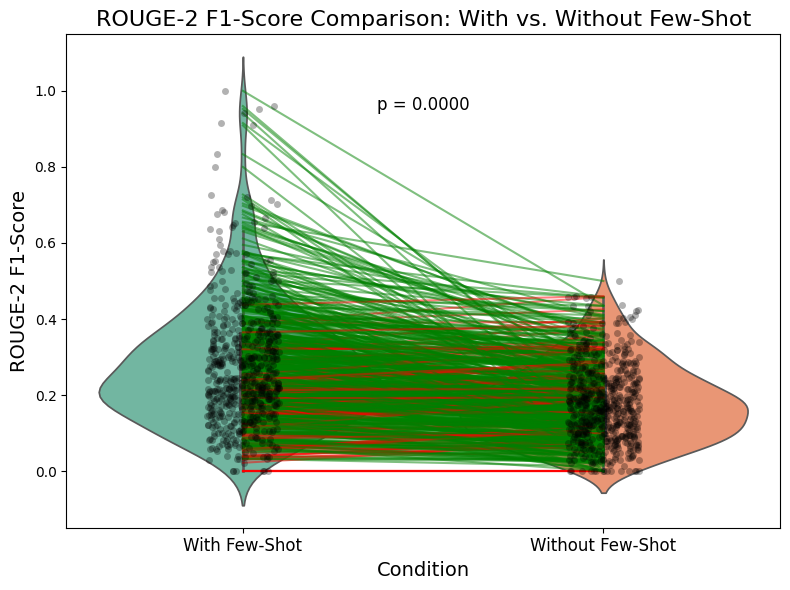
**

**Supplemental Figure 5. GPT-4o ROUGE-L F1 scores with and without few-shot examples – temperature 0, top-k=3.** The lines indicate the change for each impression, where green lines indicate an increase in the score with few-shot examples, and the red lines indicate decrease.


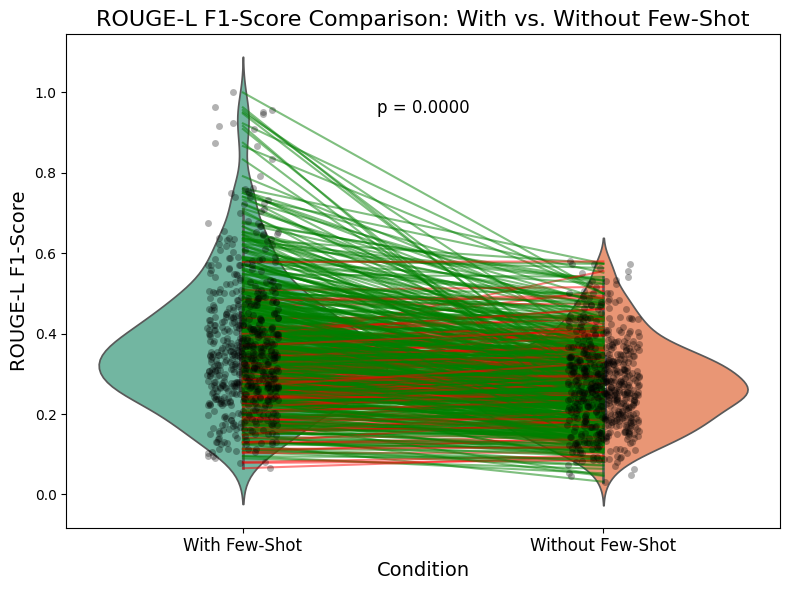


**Supplemental Figure 6. GPT-4o BERTScore F1 scores with and without few-shot examples – temperature 0, top-k=3.** The lines indicate the change for each impression, where green lines indicate an increase in the score with few-shot examples, and the red lines indicate decrease.

**
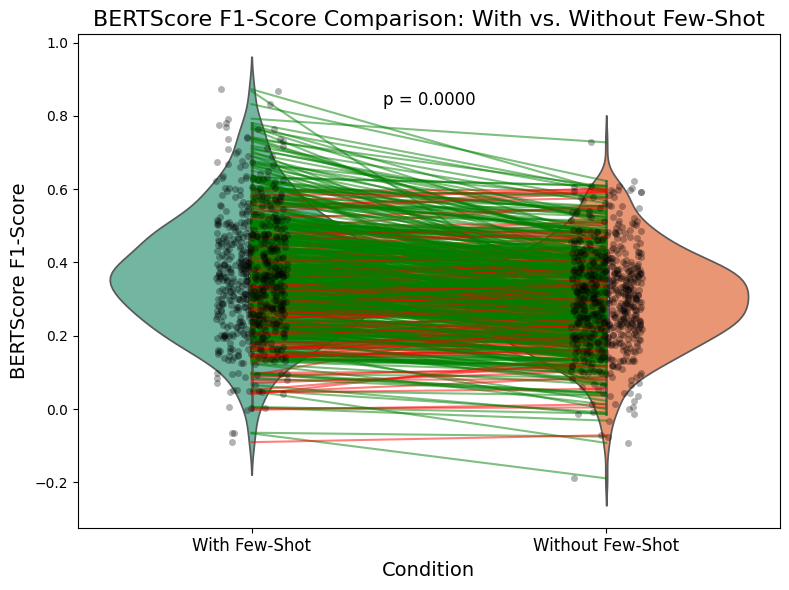
**

**Supplemental Figure 7. GPT-4o BERTScore Precision scores with and without few-shot examples – temperature 0, top-k=3.** The lines indicate the change for each impression, where green lines indicate an increase in the score with few-shot examples, and the red lines indicate decrease.

**
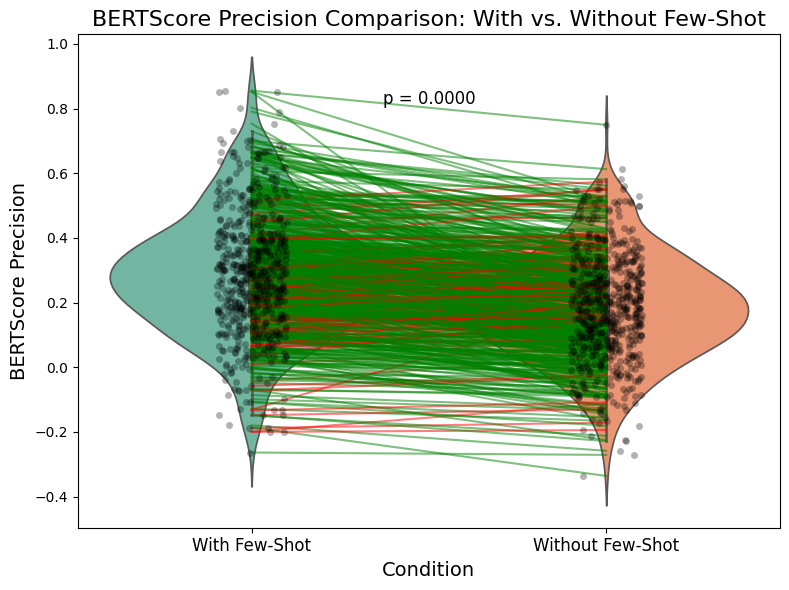
**

**Supplemental Figure 8. GPT-4o BERTScore Recall with and without few-shot examples – temperature 0, top-k=3.** The lines indicate the change for each impression, where green lines indicate an increase in the score with few-shot examples, and the red lines indicate decrease.

**
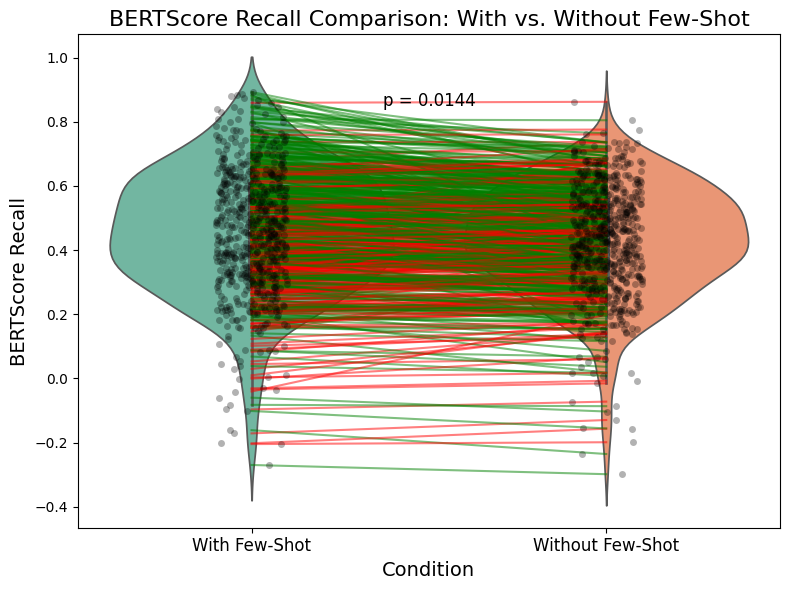
**

**Supplemental Figure 9. GPT-4o ROUGE-1 F1 scores with and without few-shot examples – temperature 0.7, top-k=3.** The lines indicate the change for each impression, where green lines indicate an increase in the score with few-shot examples, and the red lines indicate decrease.


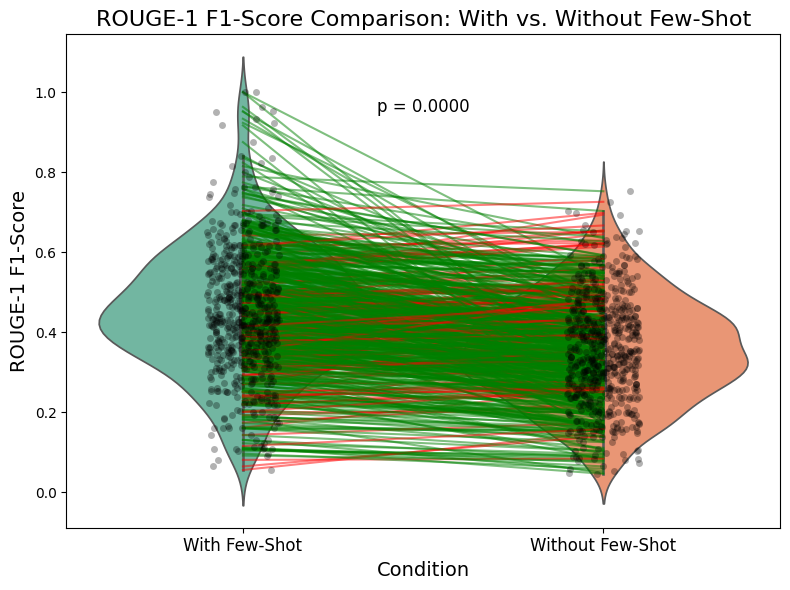


**Supplemental Figure 10. GPT-4o ROUGE-2 F1 scores with and without few-shot examples – temperature 0.7, top-k=3.** The lines indicate the change for each impression, where green lines indicate an increase in the score with few-shot examples, and the red lines indicate decrease.

**
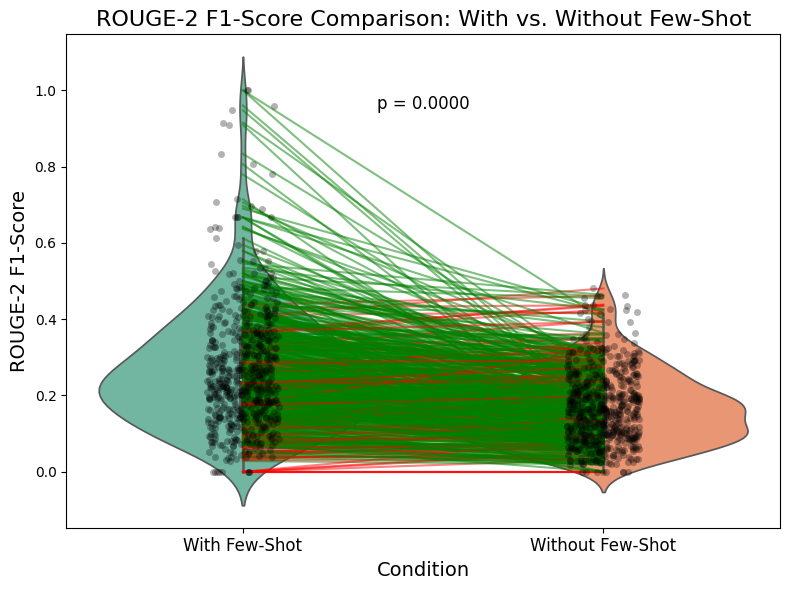
**

**Supplemental Figure 11. GPT-4o ROUGE-L F1 scores with and without few-shot examples – temperature 0.7, top-k=3.** The lines indicate the change for each impression, where green lines indicate an increase in the score with few-shot examples, and the red lines indicate decrease.

**
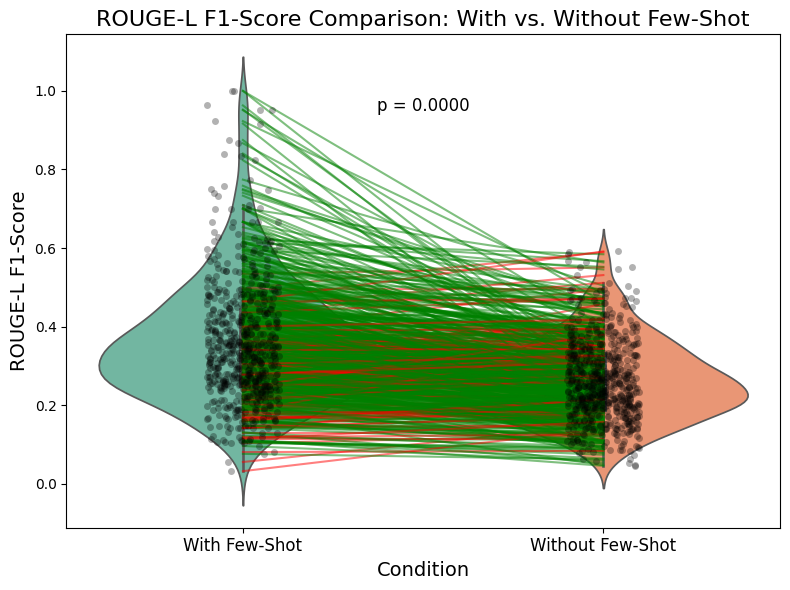
**

**Supplemental Figure 12. GPT-4o BERTScore F1 scores with and without few-shot examples – temperature 0.7, top-k=3.** The lines indicate the change for each impression, where green lines indicate an increase in the score with few-shot examples, and the red lines indicate decrease.

**
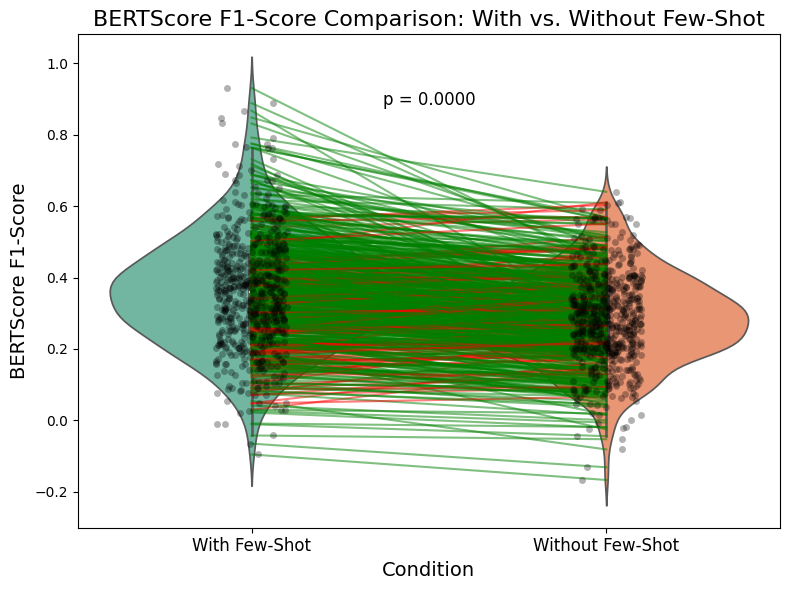
**

**Supplemental Figure 13. GPT-4o BERTScore Precision scores with and without few-shot examples – temperature 0.7, top-k=3.** The lines indicate the change for each impression, where green lines indicate an increase in the score with few-shot examples, and the red lines indicate decrease.

**
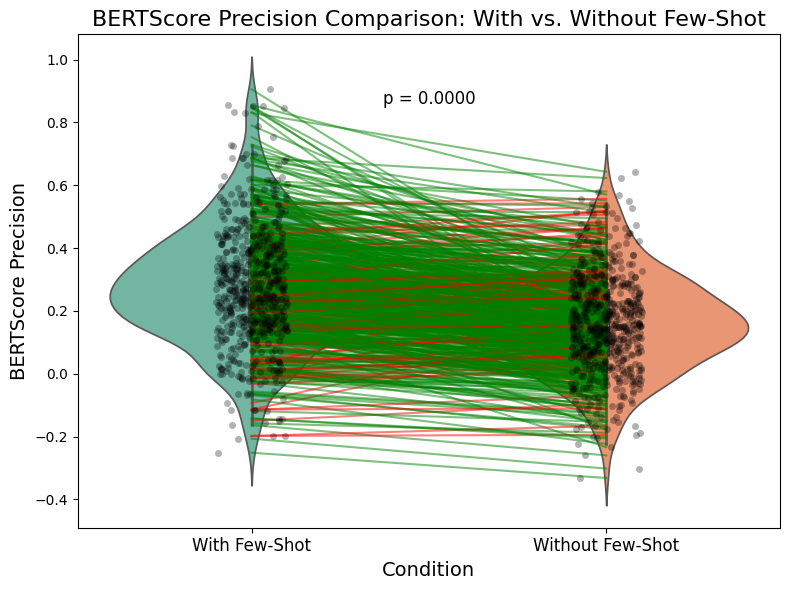
**

**Supplemental Figure 14. GPT-4o BERTScore Recall scores with and without few-shot examples – temperature 0.7, top-k=3.** The lines indicate the change for each impression, where green lines indicate an increase in the score with few-shot examples, and the red lines indicate decrease.

**
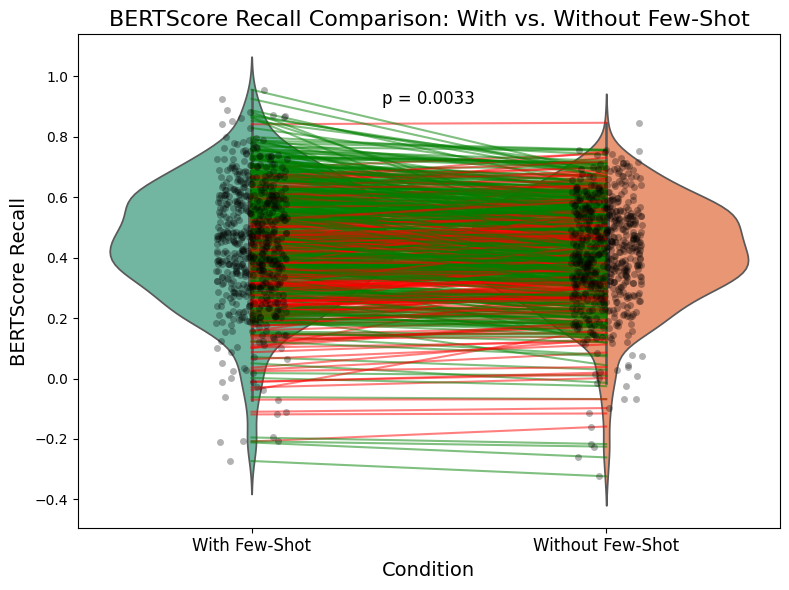
**

**Supplemental Figure 15. GPT-4o ROUGE-1 F1 scores with and without few-shot examples – temperature 1, top-k=3.** The lines indicate the change for each impression, where green lines indicate an increase in the score with few-shot examples, and the red lines indicate decrease.

**
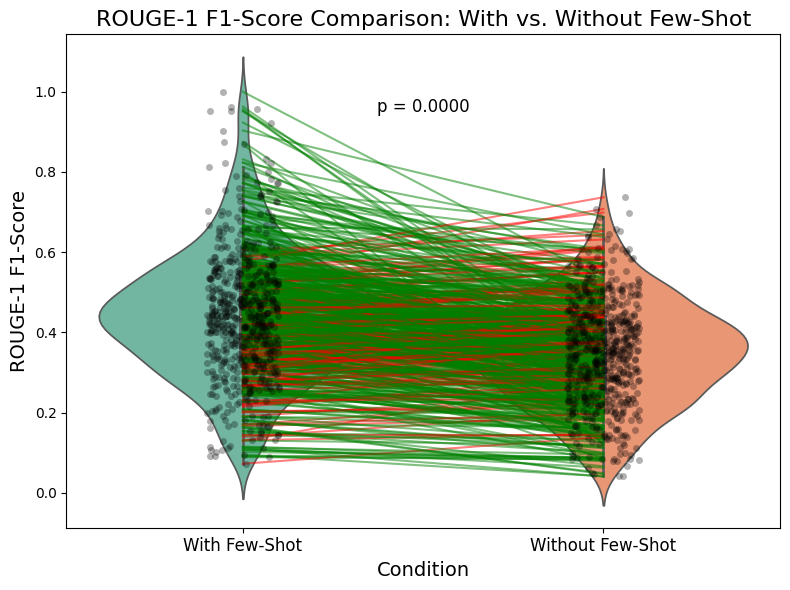
**

**Supplemental Figure 16. GPT-4o ROUGE-2 F1 scores with and without few-shot examples – temperature 1, top-k=3.** The lines indicate the change for each impression, where green lines indicate an increase in the score with few-shot examples, and the red lines indicate decrease.

**
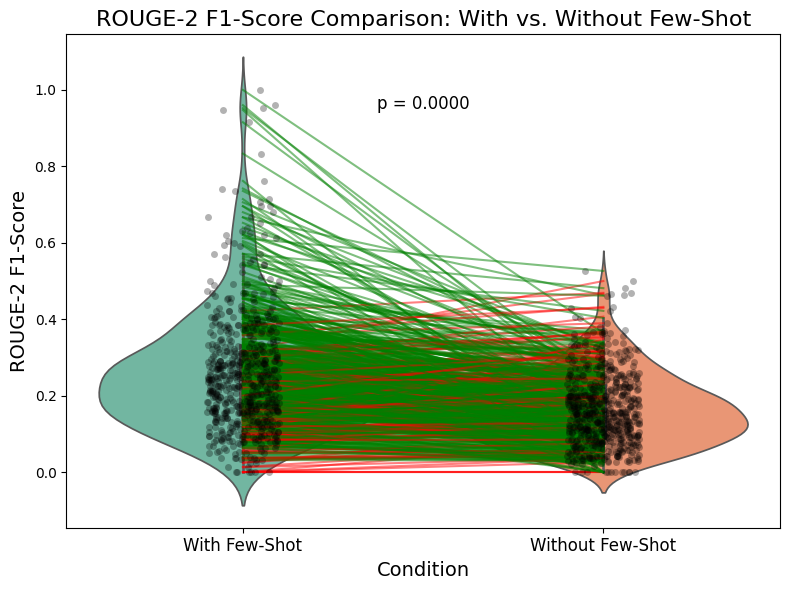
**

**Supplemental Figure 17. GPT-4o ROUGE-L F1 scores with and without few-shot examples – temperature 1, top-k=3.** The lines indicate the change for each impression, where green lines indicate an increase in the score with few-shot examples, and the red lines indicate decrease.

**
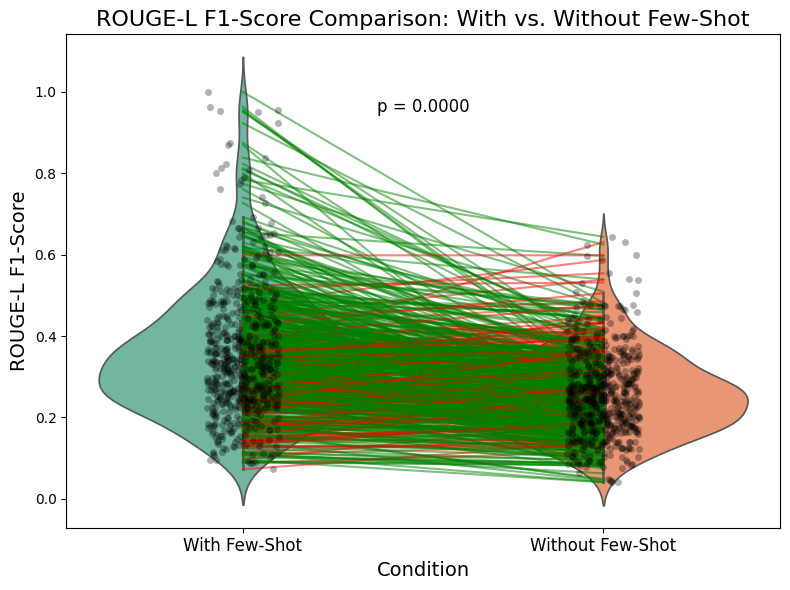
**

**Supplemental Figure 18. GPT-4o BERTScore F1 scores with and without few-shot examples – temperature 1, top-k=3.** The lines indicate the change for each impression, where green lines indicate an increase in the score with few-shot examples, and the red lines indicate decrease.

**
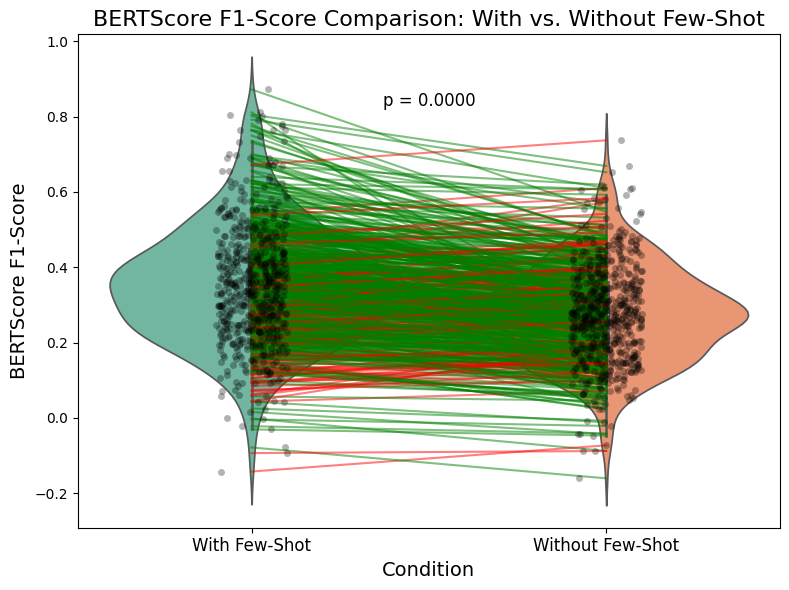
**

**Supplemental Figure 19. GPT-4o BERTScore Precision scores with and without few-shot examples – temperature 1, top-k=3.** The lines indicate the change for each impression, where green lines indicate an increase in the score with few-shot examples, and the red lines indicate decrease.


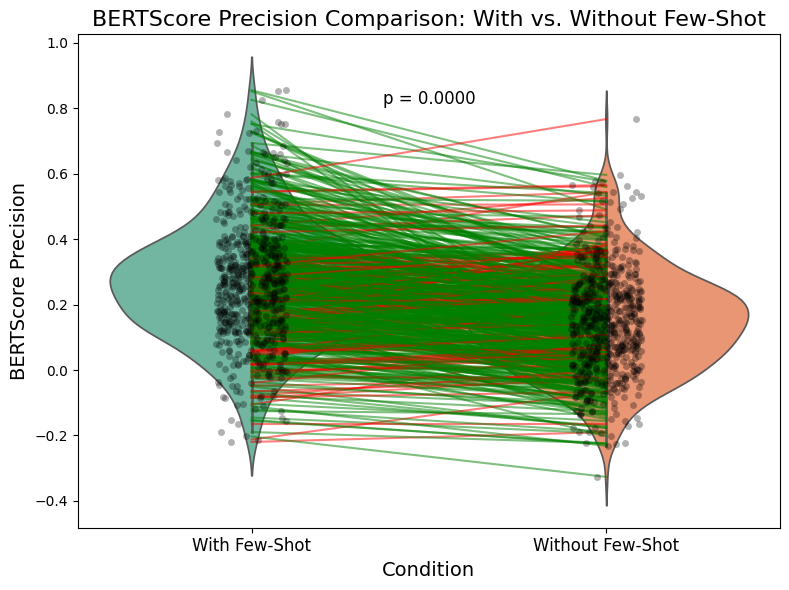


**Supplemental Figure 20. GPT-4o BERTScore Recall scores with and without few-shot examples – temperature 1, top-k=3.** The lines indicate the change for each impression, where green lines indicate an increase in the score with few-shot examples, and the red lines indicate decrease.

**
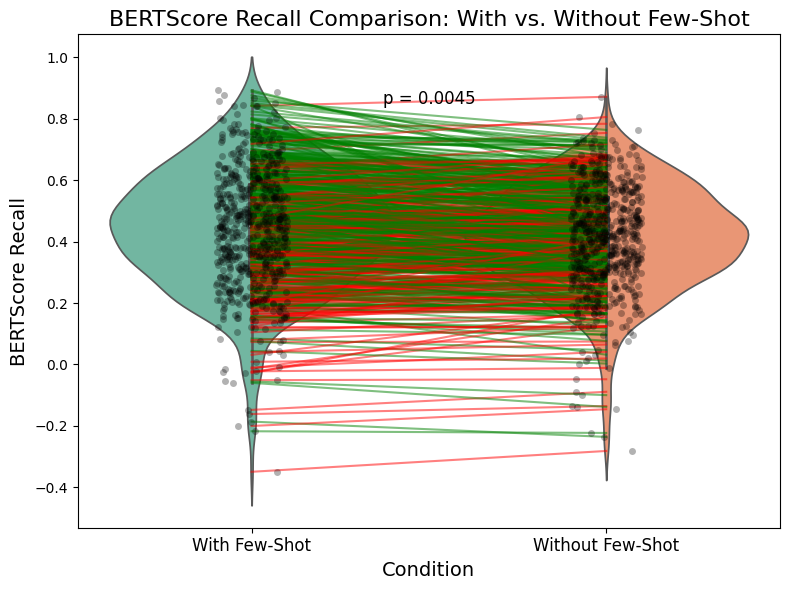
**

**Supplemental Figure 21. GPT-4o ROUGE-1 F1 scores with and without few-shot examples – temperature 0, top-k=10.** The lines indicate the change for each impression, where green lines indicate an increase in the score with few-shot examples, and the red lines indicate decrease.


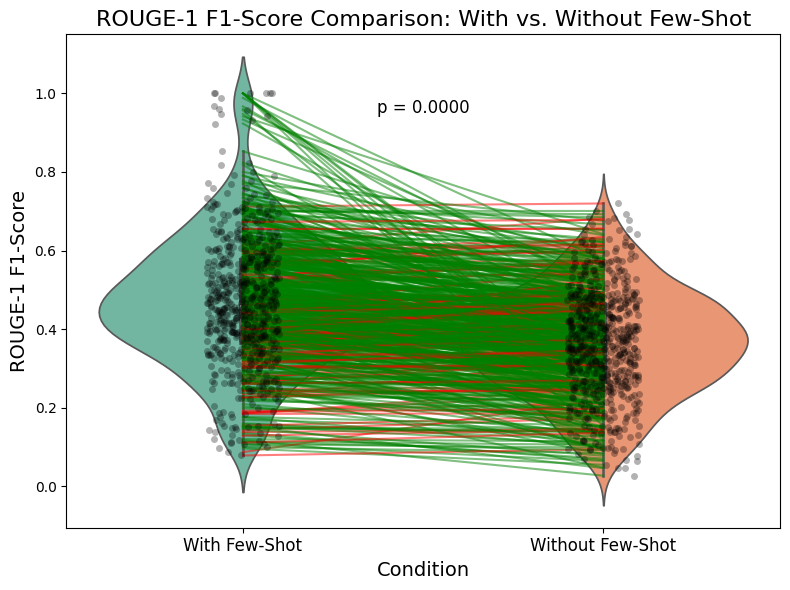


**Supplemental Figure 22. GPT-4o ROUGE-2 F1 scores with and without few-shot examples – temperature 0, top-k=10.** The lines indicate the change for each impression, where green lines indicate an increase in the score with few-shot examples, and the red lines indicate decrease.

**
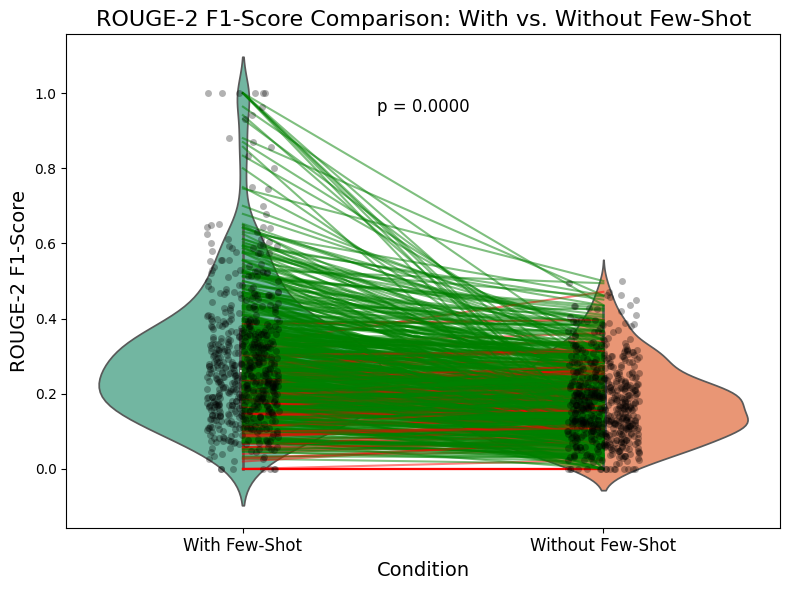
**

**Supplemental Figure 23. GPT-4o ROUGE-L F1 scores with and without few-shot examples – temperature 0, top-k=10.** The lines indicate the change for each impression, where green lines indicate an increase in the score with few-shot examples, and the red lines indicate decrease.


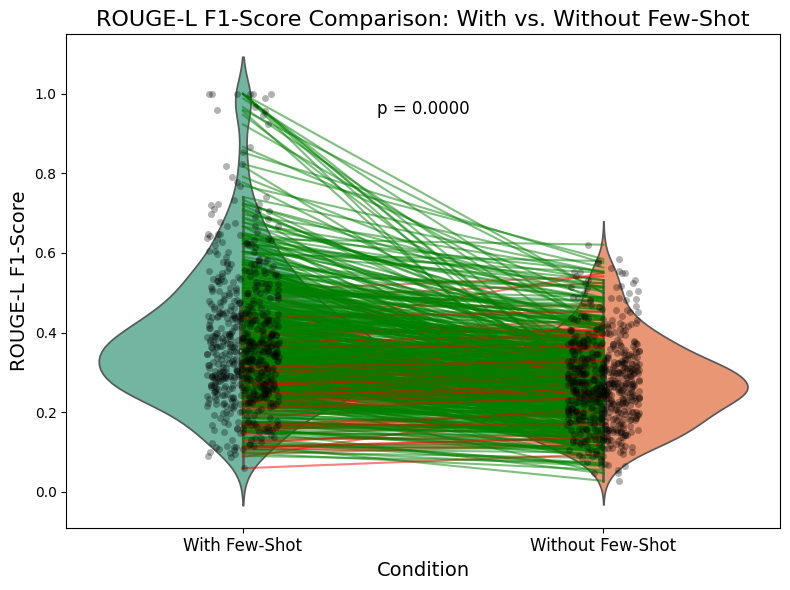


**Supplemental Figure 24. GPT-4o BERTScore F1 scores with and without few-shot examples – temperature 0, top-k=10.** The lines indicate the change for each impression, where green lines indicate an increase in the score with few-shot examples, and the red lines indicate decrease.

**
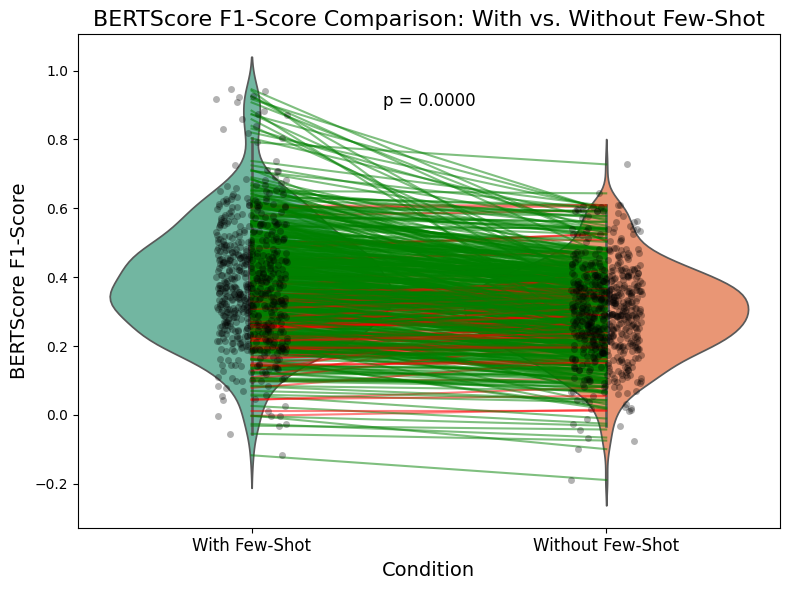
**

**Supplemental Figure 25. GPT-4o BERTScore Precision scores with and without few-shot examples – temperature 0, top-k=10.** The lines indicate the change for each impression, where green lines indicate an increase in the score with few-shot examples, and the red lines indicate decrease.


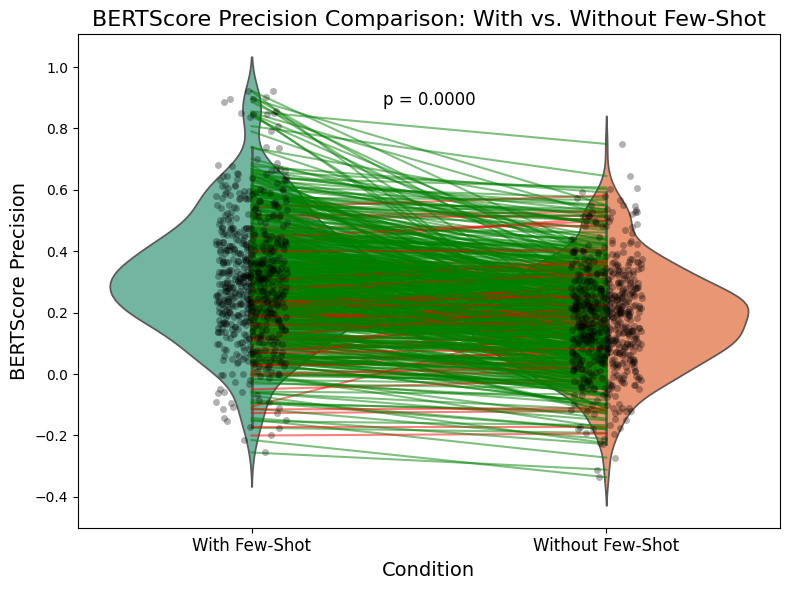


**Supplemental Figure 26. GPT-4o BERTScore Recall with and without few-shot examples – temperature 0, top-k=10.** The lines indicate the change for each impression, where green lines indicate an increase in the score with few-shot examples, and the red lines indicate decrease.

**
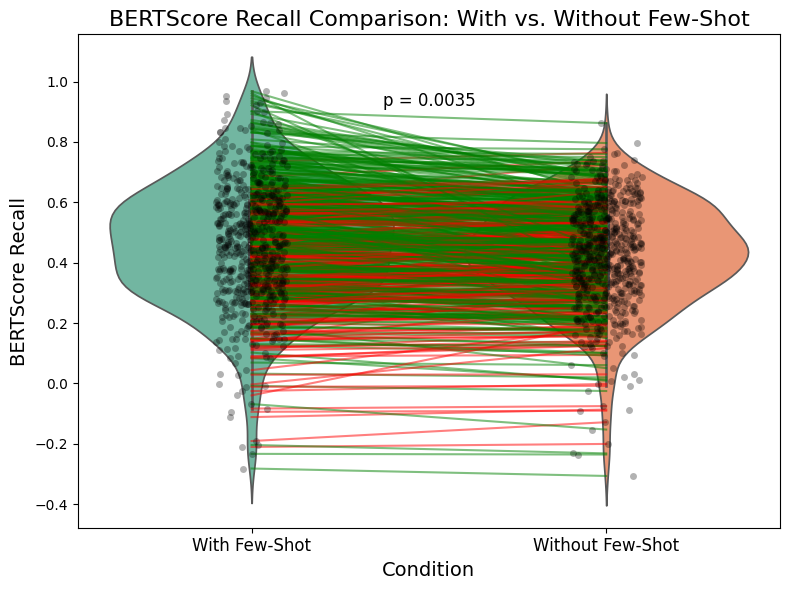
**

**Supplemental Figure 27. GPT-4o ROUGE-1 F1 scores with and without few-shot examples – temperature 0.7, top-k=10.** The lines indicate the change for each impression, where green lines indicate an increase in the score with few-shot examples, and the red lines indicate decrease.

**
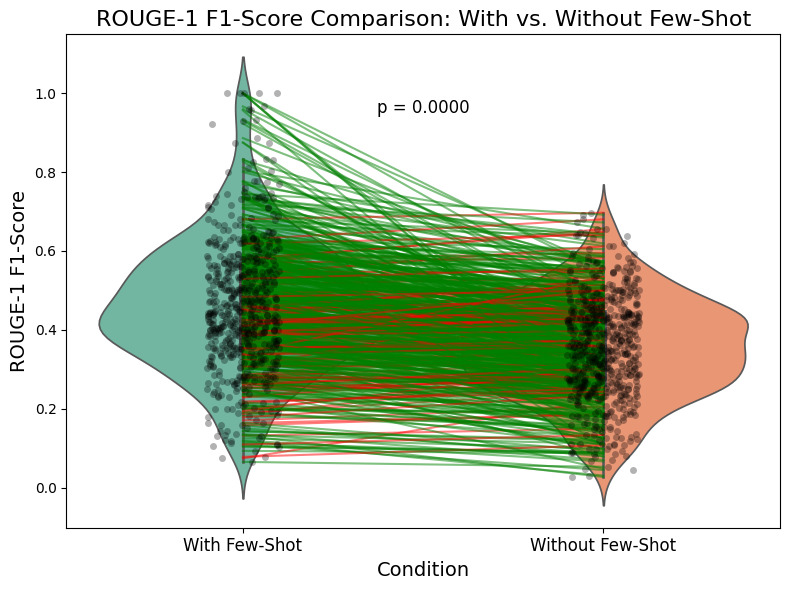
**

**Supplemental Figure 28. GPT-4o ROUGE-2 F1 scores with and without few-shot examples – temperature 0.7, top-k=10.** The lines indicate the change for each impression, where green lines indicate an increase in the score with few-shot examples, and the red lines indicate decrease.

**
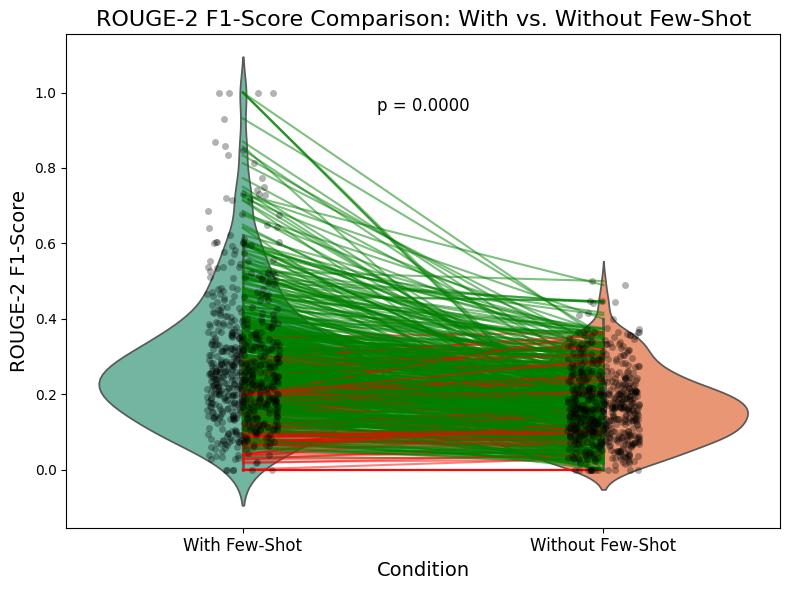
**

**Supplemental Figure 29. GPT-4o ROUGE-L F1 scores with and without few-shot examples – temperature 0.7, top-k=10.** The lines indicate the change for each impression, where green lines indicate an increase in the score with few-shot examples, and the red lines indicate decrease.

**
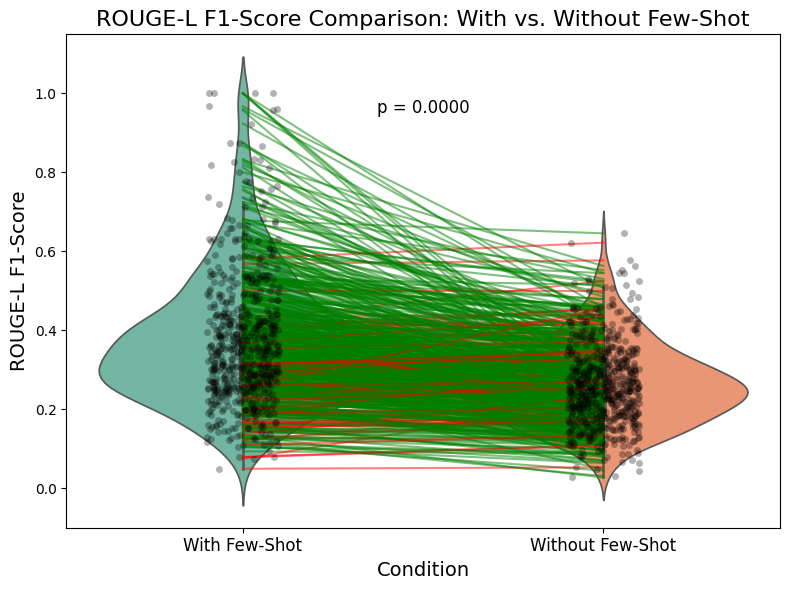
**

**Supplemental Figure 30. GPT-4o BERTScore F1 scores with and without few-shot examples – temperature 0.7, top-k=10.** The lines indicate the change for each impression, where green lines indicate an increase in the score with few-shot examples, and the red lines indicate decrease.

**
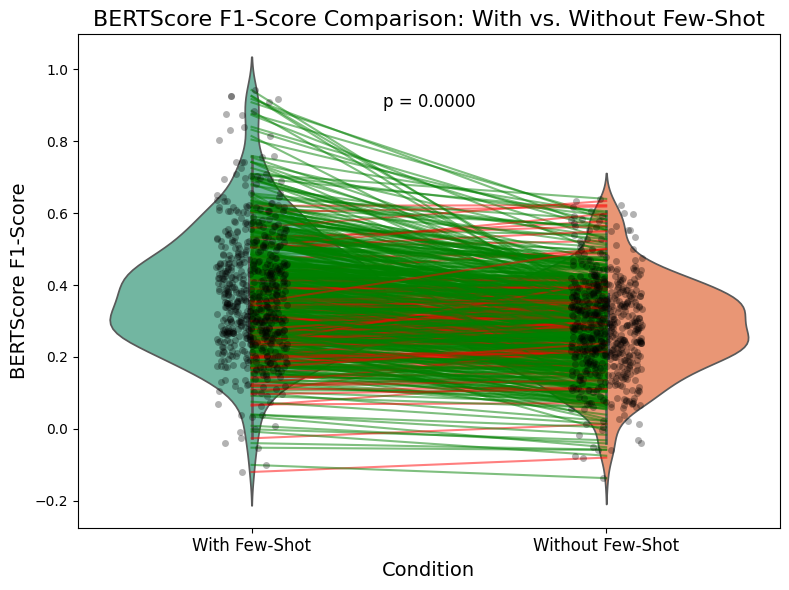
**

**Supplemental Figure 31. GPT-4o BERTScore Precision scores with and without few-shot examples – temperature 0.7, top-k=10.** The lines indicate the change for each impression, where green lines indicate an increase in the score with few-shot examples, and the red lines indicate decrease.


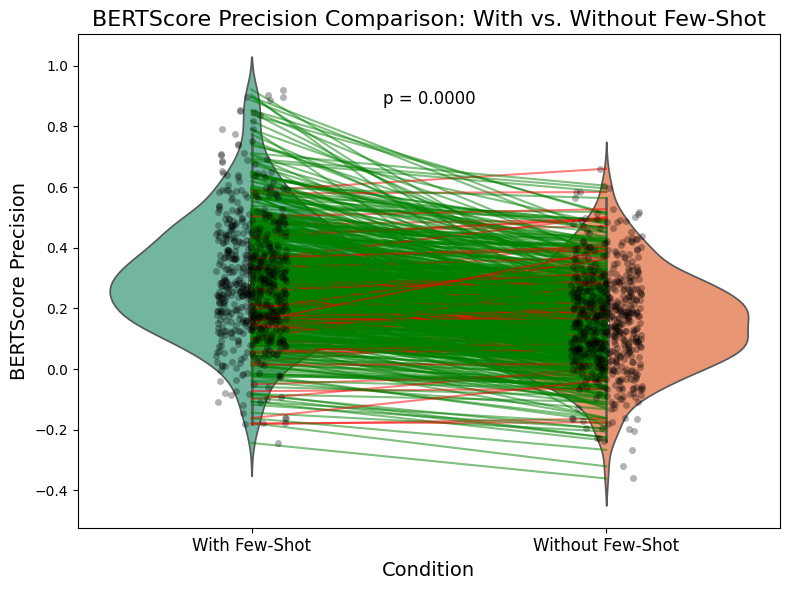


**Supplemental Figure 32. GPT-4o BERTScore Recall scores with and without few-shot examples – temperature 0.7, top-k=10.** The lines indicate the change for each impression, where green lines indicate an increase in the score with few-shot examples, and the red lines indicate decrease.

**
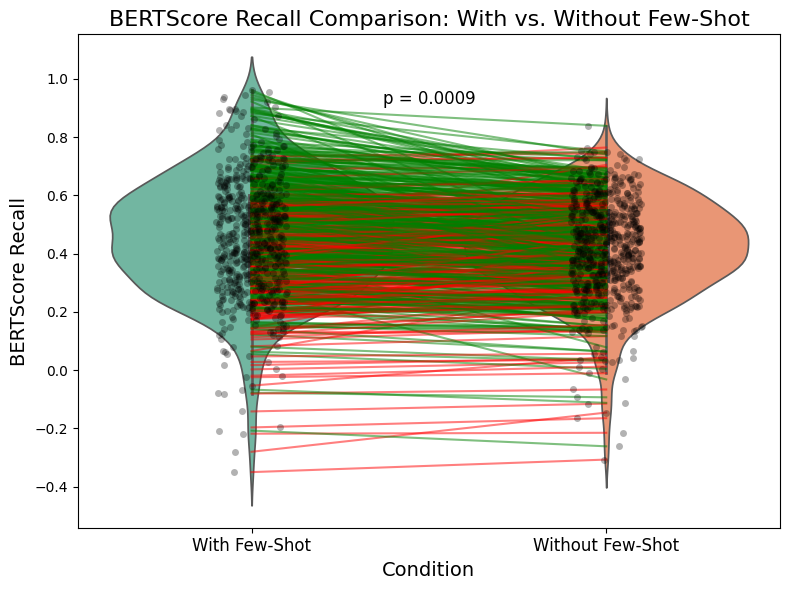
**

**Supplemental Figure 33. GPT-4o ROUGE-1 F1 scores with and without few-shot examples – temperature 1, top-k=10.** The lines indicate the change for each impression, where green lines indicate an increase in the score with few-shot examples, and the red lines indicate decrease.


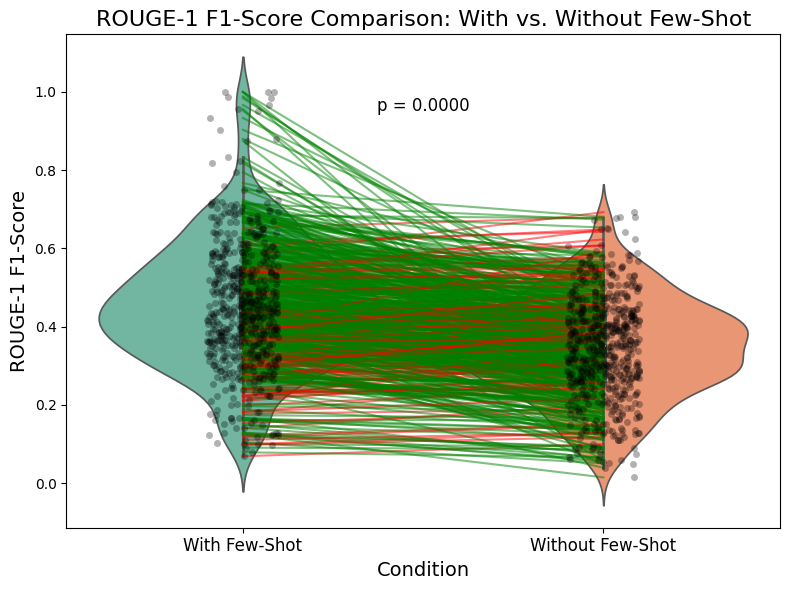


**Supplemental Figure 34. GPT-4o ROUGE-2 F1 scores with and without few-shot examples – temperature 1, top-k=10.** The lines indicate the change for each impression, where green lines indicate an increase in the score with few-shot examples, and the red lines indicate decrease.

**
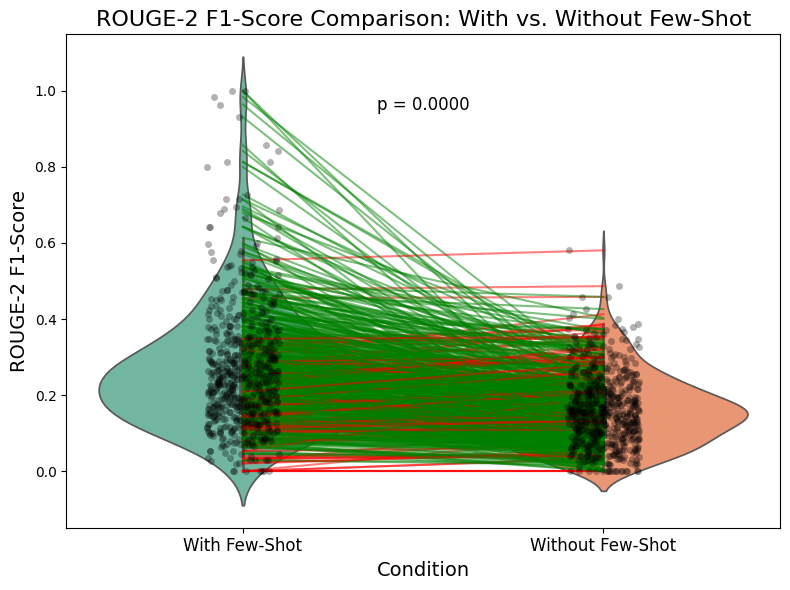
**

**Supplemental Figure 35. GPT-4o ROUGE-L F1 scores with and without few-shot examples – temperature 1, top-k=10.** The lines indicate the change for each impression, where green lines indicate an increase in the score with few-shot examples, and the red lines indicate decrease.

**
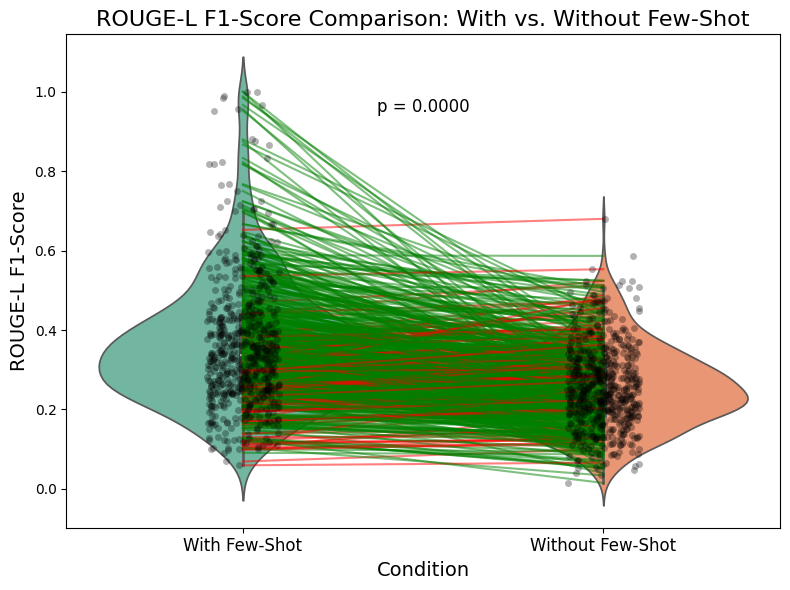
**

**Supplemental Figure 36. GPT-4o BERTScore F1 scores with and without few-shot examples – temperature 1, top-k=10.** The lines indicate the change for each impression, where green lines indicate an increase in the score with few-shot examples, and the red lines indicate decrease.

**
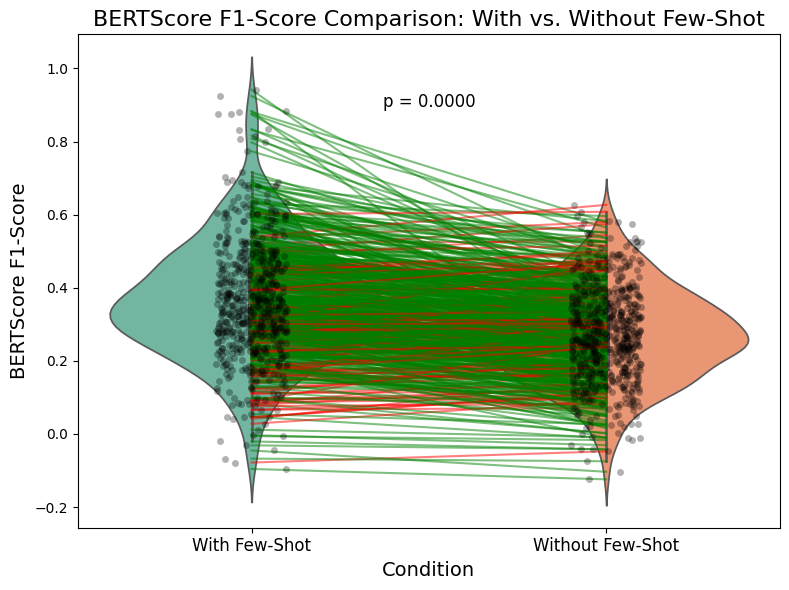
**

**Supplemental Figure 37. GPT-4o BERTScore Precision scores with and without few-shot examples – temperature 1, top-k=10.** The lines indicate the change for each impression, where green lines indicate an increase in the score with few-shot examples, and the red lines indicate decrease.


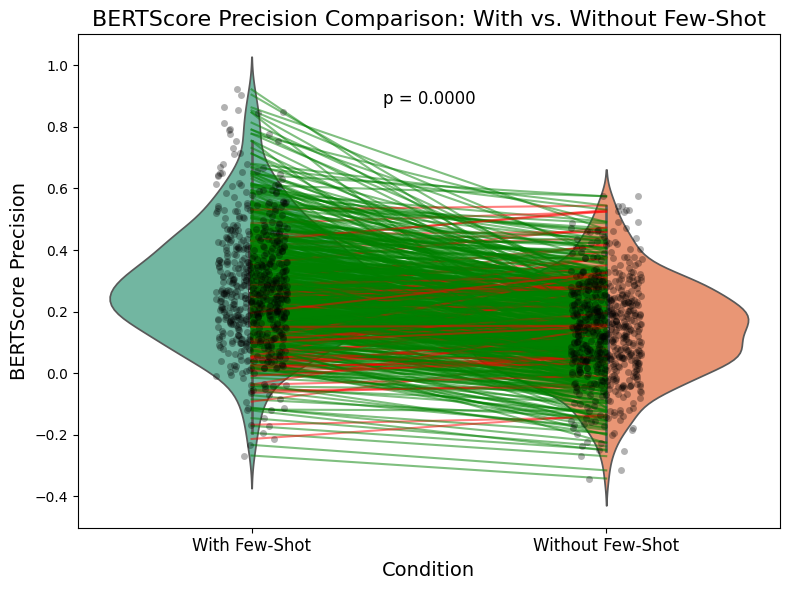


**Supplemental Figure 38. GPT-4o BERTScore Recall scores with and without few-shot examples – temperature 1, top-k=10.** The lines indicate the change for each impression, where green lines indicate an increase in the score with few-shot examples, and the red lines indicate decrease.


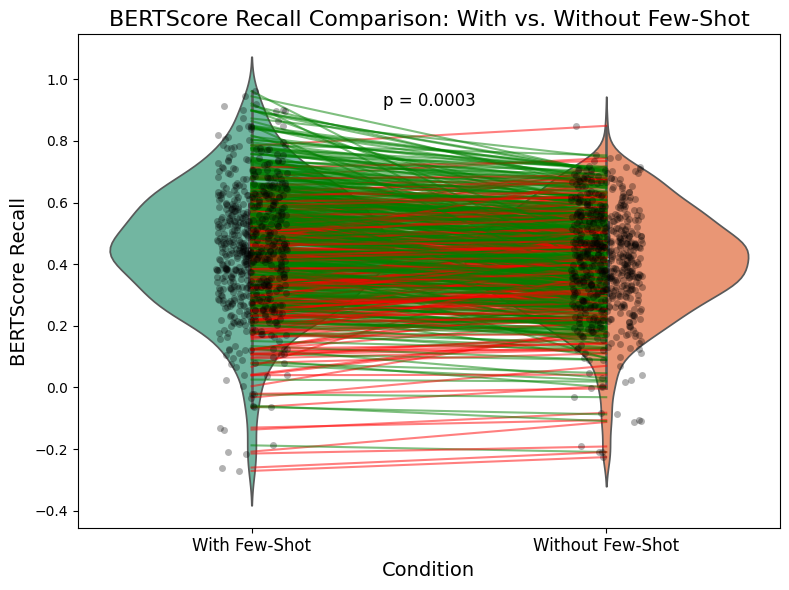


**Supplemental Figure 39. LLaMA 3.1-70B ROUGE-1 F1 scores with and without few-shot examples – temperature 0, top-k=3.** The lines indicate the change for each impression, where green lines indicate an increase in the score with few-shot examples, and the red lines indicate decrease.


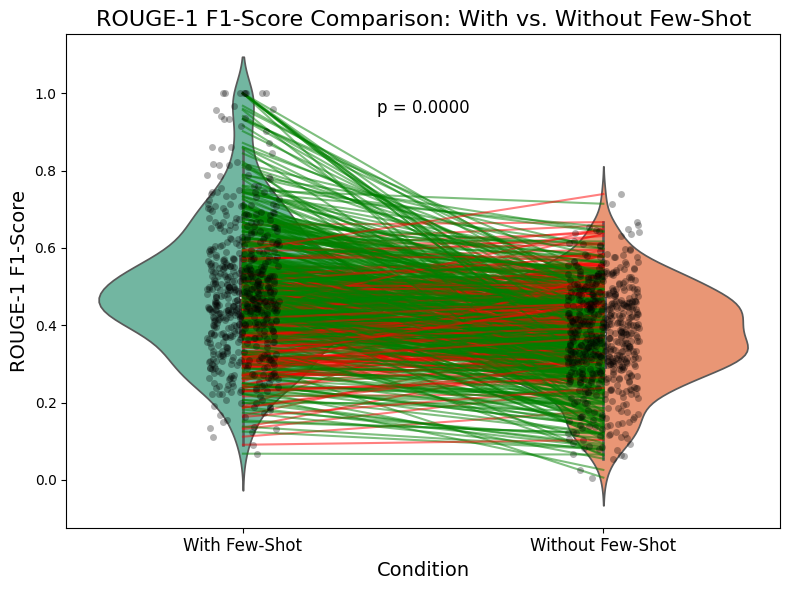


**Supplemental Figure 40. LLaMA 3.1-70B ROUGE-2 F1 scores with and without few-shot examples – temperature 0, top-k=3.** The lines indicate the change for each impression, where green lines indicate an increase in the score with few-shot examples, and the red lines indicate decrease.


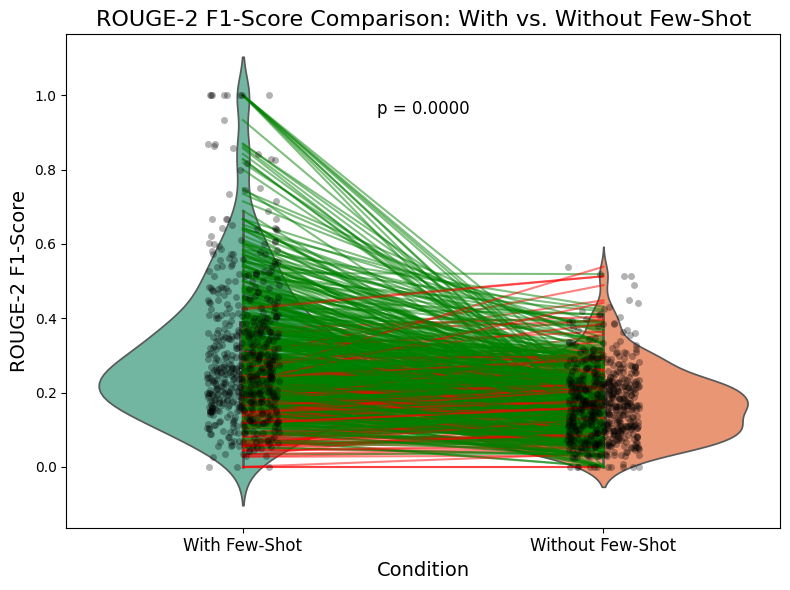


**Supplemental Figure 41. LLaMA 3.1-70B ROUGE-L F1 scores with and without few-shot examples – temperature 0, top-k=3.** The lines indicate the change for each impression, where green lines indicate an increase in the score with few-shot examples, and the red lines indicate decrease.


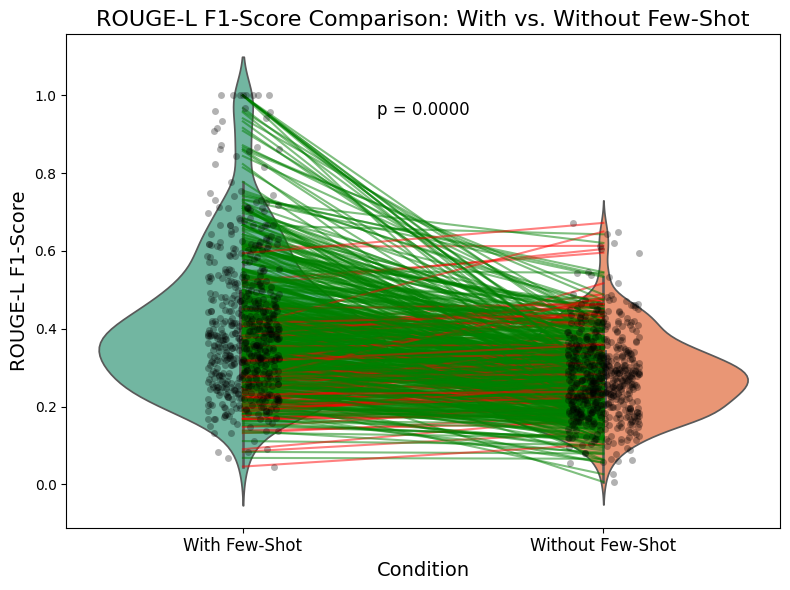


**Supplemental Figure 42. LLaMA 3.1-70B BERTScore F1 scores with and without few-shot examples – temperature 0, top-k=3.** The lines indicate the change for each impression, where green lines indicate an increase in the score with few-shot examples, and the red lines indicate decrease.


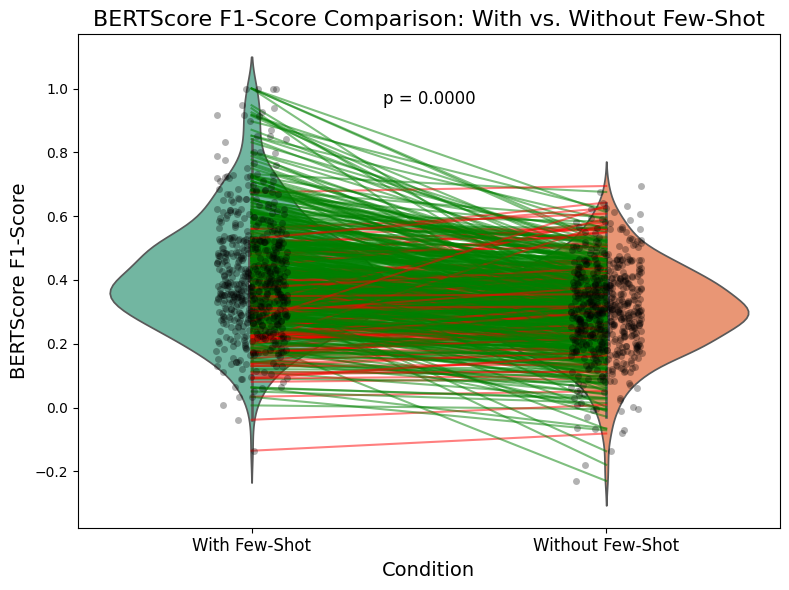


**Supplemental Figure 43. LLaMA 3.1-70B BERTScore precision scores with and without few-shot examples – temperature 0, top-k=3.** The lines indicate the change for each impression, where green lines indicate an increase in the score with few-shot examples, and the red lines indicate decrease.

**
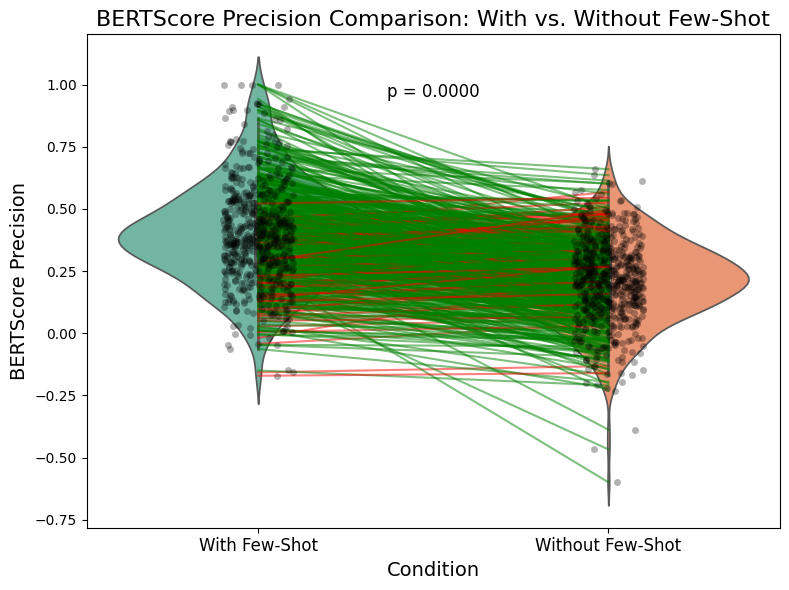
**

**Supplemental Figure 44. LLaMA 3.1-70B BERTScore recall scores with and without few-shot examples – temperature 0, top-k=3.** The lines indicate the change for each impression, where green lines indicate an increase in the score with few-shot examples, and the red lines indicate decrease.


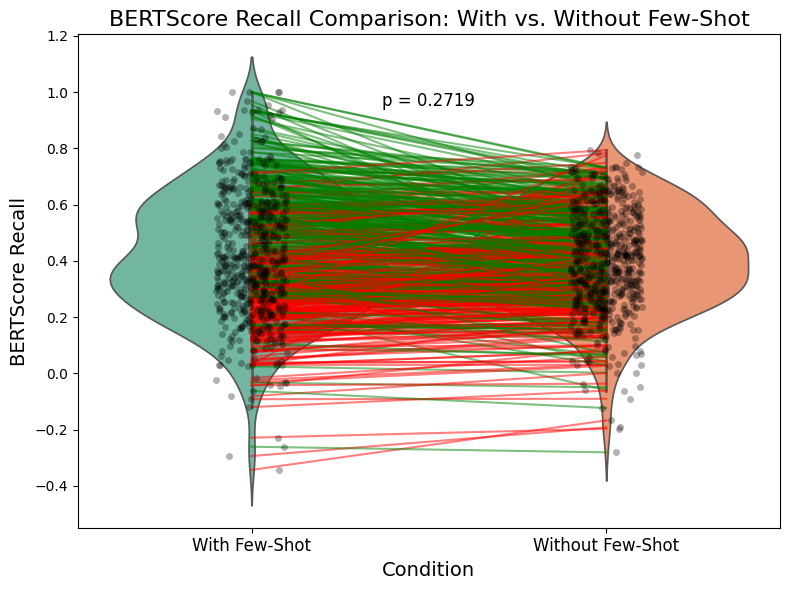


**Supplemental Figure 45. LLaMA 3.1-70B ROUGE-1 F1 scores with and without few-shot examples – temperature 0.7, top-k=3.** The lines indicate the change for each impression, where green lines indicate an increase in the score with few-shot examples, and the red lines indicate decrease.


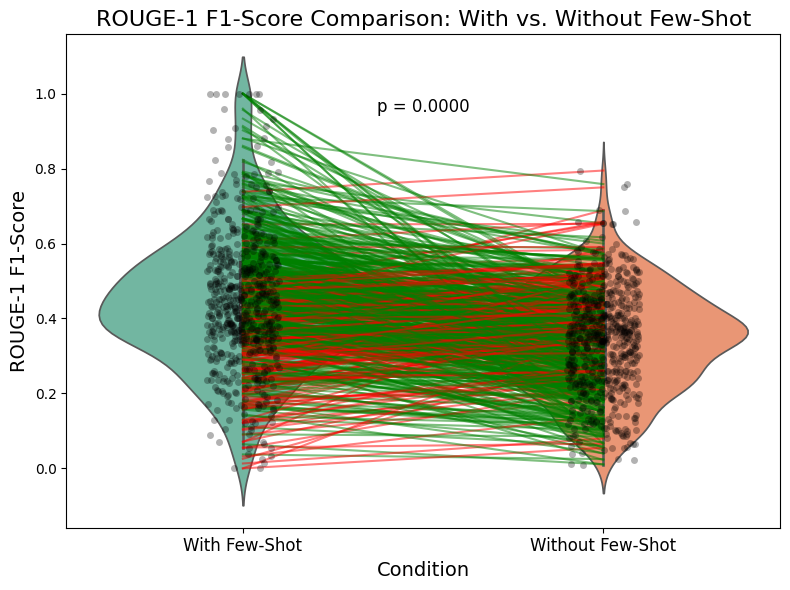


**Supplemental Figure 46. LLaMA 3.1-70B ROUGE-2 F1 scores with and without few-shot examples – temperature 0.7, top-k=3.** The lines indicate the change for each impression, where green lines indicate an increase in the score with few-shot examples, and the red lines indicate decrease.


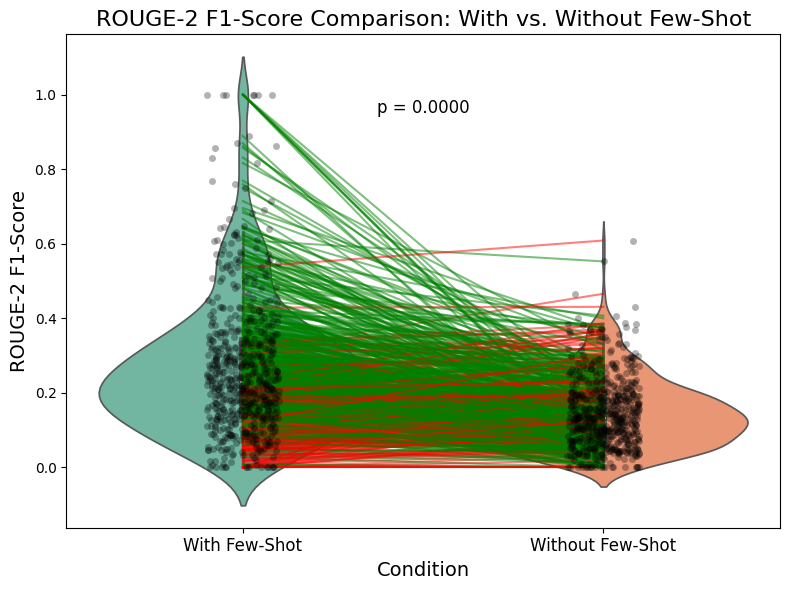


**Supplemental Figure 47. LLaMA 3.1-70B ROUGE-L F1 scores with and without few-shot examples – temperature 0.7, top-k=3.** The lines indicate the change for each impression, where green lines indicate an increase in the score with few-shot examples, and the red lines indicate decrease.


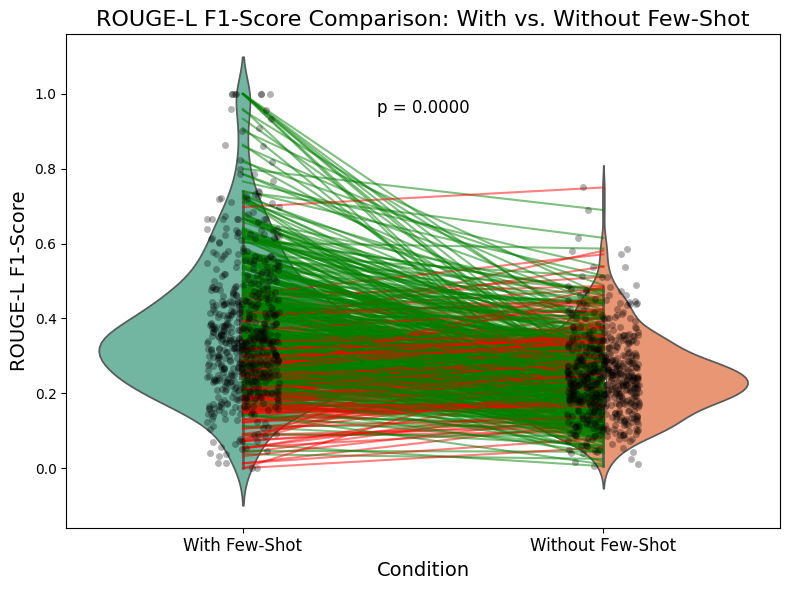


**Supplemental Figure 48. LLaMA 3.1-70B BERTScore F1 scores with and without few-shot examples – temperature 0.7, top-k=3.** The lines indicate the change for each impression, where green lines indicate an increase in the score with few-shot examples, and the red lines indicate decrease.


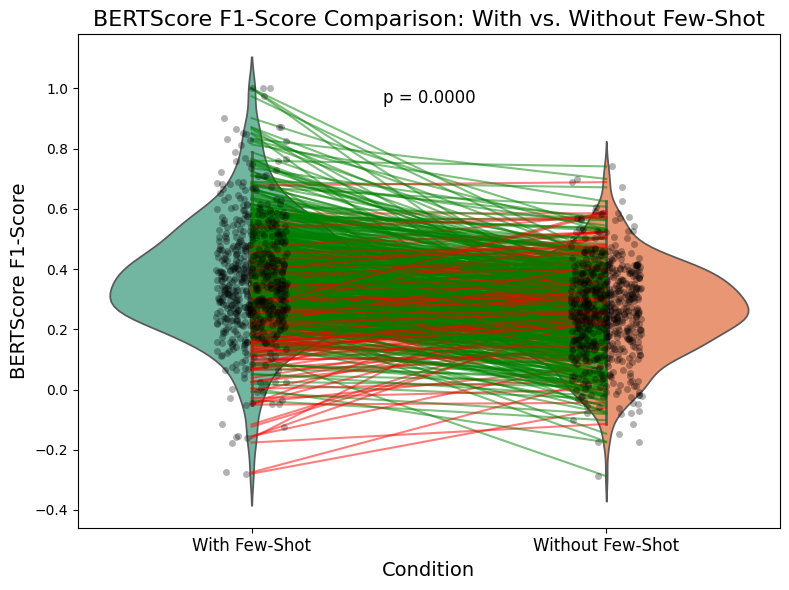


**Supplemental Figure 49. LLaMA 3.1-70B BERTScore precision scores with and without few-shot examples – temperature 0.7, top-k=3.** The lines indicate the change for each impression, where green lines indicate an increase in the score with few-shot examples, and the red lines indicate decrease.


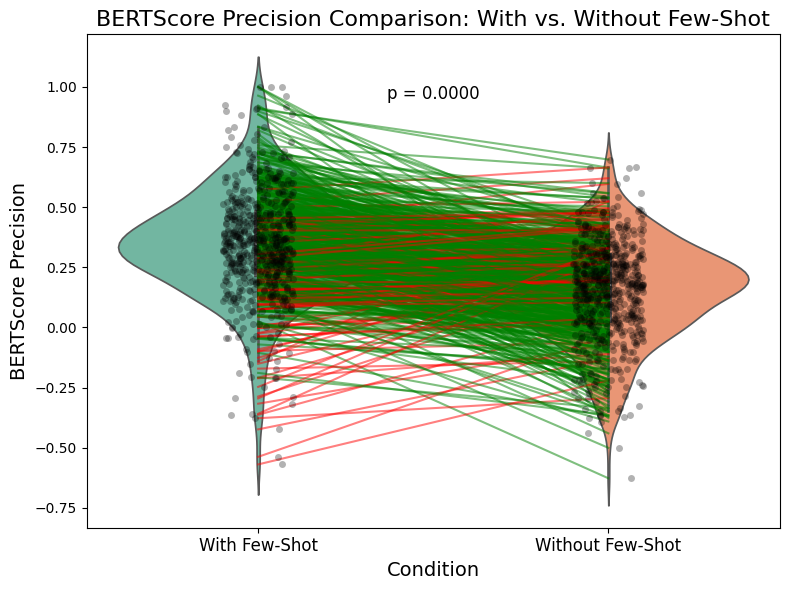


**Supplemental Figure 50. LLaMA 3.1-70B BERTScore recall scores with and without few-shot examples – temperature 0.7, top-k=3.** The lines indicate the change for each impression, where green lines indicate an increase in the score with few-shot examples, and the red lines indicate decrease.


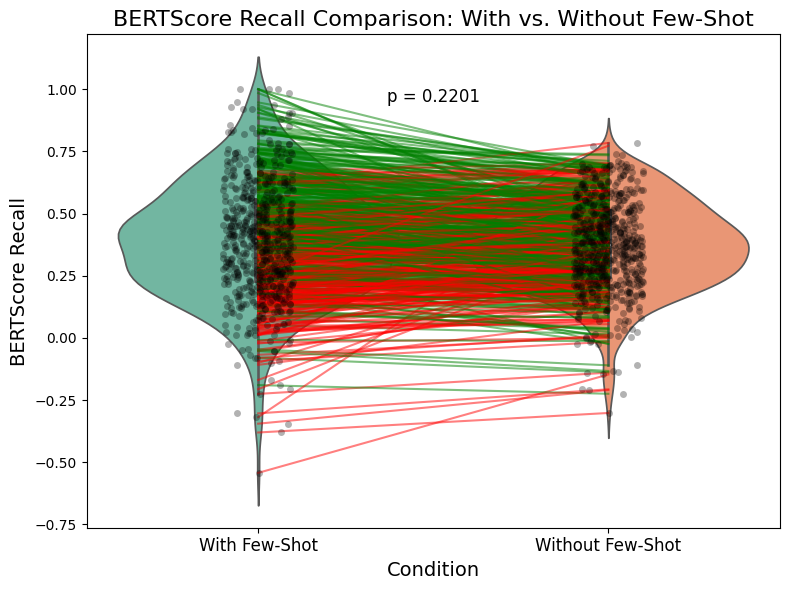


**Supplemental Figure 51. LLaMA 3.1-70B ROUGE-1 F1 scores with and without few-shot examples – temperature 1, top-k=3.** The lines indicate the change for each impression, where green lines indicate an increase in the score with few-shot examples, and the red lines indicate decrease.

**
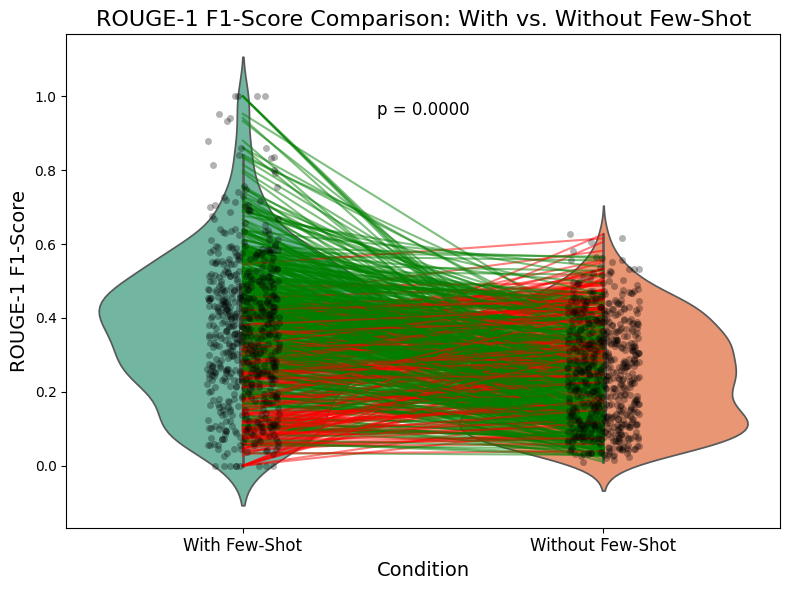
**

**Supplemental Figure 52. LLaMA 3.1-70B ROUGE-2 F1 scores with and without few-shot examples – temperature 1, top-k=3.** The lines indicate the change for each impression, where green lines indicate an increase in the score with few-shot examples, and the red lines indicate decrease.


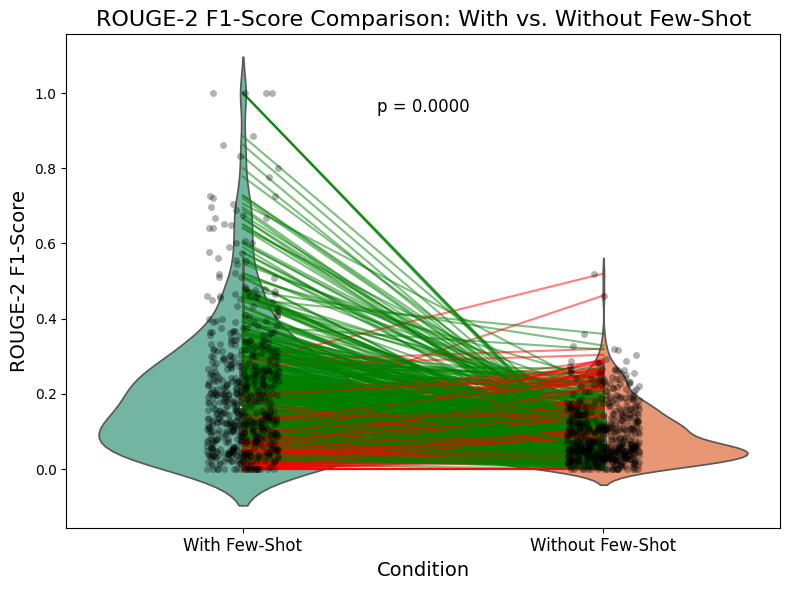


**Supplemental Figure 53. LLaMA 3.1-70B ROUGE-L F1 scores with and without few-shot examples – temperature 1, top-k=3.** The lines indicate the change for each impression, where green lines indicate an increase in the score with few-shot examples, and the red lines indicate decrease.


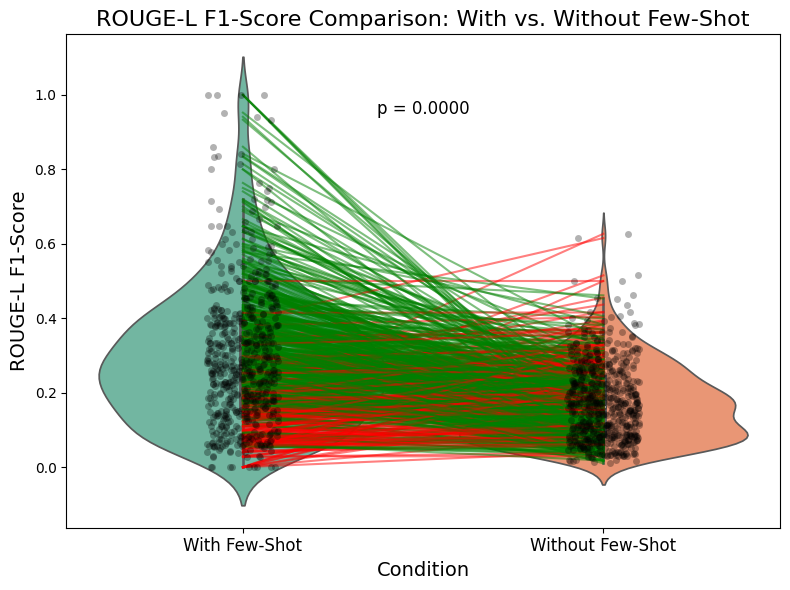


**Supplemental Figure 54. LLaMA 3.1-70B BERTScore F1 scores with and without few-shot examples – temperature 1, top-k=3.** The lines indicate the change for each impression, where green lines indicate an increase in the score with few-shot examples, and the red lines indicate decrease.


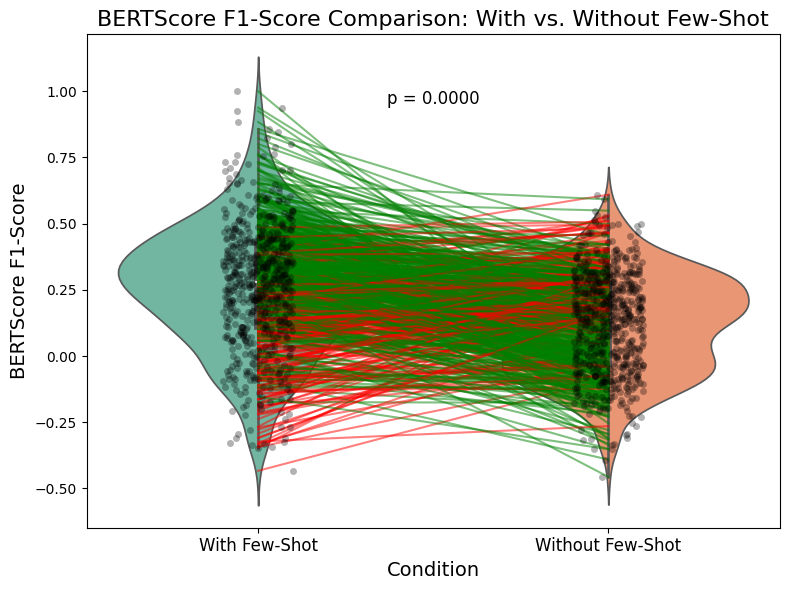


**Supplemental Figure 55. LLaMA 3.1-70B BERTScore precision scores with and without few-shot examples – temperature 1, top-k=3.** The lines indicate the change for each impression, where green lines indicate an increase in the score with few-shot examples, and the red lines indicate decrease.


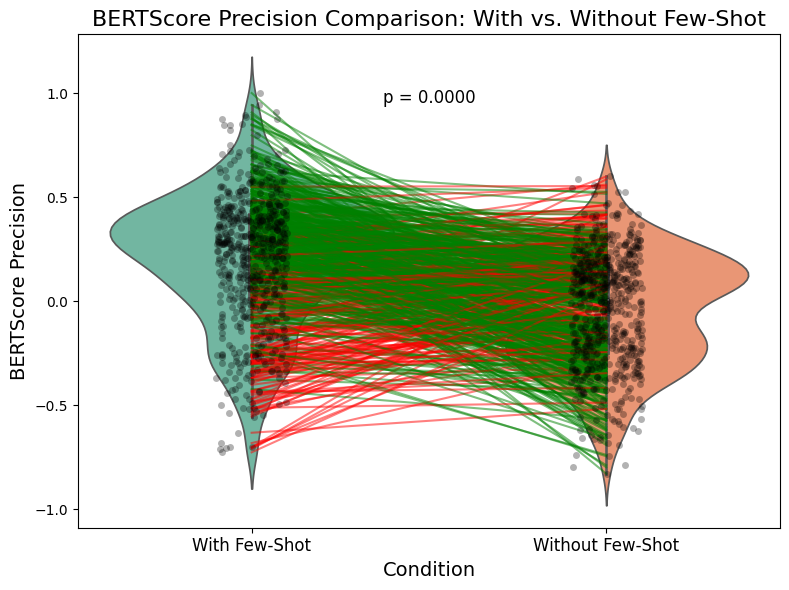


**Supplemental Figure 56. LLaMA 3.1-70B BERTScore recall scores with and without few-shot examples – temperature 1, top-k=3.** The lines indicate the change for each impression, where green lines indicate an increase in the score with few-shot examples, and the red lines indicate decrease.


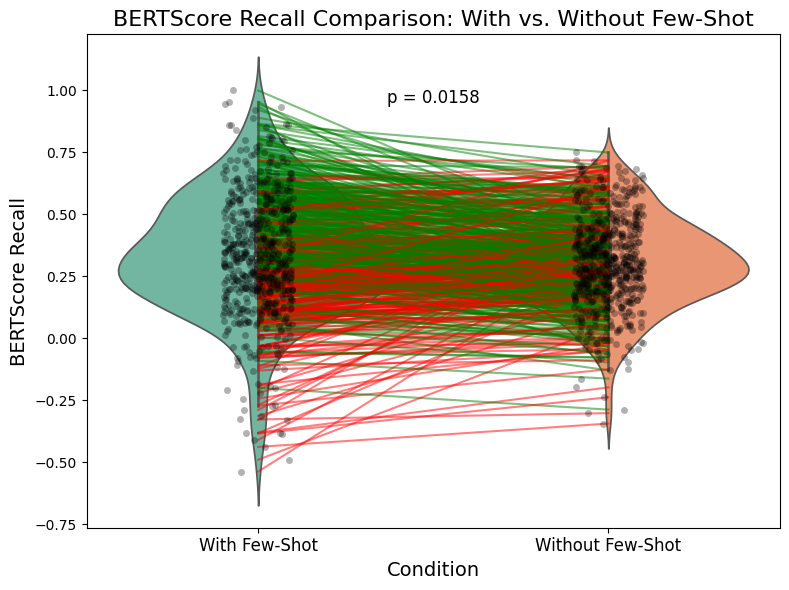


**Supplemental Figure 57. LLaMA 3.1-70B ROUGE-1 F1 scores with and without few-shot examples – temperature 0, top-k=10.** The lines indicate the change for each impression, where green lines indicate an increase in the score with few-shot examples, and the red lines indicate decrease.


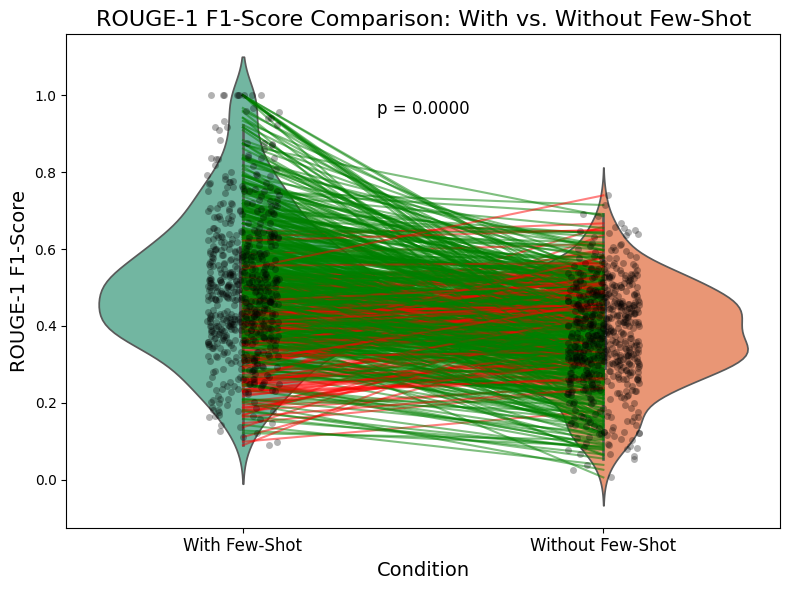


**Supplemental Figure 58. LLaMA 3.1-70B ROUGE-2 F1 scores with and without few-shot examples – temperature 0, top-k=10.** The lines indicate the change for each impression, where green lines indicate an increase in the score with few-shot examples, and the red lines indicate decrease.


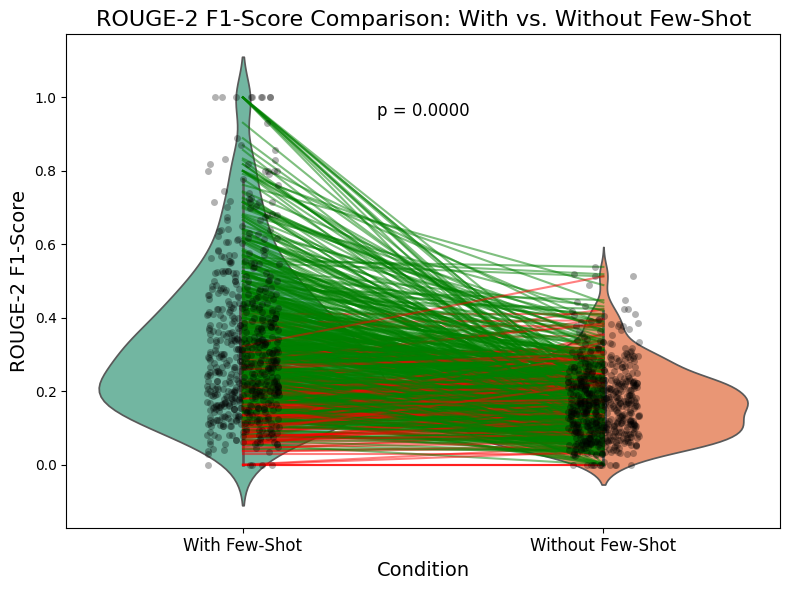


**Supplemental Figure 59. LLaMA 3.1-70B ROUGE-L F1 scores with and without few-shot examples – temperature 0, top-k=10.** The lines indicate the change for each impression, where green lines indicate an increase in the score with few-shot examples, and the red lines indicate decrease.

**
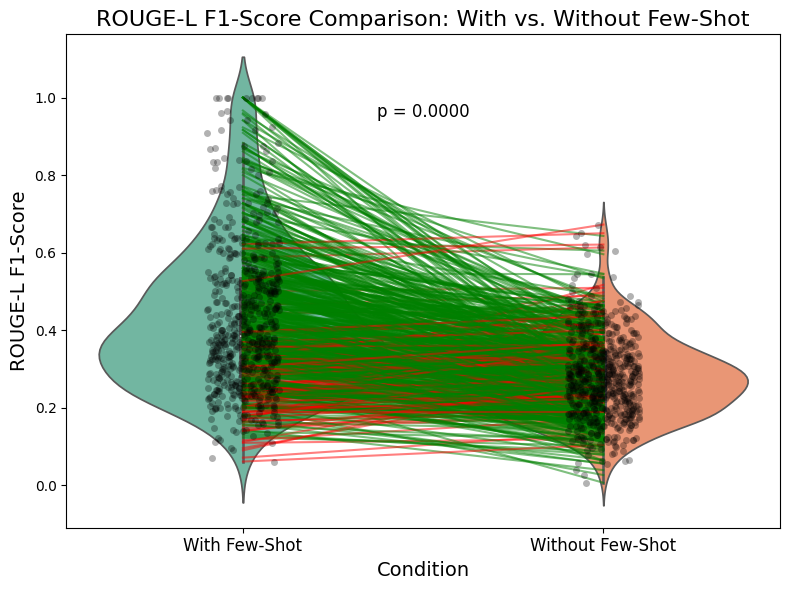
**

**Supplemental Figure 60. LLaMA 3.1-70B BERTScore F1 scores with and without few-shot examples – temperature 0, top-k=10.** The lines indicate the change for each impression, where green lines indicate an increase in the score with few-shot examples, and the red lines indicate decrease.


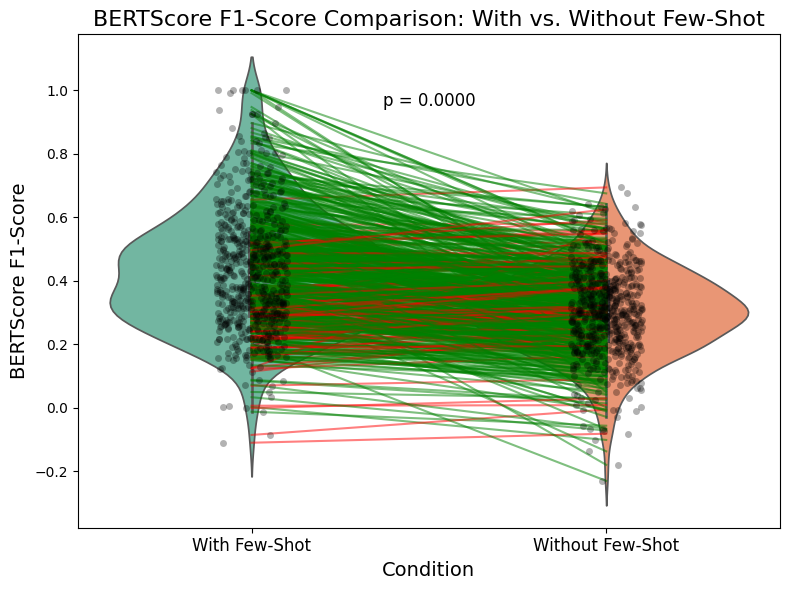


**Supplemental Figure 61. LLaMA 3.1-70B BERTScore precision scores with and without few-shot examples – temperature 0, top-k=10.** The lines indicate the change for each impression, where green lines indicate an increase in the score with few-shot examples, and the red lines indicate decrease.


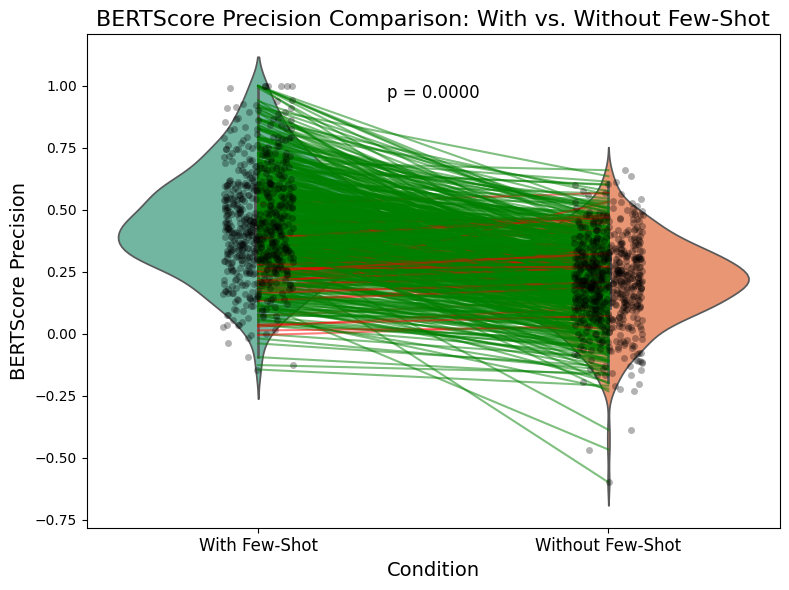


**Supplemental Figure 62. LLaMA 3.1-70B BERTScore recall scores with and without few-shot examples – temperature 0, top-k=10.** The lines indicate the change for each impression, where green lines indicate an increase in the score with few-shot examples, and the red lines indicate decrease.


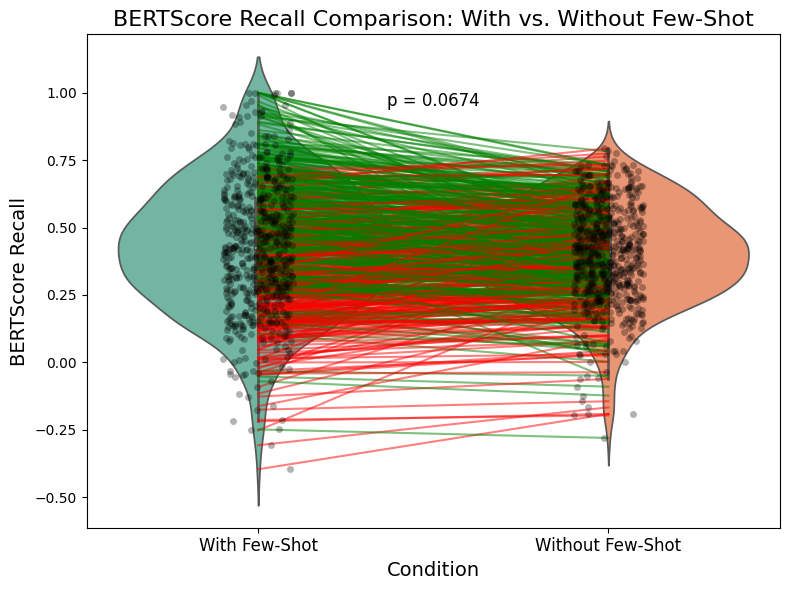


**Supplemental Figure 63. LLaMA 3.1-70B ROUGE-1 F1 scores with and without few-shot examples – temperature 0.7, top-k=10.** The lines indicate the change for each impression, where green lines indicate an increase in the score with few-shot examples, and the red lines indicate decrease.


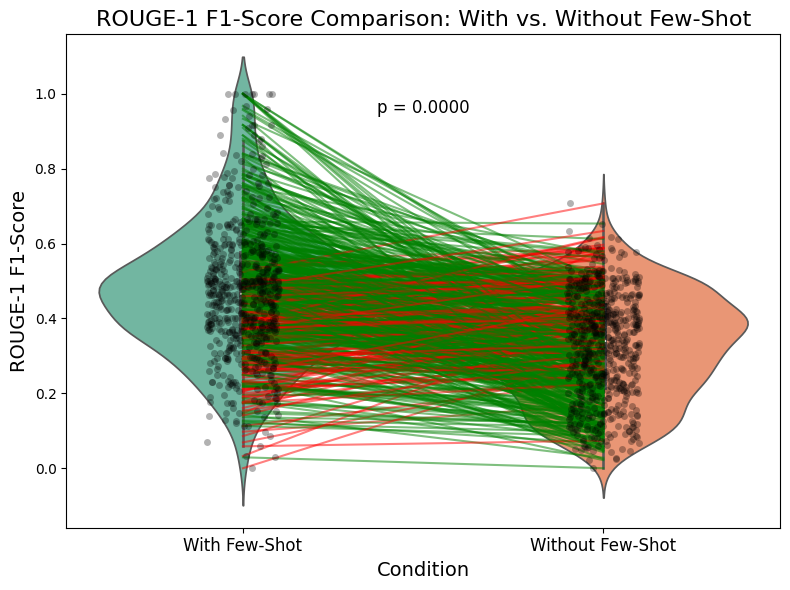


**Supplemental Figure 64. LLaMA 3.1-70B ROUGE-2 F1 scores with and without few-shot examples – temperature 0.7, top-k=10.** The lines indicate the change for each impression, where green lines indicate an increase in the score with few-shot examples, and the red lines indicate decrease.


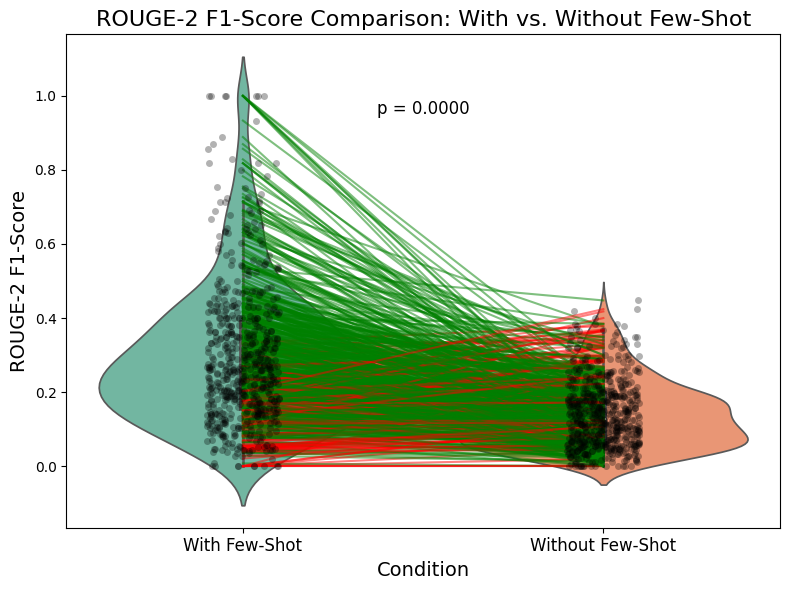


**Supplemental Figure 65. LLaMA 3.1-70B ROUGE-L F1 scores with and without few-shot examples – temperature 0.7, top-k=10.** The lines indicate the change for each impression, where green lines indicate an increase in the score with few-shot examples, and the red lines indicate decrease.


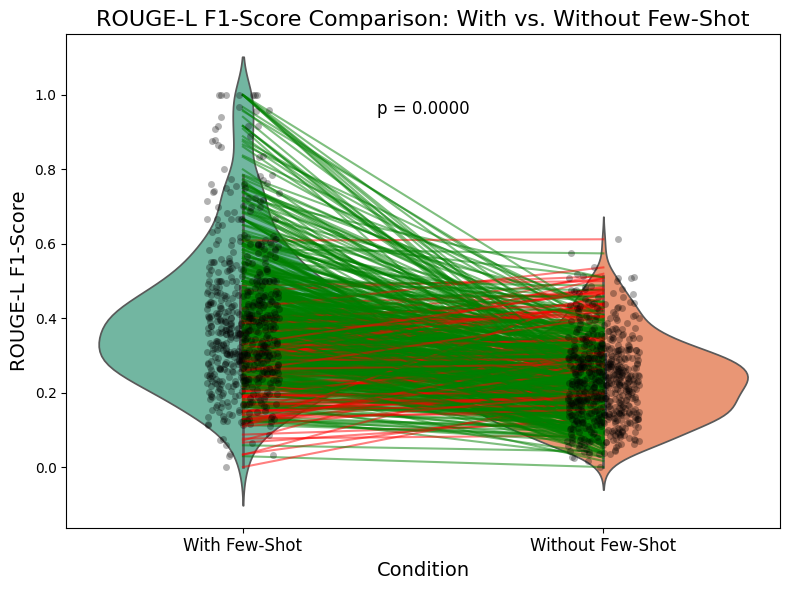


**Supplemental Figure 66. LLaMA 3.1-70B BERTScore F1 scores with and without few-shot examples – temperature 0.7, top-k=10.** The lines indicate the change for each impression, where green lines indicate an increase in the score with few-shot examples, and the red lines indicate decrease.


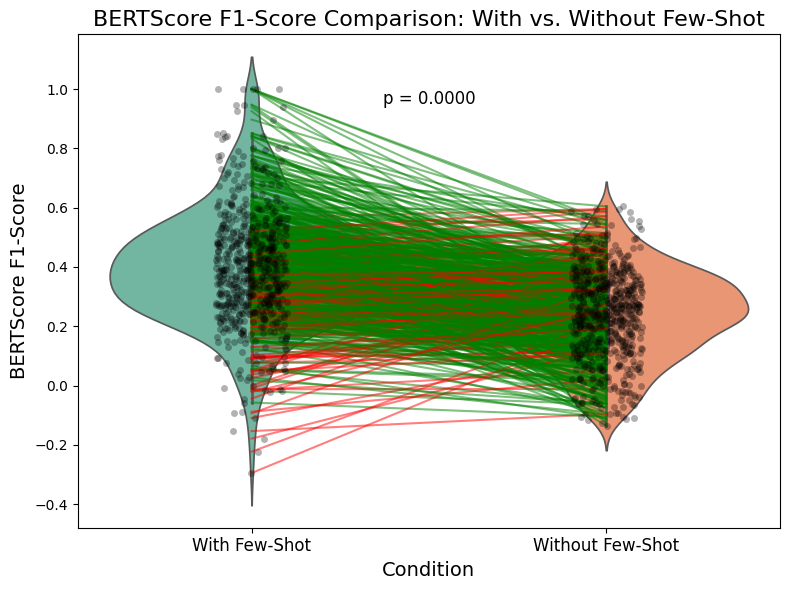


**Supplemental Figure 67. LLaMA 3.1-70B BERTScore precision scores with and without few-shot examples – temperature 0.7, top-k=10.** The lines indicate the change for each impression, where green lines indicate an increase in the score with few-shot examples, and the red lines indicate decrease.


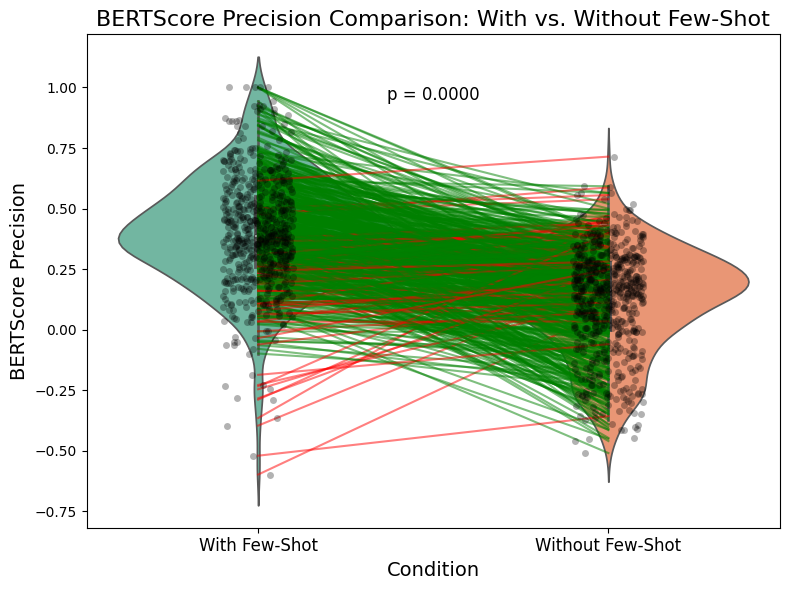


**Supplemental Figure 68. LLaMA 3.1-70B BERTScore recall scores with and without few-shot examples – temperature 0.7, top-k=10.** The lines indicate the change for each impression, where green lines indicate an increase in the score with few-shot examples, and the red lines indicate decrease.


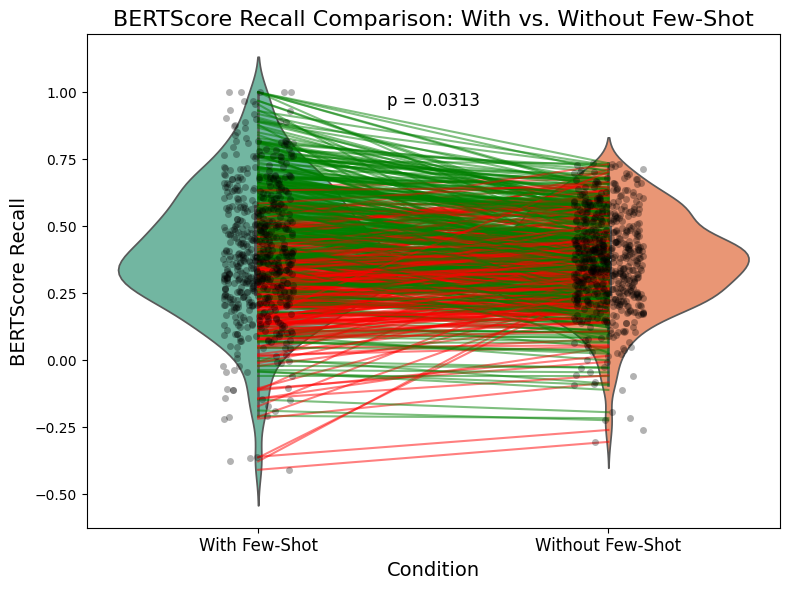


**Supplemental Figure 69. LLaMA 3.1-70B ROUGE-1 F1 scores with and without few-shot examples – temperature 1, top-k=10.** The lines indicate the change for each impression, where green lines indicate an increase in the score with few-shot examples, and the red lines indicate decrease.


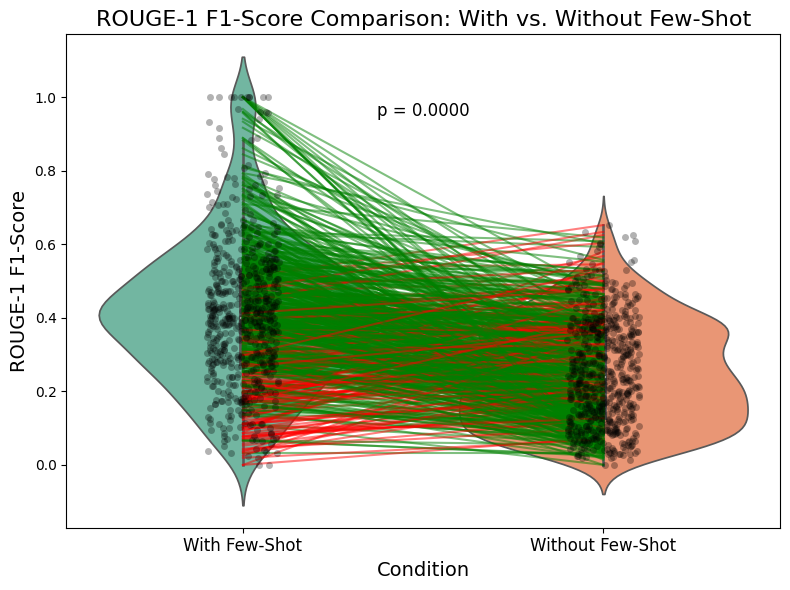


**Supplemental Figure 70. LLaMA 3.1-70B ROUGE-2 F1 scores with and without few-shot examples – temperature 1, top-k=10.** The lines indicate the change for each impression, where green lines indicate an increase in the score with few-shot examples, and the red lines indicate decrease.


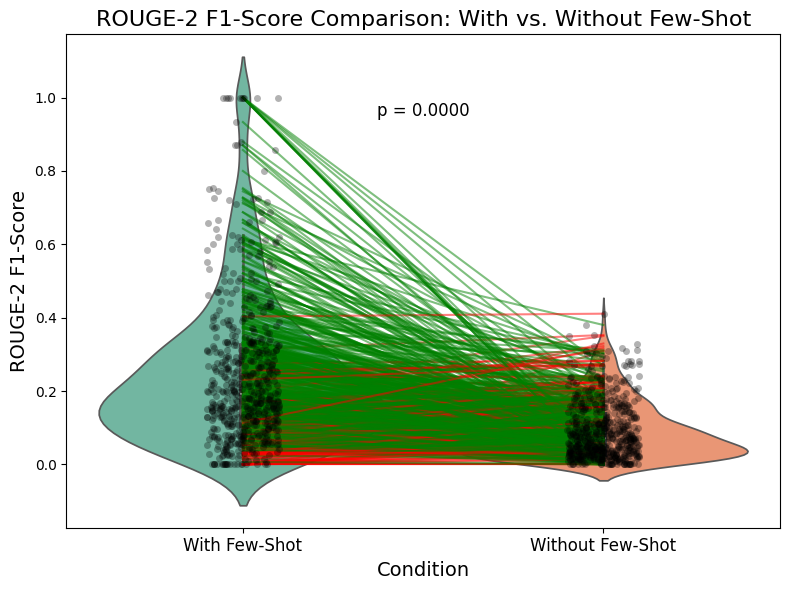


**Supplemental Figure 71. LLaMA 3.1-70B ROUGE-L F1 scores with and without few-shot examples – temperature 1, top-k=10.** The lines indicate the change for each impression, where green lines indicate an increase in the score with few-shot examples, and the red lines indicate decrease.


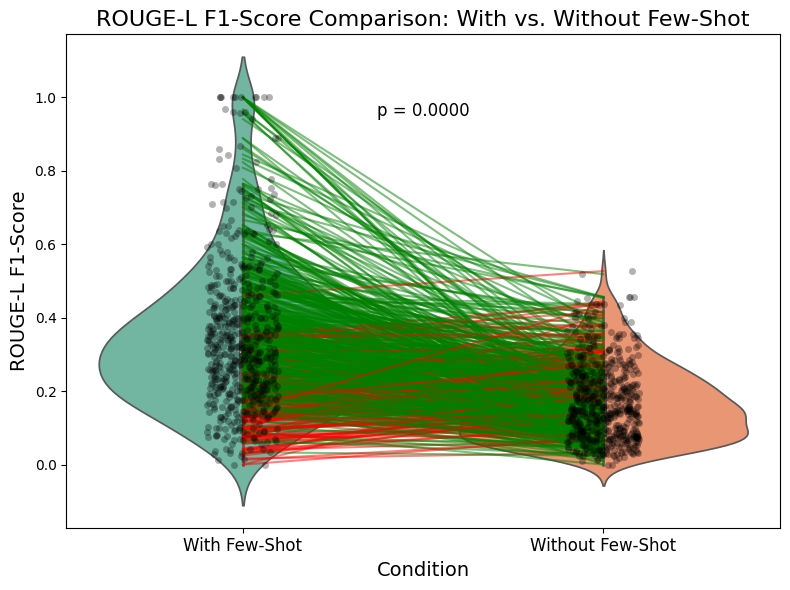


**Supplemental Figure 72. LLaMA 3.1-70B BERTScore F1 scores with and without few-shot examples – temperature 1, top-k=10.** The lines indicate the change for each impression, where green lines indicate an increase in the score with few-shot examples, and the red lines indicate decrease.


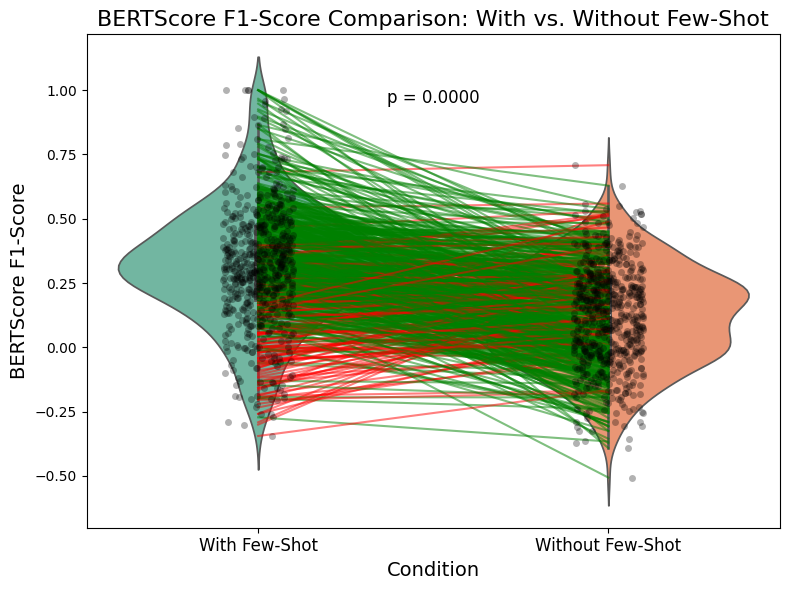


**Supplemental Figure 73. LLaMA 3.1-70B BERTScore precision scores with and without few-shot examples – temperature 1, top-k=10.** The lines indicate the change for each impression, where green lines indicate an increase in the score with few-shot examples, and the red lines indicate decrease.


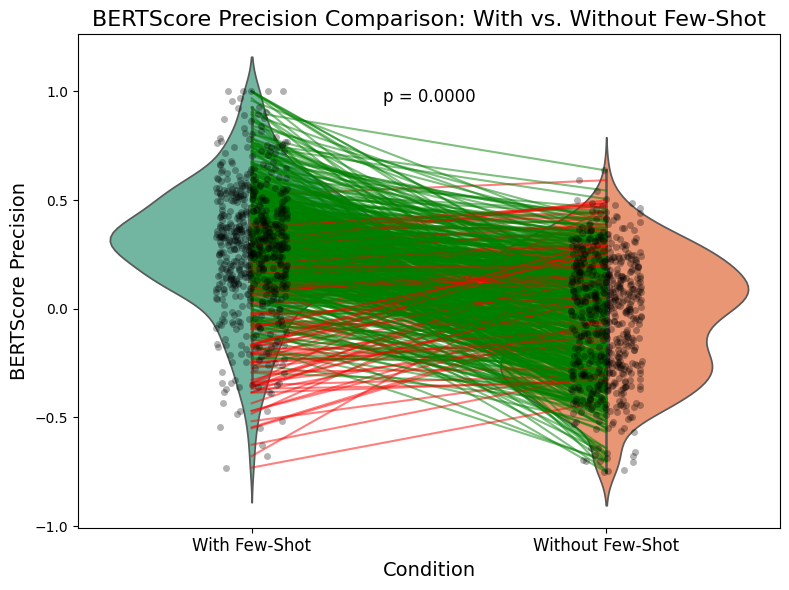


**Supplemental Figure 74. LLaMA 3.1-70B BERTScore recall scores with and without few-shot examples – temperature 1, top-k=10.** The lines indicate the change for each impression, where green lines indicate an increase in the score with few-shot examples, and the red lines indicate decrease.


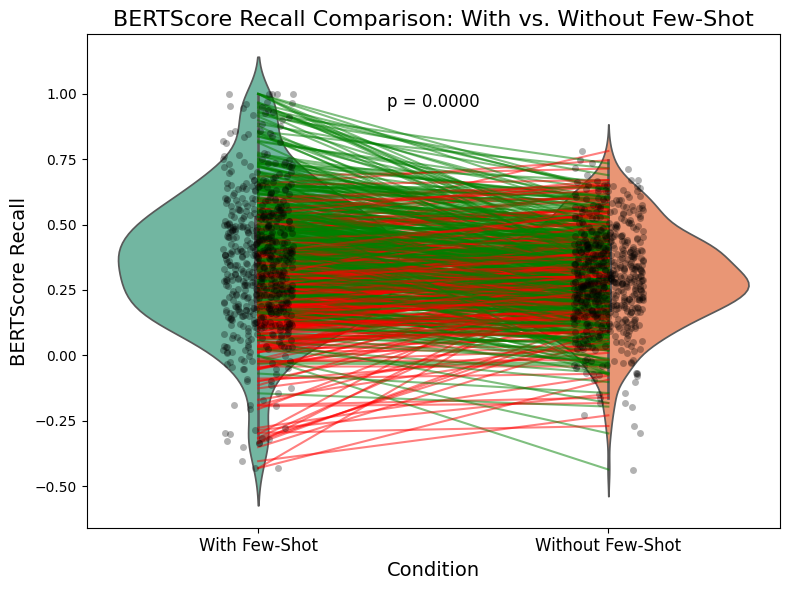


**Supplemental Prompt**

Read the following radiology findings and generate a clear, concise, and actionable impression based on the guidelines.

Guidelines:

{guidelines}

"
 if include_few_shot and few_shot_examples:
 final_prompt += f"Few-Shot Examples:\n\n{few_shot_examples}\n"


 final_prompt += (f"Input :\n{narrative}\n\nOutput Impression:\n")

Guidelines: |
 Lead with the Diagnosis:

 If a clinical question is specified, address it directly as the first numbered point.
 Begin with the most likely diagnosis or a prioritized differential diagnosis.
 Use specific disease names, health conditions, or radiologic signs when possible.
 Include only key findings that directly affect patient management.

 Use Clear and Accessible Language:
 Employ medical terminology understandable to a general medical audience.
 Avoid unnecessary technical jargon specific to radiology.

 Organize the Impression as a Numbered List.

 Ensure Statements are Supported by Findings:
 All statements must be directly supported by the findings.
 Do not restate findings that are not critical to the impression.

 Exclude Insignificant Information:
 Do not include benign or incidental findings that do not require action.
 Omit stable findings unless they are currently clinically important.
 Exclude any information that does not impact patient care or management.

 Remember:
 Be Extremely Concise: Include only the most critical and actionable information.
 Write for Referring Clinicians: Use language that is accessible and useful to them.
 Avoid Repetition: Do not repeat information or include unnecessary details.
 Formatting: Do not include any markdown formatting, code fences, or language identifiers.
