# Supplementary material for: Case-matched retrieval improves textual alignment of LLM-generated radiology impressions
Source: PLoS One. 2026 Jul 31;21(7):e0354688. doi: 10.1371/journal.pone.0354688 (PMC13426976; doi:10.1371/journal.pone.0354688)
Supplement: Attachment — Submitted filename: response_letter.docx [file pone.0354688.s003.docx]

04/12/2026

Response to Review: PONE-D-25-66963

Dear Dr. Krit Pongpirul,

We would like to thank you for the opportunity to submit a revised version of our manuscript titled “Case-Matched Retrieval Improves Textual Alignment of LLM-Generated Radiology Impressions”. We would also like to thank the editor and the reviewers for their valuable comments.

Below are our responses to the editor’s and the reviewers’ comments, and the revised manuscript is attached.

Please do not hesitate to contact us regarding any further issue or question.

**Editor’s Comments to the Authors:**

**Editor’s comment:**

The manuscript addresses an important and timely problem in medical informatics—namely, reducing the administrative burden on radiologists through inference-time strategies such as retrieval-augmented generation (RAG). The study benefits from a large, real-world dataset of nearly 12,000 CT pulmonary angiography reports, a pragmatic focus on inference-time adaptation rather than model retraining, and evaluation across both proprietary and open-source large language models. These elements place the work squarely within the scope of PLOS ONE.

However, the reviewer raises several methodological and reporting concerns that affect the interpretation and strength of the conclusions. In particular, the following points require attention:

**Response:**

Thank you, we appreciate the comment. We revised the manuscript throughout.

**Editor’s comment:**

The current manuscript overstates its findings by implying improvement in clinical quality or diagnostic validity of radiology impressions. The reported results demonstrate improved textual or stylistic alignment with reference impressions using automated NLP metrics. Please revise the language throughout the manuscript (including title, abstract, results, and conclusions) to clearly and consistently reflect this distinction and to avoid claims of clinical accuracy, safety, or effectiveness.

**Response:**

Thank you, we agree. We revised the manuscript throughout to better distinguish textual alignment with reference impressions from clinical accuracy or diagnostic validity. We now describe the main finding as improved performance on automated text-similarity metrics and explicitly note that these metrics do not establish clinical correctness and that radiologist review remains necessary.

Revised title:

*“Case-Matched Retrieval Improves Textual Alignment of LLM-Generated Radiology Impressions”*

In the Abstract:

*“Results*

*Dynamic retrieval-based few-shot prompting outperformed zero-shot and fixed few-shot prompting across all configurations (all p<0.05). The highest scores were observed at temperature 0 and k=10. ROUGE-1 F1 increased to 0.44–0.47 for GPT-4o and 0.37–0.50 for LLaMA, versus 0.35–0.37 and 0.25–0.37, respectively, in zero-shot prompting. Lower temperature and larger k were associated with higher similarity scores.*

*Conclusions*

*Dynamic, case-matched retrieval improved alignment of LLM-generated CTPA impressions with reference impressions on automated text-similarity metrics. Scores remained moderate, and radiologists’ verification is still required before clinical deployment.”*

In the Discussion:

*“This study has several limitations. It was retrospective, limited to a single exam type (CTPA), and evaluated only two LLMs. In addition, we did not perform human evaluation of the generated impressions, so we cannot determine whether higher similarity scores corresponded to better clinical quality, correctness, or safety. Evaluation relied exclusively on automated similarity metrics. ROUGE and BERTScore are useful for benchmarking textual overlap and semantic similarity to reference impressions, but they are not measures of diagnostic accuracy or clinical correctness. They do not reliably detect negation errors, clinically important omissions, unsupported statements, or harmful hallucinations. Accordingly, the improvements reported here should be interpreted as gains in textual alignment with reference impressions rather than evidence of clinical safety, diagnostic validity, or readiness for real-world deployment. We also did not perform a patient-level split analysis, which may affect independence between the retrieval bank and test set and should be addressed in future work.*

*In conclusion, dynamic, case-matched retrieval consistently improved alignment of LLM-generated CTPA impressions with reference impressions on automated text-similarity metrics. The largest gains were seen at temperature 0 and k=10. Further work with human evaluation will be needed to determine whether these improvements translate to clinically meaningful gains.”*

**Editor’s comment:**

The exclusive reliance on automated similarity metrics (ROUGE and BERTScore) must be more explicitly framed as a benchmarking approach rather than a proxy for clinical correctness. Please substantially strengthen the limitations section to clarify:
- That these metrics do not assess diagnostic accuracy, negation errors, omissions, or harmful hallucinations.
- That no claims are made regarding clinical safety or real-world deployment without human verification.

**Response:**

We completely agree and revised the limitation paragraph:

*“This study has several limitations. It was retrospective, limited to a single exam type (CTPA), and evaluated only two LLMs. In addition, we did not perform human evaluation of the generated impressions, so we cannot determine whether higher similarity scores corresponded to better clinical quality, correctness, or safety. Evaluation relied exclusively on automated similarity metrics. ROUGE and BERTScore are useful for benchmarking textual overlap and semantic similarity to reference impressions, but they are not measures of diagnostic accuracy or clinical correctness. They do not reliably detect negation errors, clinically important omissions, unsupported statements, or harmful hallucinations. Accordingly, the improvements reported here should be interpreted as gains in textual alignment with reference impressions rather than evidence of clinical safety, diagnostic validity, or readiness for real-world deployment. We also did not perform a patient-level split analysis, which may affect independence between the retrieval bank and test set and should be addressed in future work.”*

**Editor’s comment:**

The manuscript should include appropriate measures of precision. Please report 95% confidence intervals (or equivalent uncertainty estimates) for primary outcome metrics and clarify variability across test cases. Point estimates and p-values alone are insufficient for assessing the robustness of the observed differences.

**Response:**

Thank you. We added 95% confidence intervals for mean metric values across all supplemental performance tables and clarified this in the Methods section.

**Editor’s comment:**

To improve interpretability and address concerns about superficial syntactic overlap, please include an analysis of model failures. At minimum, we recommend a qualitative or semi-quantitative review of the lowest-performing subset of generated impressions (e.g., lowest decile by ROUGE-L), with categorization of error types (e.g., benign paraphrasing, omission, negation error, hallucinated findings).

**Response:**

We completely agree. we added a qualitative failure analysis of the lowest-performing generated impressions. Specifically, we reviewed the 50 lowest ROUGE-L F1 outputs for each model (overall 100 impressions) and categorized error types as wrong prioritization/redundant details, truncated or malformed output, hallucinated findings, overinterpretation/unsupported recommendations, omission, benign rephrasing, or other.

In Methods:

*“We also performed qualitative analysis on a subset of cases to assess whether low similarity scores reflected superficial wording differences or clinically important errors, the 50 lowest-scoring generated impressions by ROUGE-L F1 for each model (overall 100 impressions) were evaluated by a radiologist. Error categories included wrong prioritization/redundant details, truncated or malformed output, hallucinated findings, overinterpretation/unsupported recommendations, omission, benign rephrasing, and other.”*

In Results:

*“Review of the 50 lowest-scoring impressions per model showed different error profiles (Supplemental Tables 9–12). GPT-4o failures were dominated by wrong prioritization or redundant details (37/50, 74%), whereas LLaMA 3.1-70B failures were dominated by truncated or malformed output (34/50, 68%). Hallucinations were observed in both models (GPT-4o: 5/50, 10%; LLaMA 3.1-70B: 9/50, 18%), while omissions were less common (GPT-4o: 3/50, 6%; LLaMA 3.1-70B: 4/50, 8%). Representative examples are provided in Supplemental Table 12.”*

In the Supplement, we added the following tables: Supplemental Table 9 - Distribution of Lowest Scoring Impressions by Error Type per Model; Supplemental Table 10 - Distribution by Model and Strategy; Supplemental Table 11 - Distribution by Model and Top-k; Supplemental Table 12 - Representative low-scoring cases with assigned error category

**Editor’s comment:**

Please consider adding:
- A clear description or diagram of cohort inclusion/exclusion and data flow.
- Clarification regarding patient-level versus report-level splitting and its implications.
- A more explicit statement regarding computational and workflow considerations, noting that latency and operational feasibility were not evaluated in this study

**Response:**

Thank you for these helpful suggestions. We revised the manuscript accordingly.

We clarified cohort assembly and data flow in the Methods and Figure 1 legend. Specifically, we now state that 11,998 CTPA reports from 10,681 unique patients met study inclusion during the specified time window, and that these reports were randomly divided into a retrieval bank (n=11,399) and held-out test set (n=599). We also revised Figure 1 to better depict the study data flow from cohort identification to retrieval-bank construction, test-set selection, retrieval of top-k similar reports, and impression generation.

We clarified that the split was performed at the report level, not the patient level. We added text noting that multiple reports from the same patient could be included, which may reduce strict independence between retrieval and test data compared with a patient-level split. We now state this more explicitly in Methods and Limitations.

We added an explicit statement that this study focused on impression-generation quality under different retrieval configurations and did not evaluate latency, computational burden, throughput, implementation complexity, or real-world workflow feasibility, and that findings should be interpreted as benchmarking textual alignment rather than any indication for impact on clinical deployment.

**Reviewers’ Comments to Authors:**

**Reviewer 1:**

**Reviewer comment:**

This manuscript presents a technically sound and clearly scoped evaluation of case‑matched retrieval for improving LLM‑generated radiology impressions. The methods are appropriate and the analyses are transparent. Limitations have also been acknowledged.

**Response:**

Thank you, we appreciate the comment, and the time and effort in reviewing our manuscript.

**Reviewer 2:**

**Reviewer comment:**
This manuscript evaluates the effectiveness of Retrieval-Augmented Generation (RAG) in improving the quality of radiology impressions produced by LLMs. The authors analyze a retrospective cohort of 11,998 CTPA reports, comparing a dynamic, case-matched retrieval few-shot prompting strategy with zero-shot and fixed random few-shot baselines.

The clinical issue discussed in this manuscript is significant to contemporary healthcare; however, the research execution is severely flawed. The manuscript employs a validation strategy that depends on superficial syntactical mimicry instead of clinical accuracy, making the findings mathematically questionable and diagnostically ineffective. The authors replaced thorough human evaluation with automated n-gram overlap metrics, resulting in a simplified narrative that overlooks the complexities of real-world acute care workflows. The manuscript demonstrates technical deficiencies and does not comply with essential statistical reporting standards, particularly lacking 95% Confidence Intervals (CIs). The assertion that RAG "improves" impressions is an exaggeration, as the data only demonstrates increased syntactical overlap, not clinical accuracy or safety.

**Response:**

We thank the reviewer for the careful critique. We agree that automated text-similarity metrics do not establish clinical accuracy, safety, or readiness for acute-care deployment, and we have revised the manuscript to make this limitation more explicit throughout. Our intent was to evaluate textual alignment of generated impressions with reference radiologist impressions under different inference strategies, not to claim diagnostic correctness or clinical equivalence.

In response to this comment, we made several changes. We revised the title, abstract, results, discussion, and conclusions to consistently frame the findings as improved textual alignment rather than improved clinical quality or safety.

We added 95% confidence intervals for all mean metric values in all Supplementary Tables to address statistical reporting completeness.

We expanded the limitations and discussion sections to state explicitly that ROUGE and BERTScore are benchmarking tools for textual overlap and semantic similarity, not measures of diagnostic accuracy, factual correctness, or clinical safety. We also now state clearly that latency, throughput, compute cost, and workflow feasibility were not evaluated.

To address the concern that overlap-based metrics may reflect superficial syntactic mimicry, we also added a failure analysis of the lowest-performing subset of impressions. This qualitative/semi-quantitative review showed that low scores were associated with clinically relevant failure modes, including omission, hallucination, overinterpretation, wrong prioritization of details, and truncated or malformed output, rather than simple stylistic variation alone. These additions are intended to improve interpretability and to better delineate what the present study does and does not show.

**Reviewer comment:**

It is essential to identify the strengths and logical merits within the manuscript. The administrative load on radiologists is untenable. Exploring inference-time strategies such as RAG to automate the synthesis of findings into impressions addresses a significant bottleneck in real-world applications and is a legitimate focus of applied medical informatics. The use of a dataset with nearly 12,000 CTPA reports from over 10,000 unique patients is commendable. This offers a substantial and anatomically varied repository of pulmonary vascular pathology for the embedding database, significantly surpassing the limited volumes observed in many early pilot AI studies. The shift from expensive, resource-intensive fine-tuning methods (e.g., LoRA) to dynamic, inference-time RAG is pragmatic. The authors assert that RAG enables model adaptation to specific institutional reporting styles and updated clinical guidelines by updating the vector database, thus avoiding the significant overhead of continuous neural network retraining. This evaluation of a proprietary model (GPT-4o) and an open-source model (LLaMA 3.1-70B) facilitates a robust comparative analysis. This study examines the performance of open-source models, favored in healthcare for patient data privacy, compared to leading commercial APIs.

**Response:**

Thank you, we appreciate the comment.

**Reviewer comment:**

This study's methodological framework is based on critical assumptions. The authors assess the effectiveness of their RAG pipeline using automated natural language processing metrics: ROUGE and BERTScore F1. This dependence on automated metrics represents a superficial validation that obscures the actual clinical effectiveness of the algorithms.

In a CT Pulmonary Angiography report, these metrics are diagnostically inadequate and potentially negligent. A generative model could produce the impression: "There is no evidence of a central pulmonary embolism." If the original radiologist wrote,"There is evidence of a central pulmonary embolism," and the two sentences exhibit nearly identical lexical composition and contextual token clusters, differing solely by one negative modifier. ROUGE and BERTScore would assign a high similarity score to this generated text, overlooking the LLM's hallucination of a false negative that could lead to the patient being denied essential anticoagulation therapy. The ROUGE-1 F1 scores of 0.44 to 0.50 are considered successful; however, the authors do not perform any manual error analysis to identify the nature of the remaining 50% to 56% of the divergent text. Did the model merely employ a synonym (e.g., "atelectasis" for "collapse"), which is clinically benign, or did it fabricate a nonexistent lung nodule?

**Response:**

Thank you for this important point. We absolutely agree that automated text-similarity metrics alone are insufficient to establish clinical correctness or safety. ROUGE and BERTScore quantify textual overlap and semantic similarity to the reference impression, but they do not reliably detect clinically important negation errors, omissions, hallucinated findings, or unsupported recommendations. We have revised the manuscript to make this limitation more explicit and to avoid overstating these metrics as evidence of clinical validity.

In direct response to this concern, we added a qualitative error analysis of the lowest-performing generated impressions. Specifically, we reviewed the 50 lowest ROUGE-L cases for GPT-4o and the 50 lowest ROUGE-L cases for LLaMA 3.1-70B and categorized errors into clinically relevant groups, including wrong prioritization/redundant detail, truncated or malformed output, hallucinated findings, overinterpretation/unsupported recommendation, omission, and benign rephrasing. These results are now summarized in Supplemental Tables 9-12.

This additional review showed that low scores did not reflect a single failure mode. For GPT-4o, the dominant error pattern was wrong prioritization or inclusion of redundant details rather than catastrophic fabrication. For LLaMA 3.1-70B, a major failure mode was truncated or malformed output, with additional cases of hallucinated findings and omission.

We also revised the Discussion and Limitations sections to emphasize that the reported improvements should be interpreted as gains in textual alignment with reference impressions, not as evidence of clinical safety, diagnostic accuracy, or readiness for deployment. Human evaluation remains necessary to determine whether these outputs are clinically acceptable.

**Reviewer comment:**

A robust validation of medical AI requires a blinded, multi-reader study. Human experts must evaluate the generated impressions against raw imaging findings using Likert scales, assessing clinical accuracy, omission of critical findings, and presence of dangerous hallucinations. The absence of an independent, human-driven validation phase renders the study clinically irrelevant, demonstrating merely a statistical illusion.

**Response:**

We agree that blinded expert review is the appropriate next step for determining clinical usefulness and safety. Our study was not designed as a clinical validation study and did not include radiologist scoring of generated impressions against the source findings or imaging. Accordingly, we do not claim clinical equivalence, diagnostic safety, or deployment readiness.

To address this concern, we revised the manuscript to state more explicitly that the present work is a retrospective benchmarking study of textual alignment, not a human validation study. We now emphasize in the Abstract, Discussion, and Limitations that automated metrics such as ROUGE and BERTScore evaluate similarity to the reference impression, but do not establish clinical accuracy, completeness, or safety. We also added a qualitative review of the lowest-performing cases to better characterize failure modes, but we agree that this does not replace blinded multi-reader evaluation.

**Reviewer comment:**

The manuscript does not include a flow diagram outlining the inclusion and exclusion criteria used in data extraction. CTPA imaging is often compromised in acute stroke or pulmonary embolism protocols. Patients in the emergency department frequently exhibit agitation, non-compliance, dyspnea, or have significant indwelling hardware (e.g., pacemakers, central venous catheters), resulting in substantial motion and beam-hardening artifacts that obscure the pulmonary vasculature.

**Response:**

In the present study the cohort was defined directly from the reporting database using administrative eligibility criteria rather than image-level technical screening. Specifically, we included CTPA reports performed within our health system during the prespecified study period and limited the dataset to cases with research authorization and with both findings and impression sections available for analysis. We did not exclude cases based on image quality.

In the Methods:

*“We included CTPA reports from Mayo Clinic sites (Rochester, Arizona, Florida, and the Mayo Clinic Health System) performed between April 30, 2023, and June 17, 2024. Data were accessed for research purposes between March 15, 2025, and April 10, 2025. The authors had access to identifiable patient information during data extraction, which was handled within institutional firewalls and only for the analysis.*

*In total, 11,998 reports (10,681 unique patients) were analyzed. Eligible reports contained both findings and impression sections and included both structured and unstructured report formats.. Reports were randomly split at the report level: 95% (n=11,399) formed the retrieval bank and 5% (n=599) were held out for testing. Because the split was performed at the report rather than patient level, patients with multiple examinations could contribute more than one report across the overall dataset. The reference impressions served as the ground truth for evaluation.”*

**Reviewer comment:**

The authors present mean ROUGE and BERTScore improvements as absolute ranges (e.g., LLaMA ROUGE-1 F1 rose from 0.25-0.37 to 0.37-0.50). The report omits the 95% Confidence Intervals (CIs) for these point estimates.

Point estimates and p-values are inadequate in rigorous scientific reporting. A p-value indicates the presence of a difference but provides no information on its magnitude, clinical significance, or precision. The 95% confidence intervals are essential as they define the limits of the "worst-case scenario." High variance in model performance may cause the RAG model's confidence interval lower limit to significantly overlap with the zero-shot model's upper limit, making the costly retrieval infrastructure mathematically irrelevant. The lack of precision metrics hinders a serious assessment of the evidence's certainty.

**Response:**

We completely agree that reporting point estimates with p-values alone is incomplete. In response, we calculated and added 95% confidence intervals for all reported mean ROUGE-1, ROUGE-2, ROUGE-L, and BERTScore F1 values across models, temperatures, prompting strategies, and top-k settings. These confidence intervals are now provided in Supplemental Tables 1-8.

**Reviewer comment:**

The manuscript indicates that 5% of the data (n=599) is reserved for testing. The authors exceed certain minimums for machine learning studies but do not provide a priori sample size power analysis. Was the sample size calculated to detect a Minimum Clinically Important Difference in NLP metrics?

**Response:**

Thank you for this important point. Because this was a retrospective study based on an available dataset, the sample size was not determined by a prospective power calculation. In addition, there is currently no established minimum clinically important difference for ROUGE or BERTScore in the specific setting of radiology impression generation, which limits the feasibility of a clinically anchored a priori power analysis for these automated text-similarity metrics. The study was designed as a comparative evaluation of inference strategies on a fixed held-out test set. To strengthen the statistical reporting, we have added 95% confidence intervals for all mean scores and retained paired Wilcoxon signed-rank testing for strategy comparisons. We agree that future prospective studies using human-rated clinical endpoints would allow more appropriate clinically grounded sample size planning.

**Reviewer comment:**

The authors determine that the optimal configuration involves retrieving ten prior cases (k=10) and operating the LLM at a temperature of 0. Executing an embedding model on new text necessitates querying a vector database of 11,399 reports through semantic search, utilizing a context window of a 70-billion parameter model (LLaMA 3.1-70B) or performing a network-intensive API call (GPT-4o) with ten examples, followed by deterministic token generation.

In a "Code Stroke" or acute PE scenario, time is critical for tissue viability and patient survival. The authors do not quantify the inference latency of their pipeline. The complex RAG process increases processing time by 45 to 60 seconds per report, creating a significant workflow bottleneck. If the radiologist must audit the generated text word-by-word to confirm the LLM hasn't fabricated an acute finding, the efficiency gains of automation are lost.

**Response:**

Thank you for this important point. We agree that inference latency and workflow feasibility are critical considerations, particularly in time-sensitive settings such as acute PE evaluation. However, these operational characteristics were not measured in the present study, and we did not evaluate end-to-end latency, retrieval overhead, token or compute cost, throughput, or radiologist interaction time. Accordingly, we have revised the manuscript to clarify that our results address text-generation alignment only and should not be interpreted as evidence of workflow efficiency or real-world deployment readiness. We have added this explicitly to the Methods and Discussion/Limitations sections.

In Methods:

*“This study evaluated text-generation performance only; latency, throughput, compute cost, and clinical workflow feasibility were not assessed.”*

In Discussion:

*“Finally, we did not assess latency, throughput, compute cost, or operational feasibility in clinical workflow; therefore, these findings should not be interpreted as evidence of deployment readiness.”*

**Reviewer comment:**

NLP metrics only measure string overlap and contextual embeddings, remaining entirely ignorant of medical fact, negation, and subtle diagnostic Modifiers. The assertion that the model

"improves" impressions is statistically true but clinically unproven and potentially dangerous.

**Response:**

Thank you. We agree with this concern. ROUGE and BERTScore assess textual overlap and semantic similarity, not clinical correctness, safety, or diagnostic validity, and they may fail to capture clinically important negation errors, omissions, or unsupported statements. We have revised the manuscript to avoid overstating the findings and to frame the results more precisely as improved textual alignment with reference impressions rather than improved clinical performance. We also strengthened the Limitations section to state explicitly that the observed gains should not be interpreted as evidence of clinical accuracy, safety, or deployment readiness.

**Reviewer comment:**

The manuscript displays several missed opportunities. The authors did not conduct a true multi-reader radiologist study. The lack of human expert evaluation of clinical accuracy against original imaging led to the underutilization of a valuable dataset for superficial NLP benchmarking. The authors did not define the limits of their tool. Analyzing the lowest 10% of generated impressions is essential for identifying model failures. Did it fail due to complex ICU reports? Did it fail because of conflicting results? Clinicians derive more insights from an algorithm's failures than from its successes.

The authors compared RAG to zero-shot and fixed few-shot prompts. The chance to compare the complex RAG pipeline with the true clinical standard of a human radiologist's time and accuracy was overlooked. “Large language models (LLMs) excel in various natural language processing tasks.”

**Response:**

Thank you. We agree that these are important limitations and opportunities for future work. We did not perform a multi-reader radiologist study or direct human evaluation against source imaging, so this work should be interpreted as a benchmarking study of text generation alignment rather than a clinical accuracy study. We have revised the manuscript to make this distinction more explicit and to avoid claims beyond the scope of the evaluation.

To address interpretability of failure modes, we added a qualitative review of the lowest-performing generated impressions and categorized the major error types. This analysis is now summarized in the Results and provided in the Supplement, where it shows that failures were not uniform across models. GPT-4o low-scoring cases were most often due to wrong prioritization/redundant detail, whereas LLaMA low-scoring cases were frequently truncated or malformed outputs, with additional hallucinations, omissions, and overinterpretation also observed. We agree that these failure patterns are clinically more informative than aggregate metric gains alone, and we added this analysis for that reason.

We also agree that comparison with real clinical workflow is important. However, this study was not designed to compare LLM-assisted generation against radiologist interpretation time, accuracy, or reporting efficiency. We have clarified this in the Discussion and now state explicitly that human reader studies, source-image-based evaluation, and workflow comparison against radiologist performance are necessary next steps.

We would like to thank you again for the insightful comments and for your time and effort in reviewing our manuscript.
