# Supplementary material for: Case-matched retrieval improves textual alignment of LLM-generated radiology impressions
Source: PLoS One. 2026 Jul 31;21(7):e0354688. doi: 10.1371/journal.pone.0354688 (PMC13426976; doi:10.1371/journal.pone.0354688)
Supplement: Peer review history

# Peer review: Decision Letter 0

**DOI:** 10.1371/journal.pone.0354688.r001

**Authors:** Krit Pongpirul

26 Feb 2026

PONE-D-25-66963

Case-Matched Retrieval Improves LLM-Generated Radiology Impressions

PLOS One

Dear Dr. Korfiatis,

Thank you for submitting your manuscript to PLOS ONE. After careful consideration, we feel that it has merit but does not fully meet PLOS ONE’s publication criteria as it currently stands. Therefore, we invite you to submit a revised version of the manuscript that addresses the points raised during the review process.

Please submit your revised manuscript by Apr 12 2026 11:59PM. If you will need more time than this to complete your revisions, please reply to this message or contact the journal office at plosone@plos.org. When you're ready to submit your revision, log on to https://www.editorialmanager.com/pone/ and select the 'Submissions Needing Revision' folder to locate your manuscript file.

Please include the following items when submitting your revised manuscript:

- A letter that responds to each point raised by the academic editor and reviewer(s). You should upload this letter as a separate file labeled 'Response to Reviewers'.
- A marked-up copy of your manuscript that highlights changes made to the original version. You should upload this as a separate file labeled 'Revised Manuscript with Track Changes'.
- An unmarked version of your revised paper without tracked changes. You should upload this as a separate file labeled 'Manuscript'.

If you would like to make changes to your financial disclosure, please include your updated statement in your cover letter. Guidelines for resubmitting your figure files are available below the reviewer comments at the end of this letter.

If applicable, we recommend that you deposit your laboratory protocols in protocols.io to enhance the reproducibility of your results. Protocols.io assigns your protocol its own identifier (DOI) so that it can be cited independently in the future. For instructions see: https://journals.plos.org/plosone/s/submission-guidelines#loc-laboratory-protocols. Additionally, PLOS ONE offers an option for publishing peer-reviewed Lab Protocol articles, which describe protocols hosted on protocols.io. Read more information on sharing protocols at https://plos.org/protocols?utm_medium=editorial-email&utm_source=authorletters&utm_campaign=protocols.

We look forward to receiving your revised manuscript.

Kind regards,

Krit Pongpirul, MD, MPH, PhD.

Academic Editor

PLOS One

Journal Requirements:

When submitting your revision, we need you to address these additional requirements.

1. Please ensure that your manuscript meets PLOS ONE's style requirements, including those for file naming. The PLOS ONE style templates can be found at

https://journals.plos.org/plosone/s/file?id=wjVg/PLOSOne_formatting_sample_main_body.pdf and https://journals.plos.org/plosone/s/file?id=ba62/PLOSOne_formatting_sample_title_authors_affiliations.pdf

2. For studies involving third-party data, we encourage authors to share any data specific to their analyses that they can legally distribute. PLOS recognizes, however, that authors may be using third-party data they do not have the rights to share. When third-party data cannot be publicly shared, authors must provide all information necessary for interested researchers to apply to gain access to the data. (https://journals.plos.org/plosone/s/data-availability#loc-acceptable-data-access-restrictions)

For any third-party data that the authors cannot legally distribute, they should include the following information in their Data Availability Statement upon submission:

1) A description of the data set and the third-party source

2) If applicable, verification of permission to use the data set

3) Confirmation of whether the authors received any special privileges in accessing the data that other researchers would not have

4) All necessary contact information others would need to apply to gain access to the data

3. Your ethics statement should only appear in the Methods section of your manuscript. If your ethics statement is written in any section besides the Methods, please delete it from any other section.

4. Please include captions for your Supporting Information files at the end of your manuscript, and update any in-text citations to match accordingly. Please see our Supporting Information guidelines for more information: http://journals.plos.org/plosone/s/supporting-information.

If the reviewer comments include a recommendation to cite specific previously published works, please review and evaluate these publications to determine whether they are relevant and should be cited. There is no requirement to cite these works unless the editor has indicated otherwise.

Additional Editor Comments:

The manuscript addresses an important and timely problem in medical informatics—namely, reducing the administrative burden on radiologists through inference-time strategies such as retrieval-augmented generation (RAG). The study benefits from a large, real-world dataset of nearly 12,000 CT pulmonary angiography reports, a pragmatic focus on inference-time adaptation rather than model retraining, and evaluation across both proprietary and open-source large language models. These elements place the work squarely within the scope of PLOS ONE.

However, the reviewer raises several methodological and reporting concerns that affect the interpretation and strength of the conclusions. In particular, the following points require attention:

1. The current manuscript overstates its findings by implying improvement in clinical quality or diagnostic validity of radiology impressions. The reported results demonstrate improved textual or stylistic alignment with reference impressions using automated NLP metrics. Please revise the language throughout the manuscript (including title, abstract, results, and conclusions) to clearly and consistently reflect this distinction and to avoid claims of clinical accuracy, safety, or effectiveness.

2. The exclusive reliance on automated similarity metrics (ROUGE and BERTScore) must be more explicitly framed as a benchmarking approach rather than a proxy for clinical correctness. Please substantially strengthen the limitations section to clarify:

- That these metrics do not assess diagnostic accuracy, negation errors, omissions, or harmful hallucinations.

- That no claims are made regarding clinical safety or real-world deployment without human verification.

3. The manuscript should include appropriate measures of precision. Please report 95% confidence intervals (or equivalent uncertainty estimates) for primary outcome metrics and clarify variability across test cases. Point estimates and p-values alone are insufficient for assessing the robustness of the observed differences.

4. To improve interpretability and address concerns about superficial syntactic overlap, please include an analysis of model failures. At minimum, we recommend a qualitative or semi-quantitative review of the lowest-performing subset of generated impressions (e.g., lowest decile by ROUGE-L), with categorization of error types (e.g., benign paraphrasing, omission, negation error, hallucinated findings).

5. Please consider adding:

- A clear description or diagram of cohort inclusion/exclusion and data flow.

- Clarification regarding patient-level versus report-level splitting and its implications.

- A more explicit statement regarding computational and workflow considerations, noting that latency and operational feasibility were not evaluated in this study.

[Note: HTML markup is below. Please do not edit.]

Reviewers' comments:

Reviewer's Responses to Questions

**Comments to the Author**

1. Is the manuscript technically sound, and do the data support the conclusions?

The manuscript must describe a technically sound piece of scientific research with data that supports the conclusions. Experiments must have been conducted rigorously, with appropriate controls, replication, and sample sizes. The conclusions must be drawn appropriately based on the data presented.

Reviewer #1: Yes

Reviewer #2: Partly

**********

2. Has the statistical analysis been performed appropriately and rigorously?

Reviewer #1: Yes

Reviewer #2: No

**********

3. Have the authors made all data underlying the findings in their manuscript fully available?

The PLOS Data policy requires authors to make all data underlying the findings described in their manuscript fully available without restriction, with rare exception (please refer to the Data Availability Statement in the manuscript PDF file). The data should be provided as part of the manuscript or its supporting information, or deposited to a public repository. For example, in addition to summary statistics, the data points behind means, medians and variance measures should be available. If there are restrictions on publicly sharing data—e.g. participant privacy or use of data from a third party—those must be specified.

Reviewer #1: Yes

Reviewer #2: No

**********

4. Is the manuscript presented in an intelligible fashion and written in standard English?

PLOS ONE does not copyedit accepted manuscripts, so the language in submitted articles must be clear, correct, and unambiguous. Any typographical or grammatical errors should be corrected at revision, so please note any specific errors here.

Reviewer #1: Yes

Reviewer #2: Yes

**********

5. Review Comments to the Author

Please use the space provided to explain your answers to the questions above. You may also include additional comments for the author, including concerns about dual publication, research ethics, or publication ethics. (Please upload your review as an attachment if it exceeds 20,000 characters)

Reviewer #1: This manuscript presents a technically sound and clearly scoped evaluation of case‑matched retrieval for improving LLM‑generated radiology impressions. The methods are appropriate and the analyses are transparent. Limitations have also beem acknowledged.

Reviewer #2: This manuscript evaluates the effectiveness of Retrieval-Augmented Generation (RAG) in improving the quality of radiology impressions produced by LLMs. The authors analyze a retrospective cohort of 11,998 CTPA reports, comparing a dynamic, case-matched retrieval few-shot prompting strategy with zero-shot and fixed random few-shot baselines.

The clinical issue discussed in this manuscript is significant to contemporary healthcare; however, the research execution is severely flawed. The manuscript employs a validation strategy that depends on superficial syntactical mimicry instead of clinical accuracy, making the findings mathematically questionable and diagnostically ineffective. The authors replaced thorough human evaluation with automated n-gram overlap metrics, resulting in a simplified narrative that overlooks the complexities of real-world acute care workflows. The manuscript demonstrates technical deficiencies and does not comply with essential statistical reporting standards, particularly lacking 95% Confidence Intervals (CIs). The assertion that RAG "improves" impressions is an exaggeration, as the data only demonstrates increased syntactical overlap, not clinical accuracy or safety.

It is essential to identify the strengths and logical merits within the manuscript. The administrative load on radiologists is untenable. Exploring inference-time strategies such as RAG to automate the synthesis of findings into impressions addresses a significant bottleneck in real-world applications and is a legitimate focus of applied medical informatics. The use of a dataset with nearly 12,000 CTPA reports from over 10,000 unique patients is commendable. This offers a substantial and anatomically varied repository of pulmonary vascular pathology for the embedding database, significantly surpassing the limited volumes observed in many early pilot AI studies. The shift from expensive, resource-intensive fine-tuning methods (e.g., LoRA) to dynamic, inference-time RAG is pragmatic. The authors assert that RAG enables model adaptation to specific institutional reporting styles and updated clinical guidelines by updating the vector database, thus avoiding the significant overhead of continuous neural network retraining. This evaluation of a proprietary model (GPT-4o) and an open-source model (LLaMA 3.1-70B) facilitates a robust comparative analysis. This study examines the performance of open-source models, favored in healthcare for patient data privacy, compared to leading commercial APIs.

This study's methodological framework is based on critical assumptions. The authors assess the effectiveness of their RAG pipeline using automated natural language processing metrics: ROUGE and BERTScore F1. This dependence on automated metrics represents a superficial validation that obscures the actual clinical effectiveness of the algorithms.

In a CT Pulmonary Angiography report, these metrics are diagnostically inadequate and potentially negligent. A generative model could produce the impression: "There is no evidence of a central pulmonary embolism." If the original radiologist wrote,"There is evidence of a central pulmonary embolism," and the two sentences exhibit nearly identical lexical composition and contextual token clusters, differing solely by one negative modifier. ROUGE and BERTScore would assign a high similarity score to this generated text, overlooking the LLM's hallucination of a false negative that could lead to the patient being denied essential anticoagulation therapy. The ROUGE-1 F1 scores of 0.44 to 0.50 are considered successful; however, the authors do not perform any manual error analysis to identify the nature of the remaining 50% to 56% of the divergent text. Did the model merely employ a synonym (e.g., "atelectasis" for "collapse"), which is clinically benign, or did it fabricate a nonexistent lung nodule?

A robust validation of medical AI requires a blinded, multi-reader study. Human experts must evaluate the generated impressions against raw imaging findings using Likert scales, assessing clinical accuracy, omission of critical findings, and presence of dangerous hallucinations. The absence of an independent, human-driven validation phase renders the study clinically irrelevant, demonstrating merely a statistical illusion.

The manuscript does not include a flow diagram outlining the inclusion and exclusion criteria used in data extraction. CTPA imaging is often compromised in acute stroke or pulmonary embolism protocols. Patients in the emergency department frequently exhibit agitation, non-compliance, dyspnea, or have significant indwelling hardware (e.g., pacemakers, central venous catheters), resulting in substantial motion and beam-hardening artifacts that obscure the pulmonary vasculature.

The authors present mean ROUGE and BERTScore improvements as absolute ranges (e.g., LLaMA ROUGE-1 F1 rose from 0.25-0.37 to 0.37-0.50). The report omits the 95% Confidence Intervals (CIs) for these point estimates.

Point estimates and p-values are inadequate in rigorous scientific reporting. A p-value indicates the presence of a difference but provides no information on its magnitude, clinical significance, or precision. The 95% confidence intervals are essential as they define the limits of the "worst-case scenario." High variance in model performance may cause the RAG model's confidence interval lower limit to significantly overlap with the zero-shot model's upper limit, making the costly retrieval infrastructure mathematically irrelevant. The lack of precision metrics hinders a serious assessment of the evidence's certainty.

The manuscript indicates that 5% of the data (n=599) is reserved for testing. The authors exceed certain minimums for machine learning studies but do not provide a priori sample size power analysis. Was the sample size calculated to detect a Minimum Clinically Important Difference in NLP metrics?

The authors determine that the optimal configuration involves retrieving ten prior cases (k=10) and operating the LLM at a temperature of 0. Executing an embedding model on new text necessitates querying a vector database of 11,399 reports through semantic search, utilizing a context window of a 70-billion parameter model (LLaMA 3.1-70B) or performing a network-intensive API call (GPT-4o) with ten examples, followed by deterministic token generation.

In a "Code Stroke" or acute PE scenario, time is critical for tissue viability and patient survival. The authors do not quantify the inference latency of their pipeline. The complex RAG process increases processing time by 45 to 60 seconds per report, creating a significant workflow bottleneck. If the radiologist must audit the generated text word-by-word to confirm the LLM hasn't fabricated an acute finding, the efficiency gains of automation are lost.

NLP metrics only measure string overlap and contextual embeddings, remaining entirely ignorant of medical fact, negation, and subtle diagnostic Modifiers. The assertion that the model

"improves" impressions is statistically true but clinically unproven and potentially dangerous.

The manuscript displays several missed opportunities. The authors did not conduct a true multi-reader radiologist study. The lack of human expert evaluation of clinical accuracy against original imaging led to the underutilization of a valuable dataset for superficial NLP benchmarking. The authors did not define the limits of their tool. Analyzing the lowest 10% of generated impressions is essential for identifying model failures. Did it fail due to complex ICU reports? Did it fail because of conflicting results? Clinicians derive more insights from an algorithm's failures than from its successes.

The authors compared RAG to zero-shot and fixed few-shot prompts. The chance to compare the complex RAG pipeline with the true clinical standard of a human radiologist's time and accuracy was overlooked. “Large language models (LLMs) excel in various natural language processing tasks.”

**********

6. PLOS authors have the option to publish the peer review history of their article (what does this mean?). If published, this will include your full peer review and any attached files.

If you choose “no”, your identity will remain anonymous but your review may still be made public.

**Do you want your identity to be public for this peer review?**  For information about this choice, including consent withdrawal, please see our Privacy Policy.

Reviewer #1: No

Reviewer #2: No

**********

[NOTE: If reviewer comments were submitted as an attachment file, they will be attached to this email and accessible via the submission site. Please log into your account, locate the manuscript record, and check for the action link "View Attachments". If this link does not appear, there are no attachment files.]

To ensure your figures meet our technical requirements, please review our figure guidelines: https://journals.plos.org/plosone/s/figures

You may also use PLOS’s free figure tool, NAAS, to help you prepare publication quality figures: https://journals.plos.org/plosone/s/figures#loc-tools-for-figure-preparation.

NAAS will assess whether your figures meet our technical requirements by comparing each figure against our figure specifications.

# Author comment: Author response to Decision Letter 1

**DOI:** 10.1371/journal.pone.0354688.r002

14 Apr 2026

Dear Dr. Krit Pongpirul,

We would like to thank you for the opportunity to submit a revised version of our manuscript titled “Case-Matched Retrieval Improves Textual Alignment of LLM-Generated Radiology Impressions”. We would also like to thank the editor and the reviewers for their valuable comments.

Below are our responses to the editor’s and the reviewers’ comments, and the revised manuscript is attached.

Please do not hesitate to contact us regarding any further issue or question.

Editor’s Comments to the Authors:

Editor’s comment:

The manuscript addresses an important and timely problem in medical informatics—namely, reducing the administrative burden on radiologists through inference-time strategies such as retrieval-augmented generation (RAG). The study benefits from a large, real-world dataset of nearly 12,000 CT pulmonary angiography reports, a pragmatic focus on inference-time adaptation rather than model retraining, and evaluation across both proprietary and open-source large language models. These elements place the work squarely within the scope of PLOS ONE.

However, the reviewer raises several methodological and reporting concerns that affect the interpretation and strength of the conclusions. In particular, the following points require attention:

Response:

Thank you, we appreciate the comment. We revised the manuscript throughout.

Editor’s comment:

The current manuscript overstates its findings by implying improvement in clinical quality or diagnostic validity of radiology impressions. The reported results demonstrate improved textual or stylistic alignment with reference impressions using automated NLP metrics. Please revise the language throughout the manuscript (including title, abstract, results, and conclusions) to clearly and consistently reflect this distinction and to avoid claims of clinical accuracy, safety, or effectiveness.

Response:

Thank you, we agree. We revised the manuscript throughout to better distinguish textual alignment with reference impressions from clinical accuracy or diagnostic validity. We now describe the main finding as improved performance on automated text-similarity metrics and explicitly note that these metrics do not establish clinical correctness and that radiologist review remains necessary.

Revised title:

“Case-Matched Retrieval Improves Textual Alignment of LLM-Generated Radiology Impressions”

In the Abstract:

“Results

Dynamic retrieval-based few-shot prompting outperformed zero-shot and fixed few-shot prompting across all configurations (all p<0.05). The highest scores were observed at temperature 0 and k=10. ROUGE-1 F1 increased to 0.44–0.47 for GPT-4o and 0.37–0.50 for LLaMA, versus 0.35–0.37 and 0.25–0.37, respectively, in zero-shot prompting. Lower temperature and larger k were associated with higher similarity scores.

Conclusions

Dynamic, case-matched retrieval improved alignment of LLM-generated CTPA impressions with reference impressions on automated text-similarity metrics. Scores remained moderate, and radiologists’ verification is still required before clinical deployment.”

In the Discussion:

“This study has several limitations. It was retrospective, limited to a single exam type (CTPA), and evaluated only two LLMs. In addition, we did not perform human evaluation of the generated impressions, so we cannot determine whether higher similarity scores corresponded to better clinical quality, correctness, or safety. Evaluation relied exclusively on automated similarity metrics. ROUGE and BERTScore are useful for benchmarking textual overlap and semantic similarity to reference impressions, but they are not measures of diagnostic accuracy or clinical correctness. They do not reliably detect negation errors, clinically important omissions, unsupported statements, or harmful hallucinations. Accordingly, the improvements reported here should be interpreted as gains in textual alignment with reference impressions rather than evidence of clinical safety, diagnostic validity, or readiness for real-world deployment. We also did not perform a patient-level split analysis, which may affect independence between the retrieval bank and test set and should be addressed in future work.

In conclusion, dynamic, case-matched retrieval consistently improved alignment of LLM-generated CTPA impressions with reference impressions on automated text-similarity metrics. The largest gains were seen at temperature 0 and k=10. Further work with human evaluation will be needed to determine whether these improvements translate to clinically meaningful gains.”

Editor’s comment:

The exclusive reliance on automated similarity metrics (ROUGE and BERTScore) must be more explicitly framed as a benchmarking approach rather than a proxy for clinical correctness. Please substantially strengthen the limitations section to clarify:

- That these metrics do not assess diagnostic accuracy, negation errors, omissions, or harmful hallucinations.

- That no claims are made regarding clinical safety or real-world deployment without human verification.

Response:

We completely agree and revised the limitation paragraph:

“This study has several limitations. It was retrospective, limited to a single exam type (CTPA), and evaluated only two LLMs. In addition, we did not perform human evaluation of the generated impressions, so we cannot determine whether higher similarity scores corresponded to better clinical quality, correctness, or safety. Evaluation relied exclusively on automated similarity metrics. ROUGE and BERTScore are useful for benchmarking textual overlap and semantic similarity to reference impressions, but they are not measures of diagnostic accuracy or clinical correctness. They do not reliably detect negation errors, clinically important omissions, unsupported statements, or harmful hallucinations. Accordingly, the improvements reported here should be interpreted as gains in textual alignment with reference impressions rather than evidence of clinical safety, diagnostic validity, or readiness for real-world deployment. We also did not perform a patient-level split analysis, which may affect independence between the retrieval bank and test set and should be addressed in future work.”

Editor’s comment:

The manuscript should include appropriate measures of precision. Please report 95% confidence intervals (or equivalent uncertainty estimates) for primary outcome metrics and clarify variability across test cases. Point estimates and p-values alone are insufficient for assessing the robustness of the observed differences.

Response:

Thank you. We added 95% confidence intervals for mean metric values across all supplemental performance tables and clarified this in the Methods section.

Editor’s comment:

To improve interpretability and address concerns about superficial syntactic overlap, please include an analysis of model failures. At minimum, we recommend a qualitative or semi-quantitative review of the lowest-performing subset of generated impressions (e.g., lowest decile by ROUGE-L), with categorization of error types (e.g., benign paraphrasing, omission, negation error, hallucinated findings).

Response:

We completely agree. we added a qualitative failure analysis of the lowest-performing generated impressions. Specifically, we reviewed the 50 lowest ROUGE-L F1 outputs for each model (overall 100 impressions) and categorized error types as wrong prioritization/redundant details, truncated or malformed output, hallucinated findings, overinterpretation/unsupported recommendations, omission, benign rephrasing, or other.

In Methods:

“We also performed qualitative analysis on a subset of cases to assess whether low similarity scores reflected superficial wording differences or clinically important errors, the 50 lowest-scoring generated impressions by ROUGE-L F1 for each model (overall 100 impressions) were evaluated by a radiologist. Error categories included wrong prioritization/redundant details, truncated or malformed output, hallucinated findings, overinterpretation/unsupported recommendations, omission, benign rephrasing, and other.”

In Results:

“Review of the 50 lowest-scoring impressions per model showed different error profiles (Supplemental Tables 9–12). GPT-4o failures were dominated by wrong prioritization or redundant details (37/50, 74%), whereas LLaMA 3.1-70B failures were dominated by truncated or malformed output (34/50, 68%). Hallucinations were observed in both models (GPT-4o: 5/50, 10%; LLaMA 3.1-70B: 9/50, 18%), while omissions were less common (GPT-4o: 3/50, 6%; LLaMA 3.1-70B: 4/50, 8%). Representative examples are provided in Supplemental Table 12.”

In the Supplement, we added the following tables: Supplemental Table 9 - Distribution of Lowest Scoring Impressions by Error Type per Model; Supplemental Table 10 - Distribution by Model and Strategy; Supplemental Table 11 - Distribution by Model and Top-k; Supplemental Table 12 - Representative low-scoring cases with assigned error category

Editor’s comment:

Please consider adding:

- A clear description or diagram of cohort inclusion/exclusion and data flow.

- Clarification regarding patient-level versus report-level splitting and its implications.

- A more explicit statement regarding computational and workflow considerations, noting that latency and operational feasibility were not evaluated in this study

Response:

Thank you for these helpful suggestions. We revised the manuscript accordingly.

We clarified cohort assembly and data flow in the Methods and Figure 1 legend. Specifically, we now state that 11,998 CTPA reports from 10,681 unique patients met study inclusion during the specified time window, and that these reports were randomly divided into a retrieval bank (n=11,399) and held-out test set (n=599). We also revised Figure 1 to better depict the study data flow from cohort identification to retrieval-bank construction, test-set selection, retrieval of top-k similar reports, and impression generation.

We clarified that the split was performed at the report level, not the patient level. We added text noting that multiple reports from the same patient could be included, which may reduce strict independence between retrieval and test data compared with a patient-level split. We now state this more explicitly in Methods and Limitations.

We added an explicit statement that this study focused on impression-generation quality under different retrieval configurations and did not evaluate latency, computational burden, throughput, implementation complexity, or real-world workflow feasibility, and that findings should be interpreted as benchmarking textual alignment rather than any indication for impact on clinical deployment.

Reviewers’ Comments to Authors:

Reviewer 1:

Reviewer comment:

This manuscript presents a technically sound and clearly scoped evaluation of case‑matched retrieval for improving LLM‑generated radiology impressions. The methods are appropriate and the analyses are transparent. Limitations have also been acknowledged.

Response:

Thank you, we appreciate the comment, and the time and effort in reviewing our manuscript.

Reviewer 2:

Reviewer comment:

This manuscript evaluates the effectiveness of Retrieval-Augmented Generation (RAG) in improving the quality of radiology impressions produced by LLMs. The authors analyze a retrospective cohort of 11,998 CTPA reports, comparing a dynamic, case-matched retrieval few-shot prompting strategy with zero-shot and fixed random few-shot baselines.

The clinical issue discussed in this manuscript is significant to contemporary healthcare; however, the research execution is severely flawed. The manuscript employs a validation strategy that depends on superficial syntactical mimicry instead of clinical accuracy, making the findings mathematically questionable and diagnostically ineffective. The authors replaced thorough human evaluation with automated n-gram overlap metrics, resulting in a simplified narrative that overlooks the complexities of real-world acute care workflows. The manuscript demonstrates technical deficiencies and does not comply with essential statistical reporting standards, particularly lacking 95% Confidence Intervals (CIs). The assertion that RAG "improves" impressions is an exaggeration, as the data only demonstrates increased syntactical overlap, not clinical accuracy or safety.

Response:

We thank the reviewer for the careful critique. We agree that automated text-similarity metrics do not establish clinical accuracy, safety, or readiness for acute-care deployment, and we have revised the manuscript to make this limitation more explicit throughout. Our intent was to evaluate textual alignment of generated impressions with reference radiologist impressions under different inference strategies, not to claim diagnostic correctness or clinical equivalence.

In response to this comment, we made several changes. We revised the title, abstract, results, discussion, and conclusions to consistently frame the findings as improved textual alignment rather than improved clinical quality or safety.

We added 95% confidence intervals for all mean metric values in all Supplementary Tables to address statistical reporting completeness.

We expanded the limitations and discussion sections to state explicitly that ROUGE and BERTScore are benchmarking tools for textual overlap and semantic similarity, not measures of diagnostic accuracy, factual correctness, or clinical safety. We also now state clearly that latency, throughput, compute cost, and workflow feasibility were not evaluated.

To address the concern that overlap-based metrics may reflect superficial syntactic mimicry, we also added a failure analysis of the lowest-performing subset of impressions. This qualitative/semi-quantitative review showed that low scores were associated with clinically relevant failure modes, including omission, hallucination, overinterpretation, wrong prioritization of details, and truncated or malformed output, rather than simple stylistic variation alone. These additions are intended to improve interpretability and to better delineate what the present study does and does not show.

Reviewer comment:

It is essential to identify the strengths and logical merits within the manuscript. The administrative load on radiologists is untenable. Exploring inference-time strategies such as RAG to automate the synthesis of findings into impressions addresses a significant bottleneck in real-world applications and is a legitimate focus of applied medical informatics. The use of a dataset with nearly 12,000 CTPA reports from over 10,000 unique patients is commendable. This offers a substantial and anatomically varied repository of pulmonary vascular pathology for the embedding database, significantly surpassing the limited volumes observed in many early pilot AI studies. The shift from expensive, resource-intensive fine-tuning methods (e.g., LoRA) to dynamic, inference-time RAG is pragmatic. The authors assert that RAG enables model adaptation to specific institutional reporting styles and updated clinical guidelines by updating the vector database, thus avoiding the significant overhead of continuous neural network retraining. This evaluation of a proprietary model (GPT-4o) and an open-source model (LLaMA 3.1-70B) facilitates a robust comparative analysis. This study examines the performance of open-source models, favored in healthcare for patient data privacy, compared to leading commercial APIs.

Response:

Thank you, we appreciate the comment.

Reviewer comment:

This study's methodological framework is based on critical assumptions. The authors assess the effectiveness of their RAG pipeline using automated natural language processing metrics: ROUGE and BERTScore F1. This dependence on automated metrics represents a superficial validation that obscures the actual clinical effectiveness of the algorithms.

In a CT Pulmonary Angiography report, these metrics are diagnostically inadequate and potentially negligent. A generative model could produce the impression: "There is no evidence of a central pulmonary embolism." If the original radiologist wrote,"There is evidence of a central pulmonary embolism," an

Attachment Submitted filename: response_letter.docx

# Peer review: Decision Letter 1

**DOI:** 10.1371/journal.pone.0354688.r003

**Authors:** Asif Ekbal

12 Jul 2026

Case-Matched Retrieval Improves Textual Alignment of LLM-Generated Radiology Impressions

PONE-D-25-66963R1

Dear Dr. Korfiatis,

We’re pleased to inform you that your manuscript has been judged scientifically suitable for publication and will be formally accepted for publication once it meets all outstanding technical requirements.

Within one week, you’ll receive an e-mail detailing the required amendments. When these have been addressed, you’ll receive a formal acceptance letter and your manuscript will be scheduled for publication.

An invoice will be generated when your article is formally accepted. Please note, if your institution has a publishing partnership with PLOS and your article meets the relevant criteria, all or part of your publication costs will be covered. Please make sure your user information is up-to-date by logging into Editorial Manager at Editorial Manager® and clicking the ‘Update My Information' link at the top of the page. For questions related to billing, please contact billing support.

If your institution or institutions have a press office, please notify them about your upcoming paper to help maximize its impact. If they’ll be preparing press materials, please inform our press team as soon as possible -- no later than 48 hours after receiving the formal acceptance. Your manuscript will remain under strict press embargo until 2 pm Eastern Time on the date of publication. For more information, please contact onepress@plos.org.

Kind regards,

Asif Ekbal

Academic Editor

PLOS One

Additional Editor Comments (optional):

No further comments.

Reviewers' comments:

Reviewer's Responses to Questions

**Comments to the Author**

1. If the authors have adequately addressed your comments raised in a previous round of review and you feel that this manuscript is now acceptable for publication, you may indicate that here to bypass the “Comments to the Author” section, enter your conflict of interest statement in the “Confidential to Editor” section, and submit your "Accept" recommendation.

Reviewer #1: All comments have been addressed

Reviewer #2: All comments have been addressed

**********

2. Is the manuscript technically sound, and do the data support the conclusions?

The manuscript must describe a technically sound piece of scientific research with data that supports the conclusions. Experiments must have been conducted rigorously, with appropriate controls, replication, and sample sizes. The conclusions must be drawn appropriately based on the data presented.

Reviewer #1: Yes

Reviewer #2: Yes

**********

3. Has the statistical analysis been performed appropriately and rigorously?

Reviewer #1: Yes

Reviewer #2: Yes

**********

4. Have the authors made all data underlying the findings in their manuscript fully available?

The PLOS Data policy requires authors to make all data underlying the findings described in their manuscript fully available without restriction, with rare exception (please refer to the Data Availability Statement in the manuscript PDF file). The data should be provided as part of the manuscript or its supporting information, or deposited to a public repository. For example, in addition to summary statistics, the data points behind means, medians and variance measures should be available. If there are restrictions on publicly sharing data—e.g. participant privacy or use of data from a third party—those must be specified.

Reviewer #1: Yes

Reviewer #2: Yes

**********

5. Is the manuscript presented in an intelligible fashion and written in standard English?

PLOS ONE does not copyedit accepted manuscripts, so the language in submitted articles must be clear, correct, and unambiguous. Any typographical or grammatical errors should be corrected at revision, so please note any specific errors here.

Reviewer #1: Yes

Reviewer #2: Yes

**********

6. Review Comments to the Author

Please use the space provided to explain your answers to the questions above. You may also include additional comments for the author, including concerns about dual publication, research ethics, or publication ethics. (Please upload your review as an attachment if it exceeds 20,000 characters)

Reviewer #1: comments are addressed well from my perspective. I have no additional comments for improvement of the work.

Reviewer #2: I praise the authors for how carefully and professionally they revised this manuscript. The authors have effectively addressed all concerns from the initial review, showing a strong commitment to scientific accuracy.

The following improvements have enhanced the quality and reliability of this paper:

The title has been revised, and claims throughout the manuscript have been moderated from "clinical improvement" to "textual alignment," effectively defining the study's scope. This avoids over-interpretation of NLP metrics by the clinical community.

The inclusion of radiologist-driven error analysis on the 100 lowest-scoring impressions is an excellent enhancement. People who read this and want to do more research will find it very helpful to group failures into categories like truncation, hallucination, and omission. The use of 95% Confidence Intervals makes sure that the RAG pipeline is accurate when it comes to testing its strength.

The authors have effectively converted this benchmarking study into a solid applied medical informatics research work. I have no additional concerns and believe this manuscript is ready for publication in PLOS ONE.

**********

7. PLOS authors have the option to publish the peer review history of their article (what does this mean?). If published, this will include your full peer review and any attached files.

If you choose “no”, your identity will remain anonymous but your review may still be made public.

**Do you want your identity to be public for this peer review?**  For information about this choice, including consent withdrawal, please see our Privacy Policy.

Reviewer #1: No

Reviewer #2: **Yes:**  Ali Abdi, MD, Tehran University of Medical Sciences

**********

# Editor report: Acceptance letter

**DOI:** 10.1371/journal.pone.0354688.r004

**Authors:** Asif Ekbal

PONE-D-25-66963R1

PLOS One

Dear Dr. Korfiatis,

I'm pleased to inform you that your manuscript has been deemed suitable for publication in PLOS One. Congratulations! Your manuscript is now being handed over to our production team.

At this stage, our production department will prepare your paper for publication. This includes ensuring the following:

* All references, tables, and figures are properly cited

* All relevant supporting information is included in the manuscript submission,

* There are no issues that prevent the paper from being properly typeset

You will receive further instructions from the production team, including instructions on how to review your proof when it is ready. Please keep in mind that we are working through a large volume of accepted articles, so please give us a few days to review your paper and let you know the next and final steps.

Lastly, if your institution or institutions have a press office, please let them know about your upcoming paper now to help maximize its impact. If they'll be preparing press materials, please inform our press team within the next 48 hours. Your manuscript will remain under strict press embargo until 2 pm Eastern Time on the date of publication. For more information, please contact onepress@plos.org.

You will receive an invoice from PLOS for your publication fee after your manuscript has reached the completed accept phase. If you receive an email requesting payment before acceptance or for any other service, this may be a phishing scheme. Learn how to identify phishing emails and protect your accounts at https://explore.plos.org/phishing.

If we can help with anything else, please email us at customercare@plos.org.

Thank you for submitting your work to PLOS ONE and supporting open access.

Kind regards,

PLOS ONE Editorial Office Staff

on behalf of

Dr. Asif Ekbal

Academic Editor

PLOS One
